# Bistability in the Rac1, PAK, and RhoA Signaling Network Drives Actin Cytoskeleton Dynamics and Cell Motility Switches

**DOI:** 10.1016/j.cels.2016.01.003

**Published:** 2016-01-27

**Authors:** Kate M. Byrne, Naser Monsefi, John C. Dawson, Andrea Degasperi, Jimi-Carlo Bukowski-Wills, Natalia Volinsky, Maciej Dobrzyński, Marc R. Birtwistle, Mikhail A. Tsyganov, Anatoly Kiyatkin, Katarzyna Kida, Andrew J. Finch, Neil O. Carragher, Walter Kolch, Lan K. Nguyen, Alex von Kriegsheim, Boris N. Kholodenko

**Affiliations:** 1 Systems Biology Ireland, University College Dublin, Belfield, Dublin 4, Ireland; 2 Edinburgh Cancer Research Centre, IGMM, University of Edinburgh, Edinburgh EH4 2XR, UK; 3 Department of Pharmacology and Systems Therapeutics, Icahn School of Medicine at Mount Sinai, New York, NY 10029, USA; 4 Institute of Theoretical and Experimental Biophysics, 142290 Pushchino, Moscow Region, Russia; 5 University of Pennsylvania, Philadelphia, PA 19104, USA; 6 Conway Institute, University College Dublin, Belfield, Dublin 4, Ireland; 7 Co-senior author; 8 School of Medicine and Medical Science, University College Dublin, Belfield, Dublin 4, Ireland; 9 Department of Biochemistry and Molecular Biology, Biomedicine Discovery Institute, Monash University, Melbourne, VIC 3800, Australia

**Keywords:** Rac1, RhoA, cell motility, PAK inhibition, bistable switches, mathematical modeling

## Abstract

Dynamic interactions between RhoA and Rac1, members of the Rho small GTPase family, play a vital role in the control of cell migration. Using predictive mathematical modeling, mass spectrometry-based quantitation of network components, and experimental validation in MDA-MB-231 mesenchymal breast cancer cells, we show that a network containing Rac1, RhoA, and PAK family kinases can produce bistable, switch-like responses to a graded PAK inhibition. Using a small chemical inhibitor of PAK, we demonstrate that cellular RhoA and Rac1 activation levels respond in a history-dependent, bistable manner to PAK inhibition. Consequently, we show that downstream signaling, actin dynamics, and cell migration also behave in a bistable fashion, displaying switches and hysteresis in response to PAK inhibition. Our results demonstrate that PAK is a critical component in the Rac1-RhoA inhibitory crosstalk that governs bistable GTPase activity, cell morphology, and cell migration switches.

## Highlights

• RhoA and Rac1 are linked by a double-negative feedback loop
• A model predicts bistability of the system within a physiological parameter range
• Rac1 and RhoA activity is bistable in response to PAK inhibition
• Actin dynamics, cell morphology, and migration show hysteresis upon PAK inhibition

## Graphical Abstract

**Figure:**
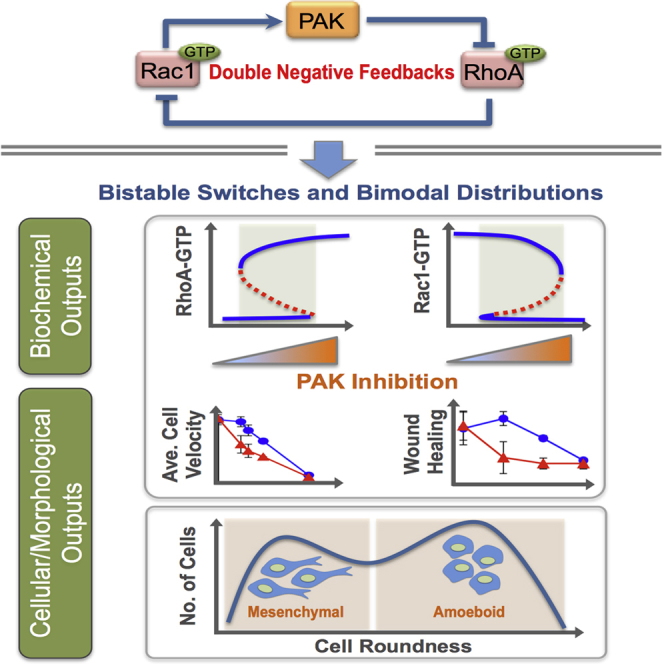

## Teaser

A mathematical model and experiments demonstrate that RhoA, Rac1, actin dynamics, cell morphology, and migration respond in a bistable manner to perturbations.

## Introduction

The members of the Rho family of small guanosine triphosphatase (GTPases), RhoA and Rac1, play crucial roles in a range of cellular functions, including the regulation of the actin cytoskeleton, cell polarity and migration, gene expression, and cell proliferation (Jaffe and Hall, 2005, Takai et al., 2001). Rho GTPases function as molecular switches, cycling between inactive guanosine diphosphate (GDP)-bound (“off”) and active GTP-bound (“on”) states. In their “on” state, Rho GTPases can bind downstream effector proteins, initiating signaling through multiple pathways. The GTPase activation-deactivation cycle is tightly controlled by two opposing enzyme groups, (1) guanine exchange factors (GEFs), which facilitate switching from GDP to guanosine triphosphate (GTP), and (2) GTPase-activating proteins (GAPs), which stimulate GTP to GDP hydrolysis.

Active Rho family GTPases, Rac1 and RhoA, induce the membrane translocation of downstream effectors and trigger their activation, which commonly involves post-translational modifications and conformational changes of bound proteins (Bos et al., 2007, Bustelo et al., 2007). Membrane-bound Rac1-GTP recruits p21-activated kinases (PAKs) by binding to their Cdc42-Rac interactive binding (CRIB) domain. In resting cells, type I PAKs are localized in the cytoplasm as inactive dimers, with the regulatory domain shielding the kinase domain. Rac1 binding induces a conformational change and subsequent activation of PAKs, which then can phosphorylate downstream substrates. The PAKs’ activity converts the local activation of Rho-type GTPases into cell-wide responses (Bokoch, 2003, Zhao and Manser, 2012).

Rac1 and RhoA, along with their fellow Rho GTPase family member Cdc42, work in a coordinated fashion to control cell migration (for reviews, see Burridge and Wennerberg, 2004, Parri and Chiarugi, 2010, Ridley et al., 2003). Rac1 is responsible for driving actin polymerization at the leading edge of a migrating cell, resulting in the formation of lamellipodia, which pushes the cell membrane forward (Nobes and Hall, 1995, Nobes and Hall, 1999, Parri and Chiarugi, 2010, Ridley et al., 1992). Rac1 also promotes focal complex assembly (Nobes and Hall, 1995, Parri and Chiarugi, 2010) and is essential for migration (Nobes and Hall, 1999). RhoA is required for cell adhesion (Nobes and Hall, 1999). It stimulates contractility in cells through myosin light-chain (MLC) phosphorylation, which induces the formation of stress fibers and focal adhesions (Chrzanowska-Wodnicka and Burridge, 1996, Ridley and Hall, 1992). From the perspective of cell morphology, Rac1 and RhoA oppose each other. Although the picture is likely more complicated (see Discussion), canonical descriptions of cell migration place active Rac1 at the migrating cell’s front and active RhoA at its back. Biochemically, Rac1 and RhoA are generally found to interact in mutually antagonistic ways, playing opposing roles in cell migration (Ohta et al., 2006, Sanz-Moreno et al., 2008; reviewed in Guilluy et al., 2011).

Double-negative feedback loops resulting from mutual inhibition can lead to bistability (Kholodenko, 2006). A bistable system can flip between two biochemically distinct steady states; in the proper context, these steady states can promote different cellular phenotypes. Thus, the existence of bistability enables switch-like behaviors in which a graded, analog change in signal inputs could cause abrupt, digital responses in signaling outputs (Ferrell, 2002, Tyson et al., 2003). Bistability has been observed in many biological systems, including the mitogen-activated protein kinase (MAPK) family cascades (Bhalla et al., 2002, Markevich et al., 2004, Markevich et al., 2006, Xiong and Ferrell, 2003) and Cdc2 activation circuit (Pomerening et al., 2003, Sha et al., 2003), which play important roles in diverse cellular functions such as development and memory (Ogasawara and Kawato, 2010). Although it was suggested that mutual inhibition between Rac1 and RhoA may result in bistable activity responses (Jilkine et al., 2007, Symons and Segall, 2009, Tsyganov et al., 2012), this behavior and the consequences for cell migration have not yet been experimentally observed. Here, we combine kinetic modeling and experimentation to demonstrate the existence of bistability in the Rac1-RhoA signaling system of highly motile MDA-MB-231 cells. Model analysis and simulations predict that graded changes in PAK activity induce bistable responses of Rac1 and RhoA activities, which are experimentally validated. Furthermore, the bistable properties of the Rac1-RhoA biochemical circuitry are translated into bistability of the actin dynamics and cell migration.

## Results

### Mathematical Modeling of the Rac1-RhoA Interaction Network

**Figure 1:**
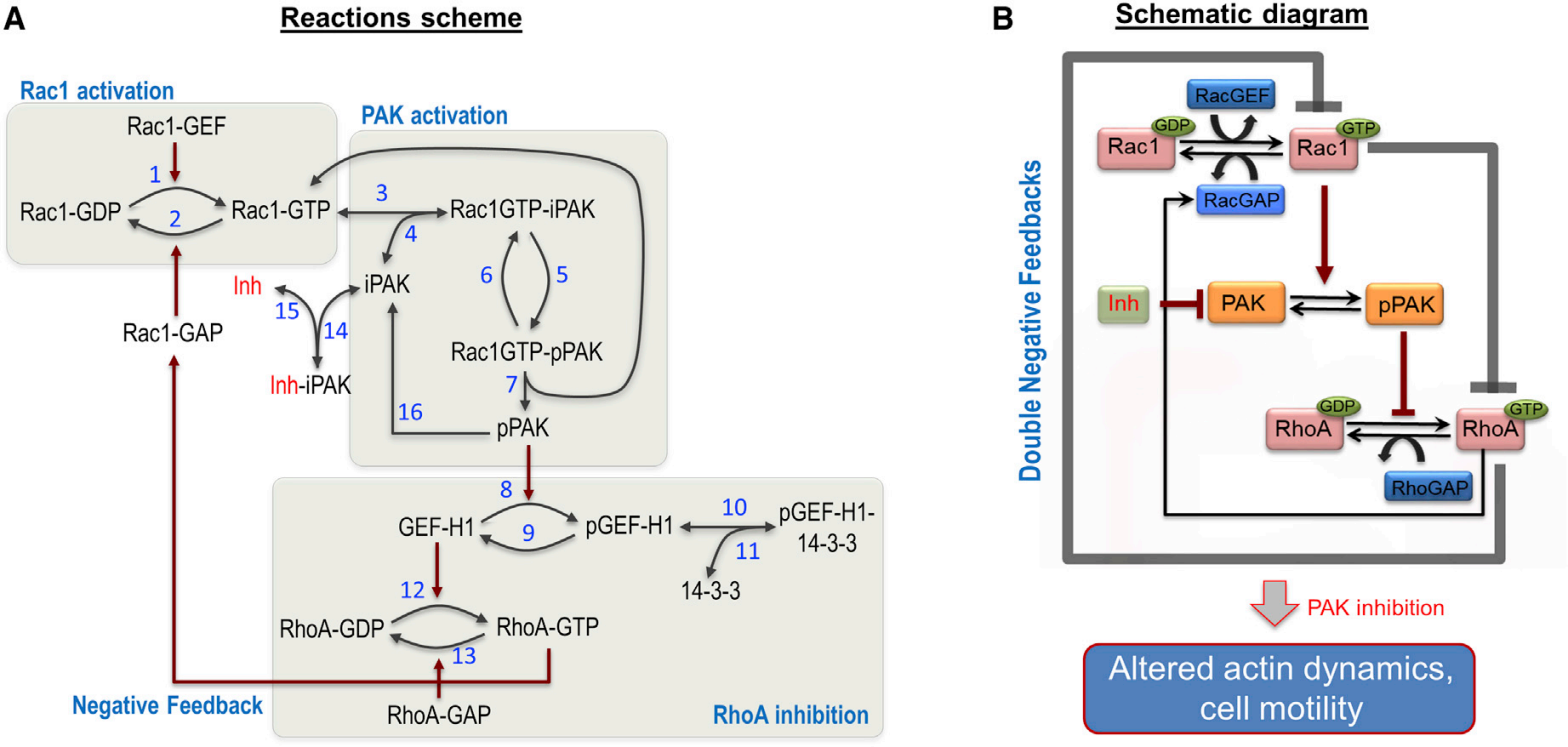
Reaction Scheme and Schematic Interactions of the Rac1-RhoA Network Model (A) Reaction scheme of the kinetic Rac1-RhoA model showing three layers of interconnected regulation of Rac1 and RhoA via PAK (see main text and the Supplemental Information for more details). iPAK and pPAK indicate inactive and active PAK, respectively; we denote PAK Inh as a general but selective PAK inhibitor; and Rac1-GTP, and RhoA-GTP indicate the GTP-bound, active forms of Rac1 and RhoA, respectively. (B) An abstract-level, schematic diagram of the Rac1-RhoA network shows the flow of signaling and highlights the double-negative feedback regulation between Rac1 and RhoA.

To explore the signaling properties of the Rac1-RhoA network, we developed a kinetic model of the network circuitry that captures main molecular events including protein-protein interactions, GTPase activation and deactivation, phosphorylation and dephosphorylation, and feedback regulations. A detailed reaction scheme of the Rac1-RhoA network is given in Figure 1A, while Figure 1B presents a schematic diagram showing the signal flow in the network among Rac1, RhoA, and PAK. The model is formulated as a system of ordinary differential equations using a combination of mass-action and enzyme kinetics laws and is fully described in the Supplemental Information.

Our kinetic model is tailored to MDA-MB-231 cells, where GEF-H1 is one of the dominant RhoA GEFs (Heck et al., 2012, von Thun et al., 2013). The model assumes that Rac1 activates PAK by binding to the PAK CRIB domain, causing a conformational transition of PAK. This exposes PAK’s activation loop, which is subsequently auto-phosphorylated through an intra-molecular mechanism, resulting in full activation of the kinase (Bokoch, 2003, Zhao and Manser, 2012). Activated PAK phosphorylates GEF-H1, a GEF for RhoA, on inactivating inhibitory sites (Zenke et al., 2004). Following phosphorylation, GEF-H1 binds to 14-3-3 protein, a small, phospho-motif binding, dimeric adaptor protein, which causes the GEF-H1 relocation to microtubules (Zenke et al., 2004), where its activity substantially decreases (Krendel et al., 2002). In this way, Rac1 inhibits RhoA activity through PAK, seen as the downward negative regulation from Rac1 to RhoA in Figure 1B.

We have experimentally shown that expression of constitutively active RhoA (RhoAV14) in MDA-MB-231 cells decreases the level of active Rac1 (Figure S1). There are several potential routes for the inhibition of Rac1 by RhoA, including through the regulation of RacGAPs, e.g., ARHGAP22 (Sanz-Moreno et al., 2008) and FilGAP (Saito et al., 2012), which are phosphorylated and activated by the Rho effector kinase ROCK. In our model, we assume that active RhoA deactivates Rac1 via activation of Rac1 GAPs (Figure 1A). This constitutes a negative regulation from RhoA to Rac1, as depicted in Figure 1B, closing a double-negative feedback loop between Rac1 and RhoA.

To determine the abundance of the proteins in the network, we quantified the concentration of the proteins by quantitative mass spectrometry. Several mass spectrometry methods have been developed that use the sum of peptide ion current integrals to estimate absolute protein concentrations. Overall, the error of these methods will be below one order of magnitude and will mostly be within a 2-fold window for abundant proteins (Li et al., 2014, Schwanhäusser et al., 2011). Using a combination of filter-aided sample preparation (Wiśniewski et al., 2009) and the proteome ruler approach (Wiśniewski et al., 2014), we quantified the ∼5,000 most abundant proteins in MDA-MB-231 cells. PAK2 is the only PAK isoform we identified in this dataset. GEF-H1 and p115-RhoGEF are the most highly expressed RhoA GEFs, and Rac1 and RhoA are the most abundant isoforms among the Rac and Rho family in these cells (Tables S5 and S6). p115-RhoGEF is mostly active downstream of G protein-coupled receptors and was consequently omitted from the model. However, GEF-H1 has been shown to be a major contributor toward RhoA activity in this cell line (von Thun et al., 2013). Our model therefore considers only these Rac, Rho, and PAK isoforms and GEF-H1. This makes the model simple enough for efficient analysis, yet it captures the most essential biological interactions.

### Bistable Switch-like Responses in the Modeled Rac1-RhoA System

**Figure 2:**
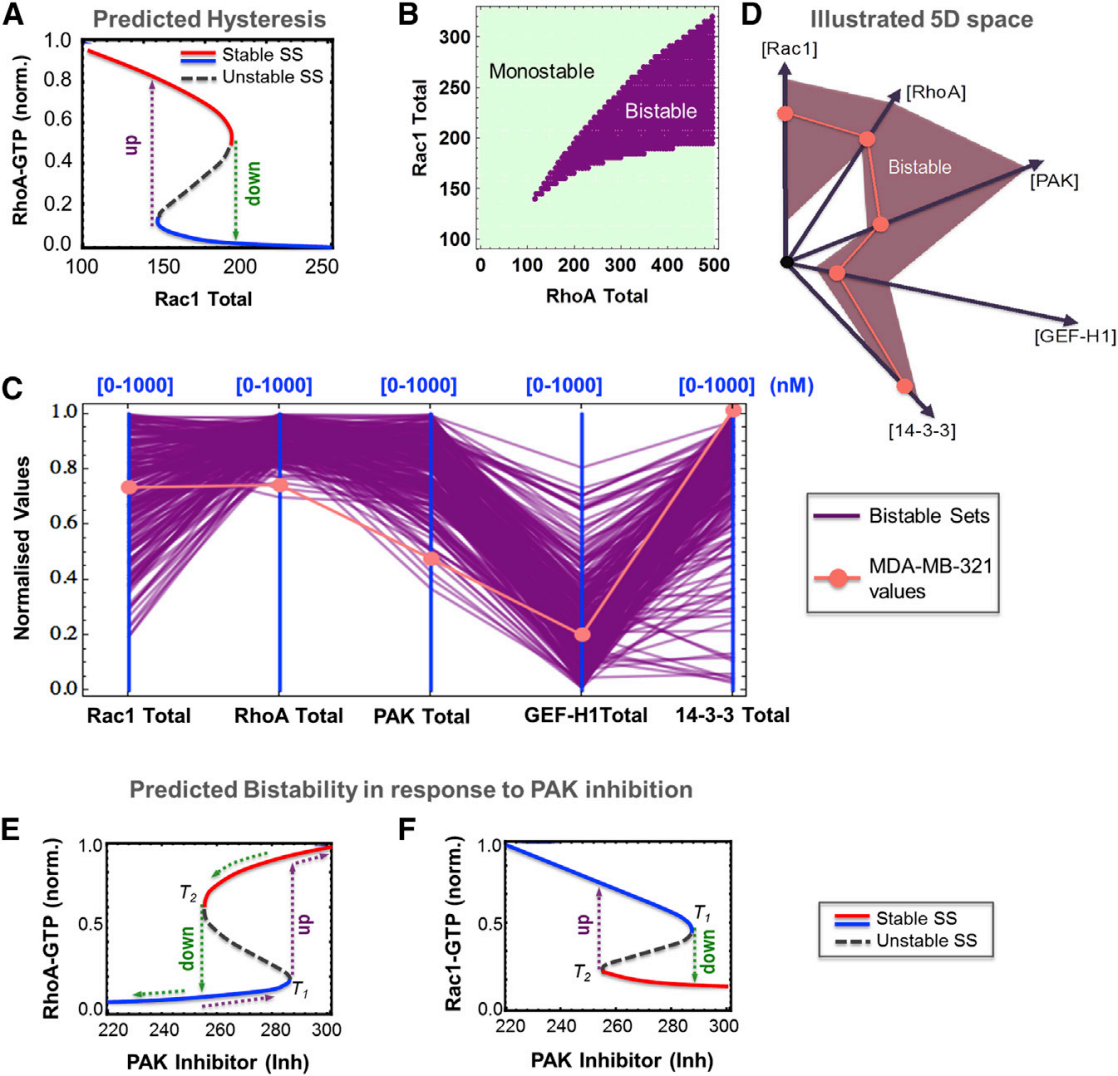
The Rac1-RhoA Double-Negative Feedback Loop Can Result in Bistable Behavior (A) Active RhoA-GTP responses to increasing Rac1 abundance in a bistable manner, resulting in abrupt switches of activity level. SS, Steady State. (B) Two-parameter (2D) bifurcation diagrams showing dependence of bistability on the abundance of Rac1 and RhoA. (C) A parallel coordinate plot showing the bistable parameter sets (purple) obtained by assessing the dynamics of 100,000 sets with randomly sampled Rac1, RhoA, PAK, GEF-H1, and 14-3-3 totals within the indicated ranges (in nanomolars). The values are normalized between 0 and 1. A detailed description of the multi-dimensional dynamic analysis and parallel coordinate representation is given in Nguyen et al. (2015) and Supplemental Experimental Procedures. (D) An intuitive simplified illustration of the bistable region in the corresponding 5D parameter space. (E and F) Simulated bistability and hysteresis for active Rac1 and RhoA in response to increasing the PAK inhibitor level. All simulations were carried out with parameter values given in Table S4.

To determine MDA-MB-231 cell-specific parameters, we measured the expression levels of key proteins incorporated in the model, including Rac1, RhoA, PAK, GEF-H1, and 14-3-3, by quantitative mass spectrometry (Table S5). After populating the model with these values (see Supplemental Information) and carrying out extensive simulations of steady-state dose responses, bistability of the Rac1-RhoA system was observed in a wide parameter range. In the bistable parameter region, Rac1 and RhoA activity levels responded in a switch-like manner to graded changes in levels of various species. For example, Figure 2A shows a model simulation in which the active RhoA-GTP displays a bistable response to the graded increase in the total Rac1 abundance. We can see that active RhoA levels suddenly jump from a high to a low state when Rac1 gradually increases from an initial low abundance (dashed, green line, Figure 2A), while the backward traverse of Rac1, that is, starting at a high abundance and gradually reducing the total amount of Rac1, abruptly pushes active RhoA from a low to a high state at a different, lower Rac1 threshold (dashed, purple line, Figure 2A). A system’s ability to have two quantitatively different thresholds, associated with each of two quantitatively different starting states, defines the so-called hysteresis phenomenon. Hysteresis is a hallmark of bistable dynamics (Kholodenko, 2006).

Although the ability to support bistable dynamics is a feature of this network’s structure, bistable behavior only occurs at certain parameter values. When parameters are changed, bistability can occur or disappear. Such dramatic changes in the system dynamic behavior are called bifurcations. If two parameters are selected, a two-dimensional (2D) plane of these parameters can be conveniently divided into areas where bistability is present or the GTPase network has a single steady state (called monostability). This partitioning of the parameter space, often referred to as 2D bifurcation diagrams, enables one to discern how changes in the abundances of different model species affect the occurrence and existence of bistability, that is, its robustness. Figure 2B displays a 2D bifurcation plot showing the system is bistable over a large region (in purple) of the Rac1 and RhoA abundances at the assumed physiological kinetic parameter values, while the remaining region exhibits monostability (in light green). Next, we investigated the dependence of bistability in more than two dimensions when multiple model parameters are allowed to simultaneously change. This analysis helped us understand the system dynamics in the multi-dimensional parameter space.

To visualize multi-dimensional parameter settings in which bistability is present or absent, we use a software program DYVIPAC (see Supplemental Experimental Procedures and Figure S2, which illustrates the DYVIPAC methodology) (Nguyen et al., 2015). Figure 2C displays the results of multi-dimensional parameter analysis in the form of parallel coordinate plots where the abundances of five model species, Rac1, RhoA, PAK, GEF-H1, and 14-3-3, are allowed to change within physiologically sensible ranges (here, from 0 to 1,000 nM). Systematic sampling of 100,000 sets in this five-dimensional (5D) parameter space for DYVIPAC analysis shows that the system can display either bistable or monostable behavior. For ease of visualization, Figure 2C displays only the bistable sets using the same color, while individually color-coded bistable sets could be seen in Figure S3B alone or with the monostable sets in Figure S3A. Figure 2C indicates that bistability occurs for a large number of parameter sets.

Bistability is found to strongly associate with high RhoA, PAK, and 14-3-3 abundances but low GEF-H1 abundance, while bistability may occur at both high and low Rac1 levels. When we overlay the measured protein levels for MDA-MB-231 cells, we observe that the MDA-MB-231-specific values appear to be within the bistable region (Figure 2C) for the assumed values of kinetic constants. A more intuitive visualization of bistable region in the 5D parameter space is given in Figure 2D. For larger sampling ranges (0 to 5,000 nM) our analysis reveals similar patterns of the bistable region, suggesting that relative rather than absolute values of these species determine the occurrence of bistability (Figure S4). Similar analysis describing the effect of the kinetic parameters on the bistable region (Figures S5 and S6) and the behavior of a comparable dimensionless model (Supplemental Experimental Procedures and Figure S7) demonstrates that this system’s bistable region is wide.

Rac1 and RhoA do not act in isolation inside the cell, but through their immediate effectors, PAK and ROCK, these GTPases regulate cytoskeleton dynamics and cell migration. Because PAK activity can be perturbed experimentally, we next asked how Rac1 and RhoA activity would behave in response to PAK inhibition. To this end, we extended our model to include PAK inhibition, which is described as a reaction in which a PAK inhibitor binds to inactive, unphosphorylated PAK, competing with PAK binding to Rac1-GTP (reactions 14 and 15, Figure 1A) (Deacon et al., 2008, Viaud and Peterson, 2009). Model simulations suggest that active RhoA and Rac1 display bistable, switch-on and switch-off responses, respectively, to increasing PAK inhibitor levels (Figures 2E and 2F). Within a defined range of inhibitor concentration, active Rac1 and RhoA achieved one of two stable steady states (solid lines, Figures 2E and 2F) but could not settle in the intermediate, unstable one (dashed lines, Figures 2E and 2F). At low inhibition levels, RhoA-GTP could display a single low steady-state value. As the inhibitor level increases, active RhoA rises slowly until it reaches a threshold level (*T*_1_) at which it suddenly switches to a high state (purple dashed lines, Figure 2E). In contrast, when the high active RhoA state is initially induced by the high PAK inhibitor levels and then the inhibitor level decreases, active RhoA slowly decreases until it reaches a second, lower threshold (*T*_2_) at which active RhoA drops precipitously and switches to a low state (green dashed line, Figure 2E). Similarly, active Rac1 reacts in a bistable manner to the PAK inhibitor, although in an opposite fashion to RhoA (Figure 2F). A 2D bifurcation analysis further shows that bistability occurs within a bounded range of inhibitor concentration (Figures S8A and S8B). When we vary the levels of the key model species within 2-fold of their measured values in MDA-MB-231, RhoA-GTP and Rac1-GTP continue to exhibit bistable responses to graded PAK inhibition but the switching thresholds are parameter dependent (Figures S8C and S8D). Thus, our model analysis suggests that Rac1 and RhoA activities can display robust bistable responses to PAK inhibition.

### Experimental Validation of Bistable Switches of Rac1 and RhoA Activity Levels

Here, we test model predictions experimentally using the MDA-MB-231 human breast cancer cell line. These experiments rely on chemical inhibition of PAK, because small molecule inhibitors, which are quickly imported and exported from cells, can reach equilibrium within the time frame of the experiments before transcriptional feedback effects take place and the system changes in fundamental ways. We used IPA-3, a chemical inhibitor that specifically targets inactive group 1 PAKs (Deacon et al., 2008, Viaud and Peterson, 2009) by preferentially binding to the inactive conformation of the PAKs’ regulatory domain. Accordingly, it has been observed to inhibit Rac1-mediated PAK activation dose dependently, whereas IPA-3 has a substantially reduced effect on already-active PAK (Deacon et al., 2008, Viaud and Peterson, 2009). To determine the half-life of intercellular IPA-3, we treated the cells with IPA-3 and replaced the media after 20 min of incubation. We then washed the cells with PBS, extracted cellular IPA-3 with methanol, and quantified it by mass spectrometry. We found that the half-life was around 2–5 min (Figure S9). After a 10 min washout, the cellular levels had decreased to 10%–25% of the initial concentration, and IPA-3 levels were undistinguishable from an untreated control after 20 min.

**Figure 3:**
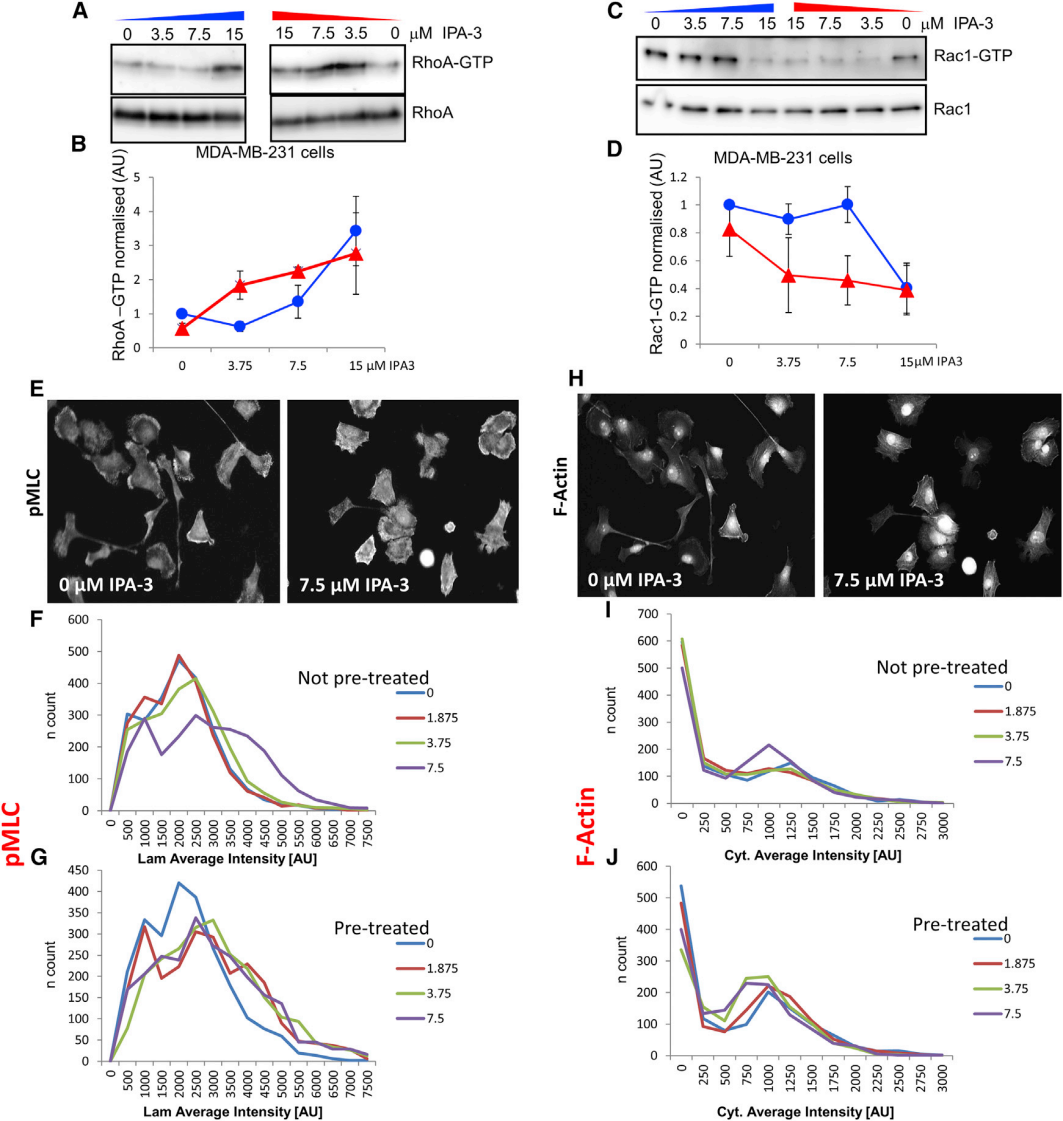
Experimental Validation of RhoA and Rac1 Bistable Switches in MDA-MB-231 Cells (A–D) PAK was inhibited in MDA-MB-231 cells by adding specific inhibitor IPA-3 at the indicated concentrations for 40 min (blue) or by incubating MDA-MB-231 cells with 15 μM IPA-3 for 20 min. The inhibitor was subsequently washed out, and the cells were incubated for an additional 20 min with IPA-3 at the indicated concentrations (red). (A) RhoA-GTP was precipitated with GST-Rhotekin beads and western blotted. (B) Densitometric analysis of three biological replicates. Error bars represent SD. (C) Rac1-GTP was precipitated with GST-PAK-CRIB beads and western blotted. (D) Densitometric analysis of three biological replicates. Error bars represent SD. (E) MDA-MB-231 cells seeded on collagen were treated for 80 min with the indicated concentrations of IPA-3, fixed and stained with an anti-pS19 MLC2 antibody. 20× image. (F) MDA-MB-231 cells seeded on collagen were treated for 80 min with the indicated concentrations of IPA-3, fixed and stained with an anti-pS19 MLC2 antibody. Histogram of single-cell, averaged lamellipodial intensity. (G) MDA-MB-231 cells seeded on collagen were treated for 20 min with 7.5 μM IPA-3. The cells were subsequently washed and treated for an additional 60 min with the indicated concentrations of IPA-3, fixed and stained with an anti-pS19 MLC2 antibody. Histogram of single-cell, averaged lamellipodial intensity. (H) MDA-MB-231 cells seeded on collagen were treated for 80 min with the indicated concentrations of IPA-3, fixed and stained with fluorescent phalloidin. 20× image. (I) MDA-MB-231 cells seeded on collagen were treated for 80 min with the indicated concentrations of IPA-3, fixed and stained with fluorescent phalloidin. Histogram of single-cell, averaged cytoplasmatic intensity. (J) MDA-MB-231 cells seeded on collagen were treated for 20 min with 7.5 μM IPA-2. The cells were subsequently washed and treated for an additional 60 min with the indicated concentrations of IPA-3, fixed and stained with fluorescent phalloidin. Histogram of single-cell, averaged cytoplasmatic intensity.

To determine whether RhoA and Rac1 behave in a bistable manner in MDA-MB-231 cells, we incubated cells that initially have low RhoA-GTP and high Rac1-GTP levels with different, incrementally increasing concentrations of IPA-3, ranging from 0 to 15 μM, for 40 min, as indicated in Figures 3A–3D. Furthermore, to change the initial RhoA and Rac1 activities, we pretreated cells with the highest concentration of IPA-3 (15 μM) for 20 min, locking the system into a high RhoA (Figures 3A and 3B) and low Rac1 (Figures 3C and 3D) activity state. At this point, the inhibitor was washed off, and then incrementally increasing IPA-3 concentrations from 0 to 15 μM were added for an additional 20 min (Figures 3A–3D; see Figure S10 for a workflow diagram of the experiment). Active, GTP-bound RhoA and Rac1 were precipitated with GST-Rhotekin and GST-PAK-CRIB beads, respectively; detected using western blots; and normalized against total RhoA and Rac1 levels (Figures 3A and 3C, respectively). Densitometric analyses of three replicates are shown in Figures 3B and 3D. The blue curves show the responses of active RhoA and Rac1 to incrementally increasing PAK inhibition, and the red curves show their responses to incrementally decreasing PAK inhibition after being initially locked in the state produced by high PAK inhibition (as predicted by simulations in Figures 2E and 2F). Comparing experimental quantifications with model predictions shows that similar switch-like and hysteretic behaviors are present. Loading controls confirm that expression levels of Rac1 and RhoA remain stable over the course of the experiment, thus excluding the possibility that the observed hysteresis is the consequence of altered protein expression in response to PAK inhibition.

### Bistability of Signaling Downstream of RhoA

Having determined that active Rac1 and RhoA behave in a bistable manner in response to PAK inhibition, we wanted to see whether molecular events downstream of the GTPases behave similarly. RhoA-GTP can bind and activate Rho kinase (ROCK), which in turn inhibits MLC phosphatase, as well as directly phosphorylating MLC (Nobes and Hall, 1999). In addition to phosphorylating MLC via ROCK, active RhoA has been shown to increase the formation of actin stress fibers by enhancing actin nucleation and reducing actin depolymerization (Chesarone and Goode, 2009, Ridley and Hall, 1992). Thus, we decided to determine whether IPA-3 could increase stress fiber formation and phosphorylated MLC (pMLC). F-actin stress fibers and pMLC can be imaged by phalloidin staining and by immunofluorescence, respectively. Both readouts can be quantified by high-content microscopy. In addition to quantifying changes at the population level, this approach would allow us to determine F-actin and pMLC at the single-cell level. These data would reveal how the distribution of either marker of RhoA activity was changed in response to PAK inhibition within the cell population.

We seeded MDA-MB-231 cells in collagen-coated 96-well plates and treated the cells with increasing concentrations of IPA-3 for 80 min (non-pretreated). Alternatively, we pretreated the cells with a high concentration of the inhibitor for 20 min, followed by a washout and incubation with increasing concentrations of IPA-3 for an additional 60 min (see Figure S10 for workflow). After the treatment was completed, we fixed, permeabilized, and stained the cell body, F-actin, nuclei, and pMLC with a high-content screening cell mask, phalloidin, Hoechst, and specific primary and fluorescent secondary antibodies (Figure 3E). We imaged the cells on a high-content automated microscope and processed the acquired images to isolate individual cells and further segment the cells into nucleus, cell body, and lamellipodium (Figure S11A). When we plotted the median intensity of cellular lamellipodial pMLC, we observed hysteresis (Figure S11B). In non-pretreated cells, IPA-3 increased pMLC intensity at the highest concentrations, whereas when cells were pretreated, we observed that the switch occurred at the lowest concentrations (Figure S11B). We then plotted single-cell lamellipodial pMLC levels in a histogram to observe whether IPA-3 levels altered the distribution of pMLC in the cell populations. In MDA-MB-231 without any IPA-3 treatment, pMLC was spread in a long-tailed distribution, possibly already containing two populations with distinct population averages (Figure 3F). This distribution was unaltered for the low and medium concentrations of the inhibitor, but we observed a broadening at the highest IPA-3 concentration (Figure 3F). In cells that were pretreated with IPA-3, we observed that the broadening of the pMLC distribution already occurred at low inhibitor concentrations (Figure 3G). The broadening of the distributions suggests that two, possibly three, cell populations with distinct average pMLC levels can be present under these conditions. This potentially multi-modal distribution suggests that pMLC levels may be determined by more complex signaling, which is not captured in its entirety by our model.

We then quantified cytoplasmatic F-actin in the same sample set. Analogous to what we observed with pMLC, only higher concentration of IPA-3 increased cellular F-actin (Figure S11C). In contrast, pretreating the cells increased the cellular quantity of F-actin across all IPA-3 concentrations (Figure S11C). We then plotted the F-actin single-cell distribution and observed that most non-pretreated MDA-MB-231 cells had no or low amounts of stress fibers at low or medium inhibitor concentrations. Incubation with 7.5 μM IPA-3 gave rise to a second population that was positive for F-actin (Figures 3H and 3I). Pretreatment with IPA-3 for 20 min was also sufficient to increase the presence of this second population (Figure 3J). Washing IPA-3 completely off reduced the levels of the stress fiber-containing cells but not to the levels of the initial condition, suggesting that 20 min of IPA-3 induced actin stress fibers irreversibly within the time frame of the experiment. This is possibly due to the stability of the stress fibers, and 1 hr may not be enough to efficiently disassemble them. In summary, these data showed that both pMLC and F-actin are induced by IPA-3. Instructively, we observed hysteresis for both markers of RhoA activity. Furthermore, our data showed that F-actin is bimodally distributed, and there is a likelihood that this was the case for pMLC.

### Bistable Behavior of Actin Dynamics

Given the requirement of Rac1 and RhoA interactions for actin assembly and disassembly (Burridge and Wennerberg, 2004, Nobes and Hall, 1995, Ridley and Hall, 1992) and in initiating protrusions at the leading edge (Machacek et al., 2009, Pertz et al., 2006), we speculated that IPA-3 would also regulate actin dynamics in a bistable fashion, a hypothesis we tested next.

**Figure 4:**
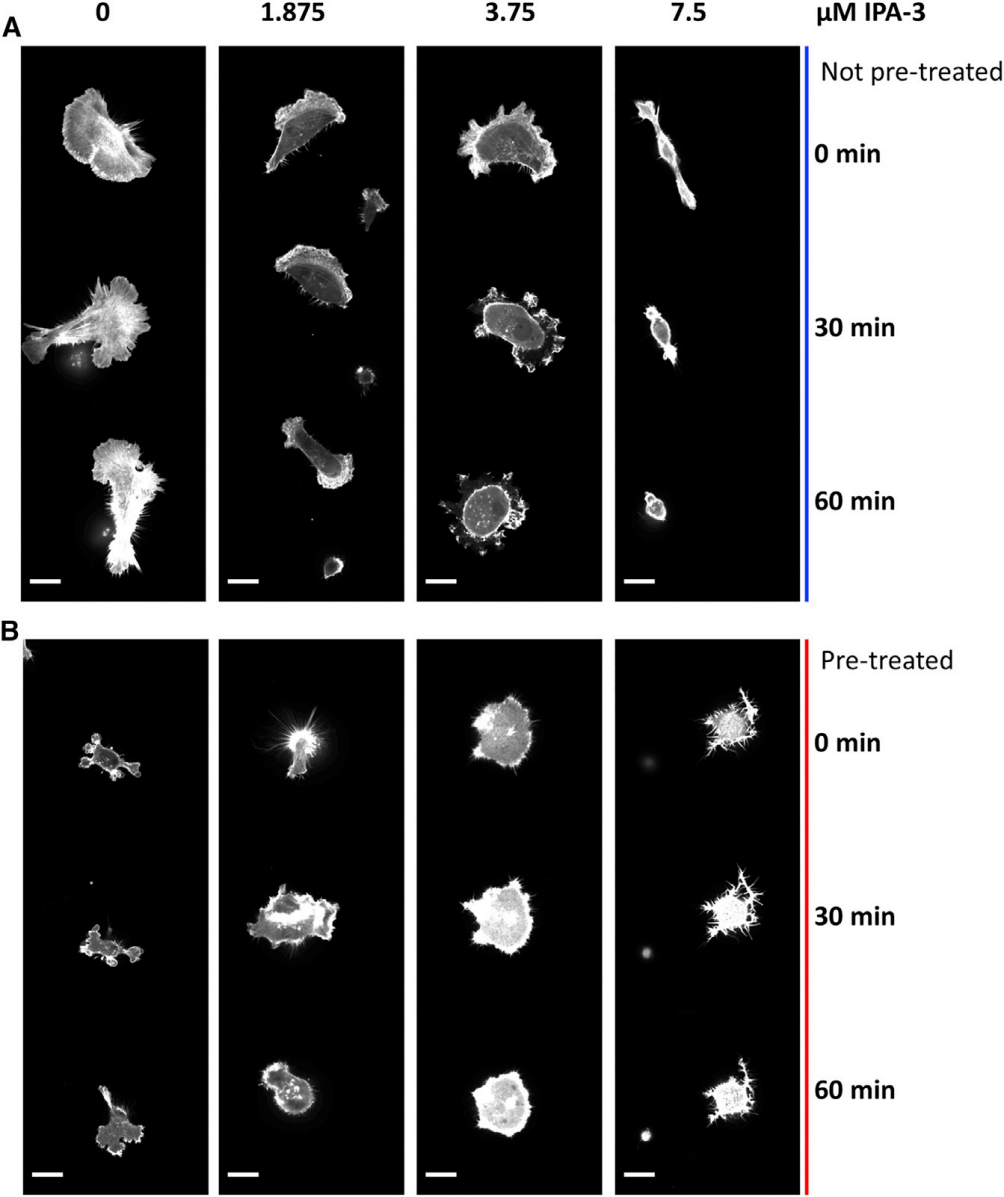
Actin Dynamics Behave in a Bistable Manner in Response to PAK Inhibition Sparsely seeded MDA-MB-231 cells expressing a LifeAct-mCherry probe were treated with the PAK inhibitor IPA-3 (A) at the indicated concentrations for 60 min (blue) or (B) by incubating cells with 7.5 μM IPA-3 for 20 min. The inhibitor was subsequently washed out, and the cells were incubated for an additional 60 min with IPA-3 at the indicated concentrations (red). Images were taken every 15 s. Montage images represent changes in actin dynamics during the cells’ migration, showing three images (0, 30, and 60 min) over the 1 hr period. Scale bar, 20 μM.

Live-cell actin polymerization dynamics can be visualized by expressing fluorescent proteins tagged to an F-actin binding peptide (LifeAct) (Riedl et al., 2008). Therefore, we generated MDA-MB-231 cells that stably express mCherry-tagged LifeAct. Similar to earlier, we exposed cells in their normal state to incrementally increasing concentrations of IPA-3. Alternatively, we first pretreated cells with high IPA-3 doses for 20 min, thereby inhibiting PAKs, and then washed out the inhibitor and again incubated cells with different IPA-3 concentrations. In both cases, we imaged the actin dynamics over 60 min (see Figure S10 for workflow). Untreated control cells exhibited a highly dynamic F-actin cytoskeleton, which rapidly pushed and retracted cellular protrusions and lamellipodia (Figure 4A; Movie S1). Low concentrations of IPA-3 (1.875 and 3.75 μM) did not affect the dynamic behavior, whereas high concentrations (7.5 μM) initially froze the actin polymerization (Figure 4A; Movies S2, S3, and S4). Subsequently, some cells contracted and membrane blebs appeared (Movie S4), which is indicative of high RhoA activity. In isolated cases, the cells even detached from the glass surface. Equally, pretreatment of the cells with high concentrations of IPA-3 resulted in stalling actin dynamics, blebbing, and a contracted phenotype (Figure 4B; Movies S5, S6, S7, and S8). When the IPA-3 concentration was dropped below 3.75 μM, actin dynamics recovered, blebbing was reduced, and the cells started spreading and initiating lamellipodia (Figure 4B; Movies S5 and S6). In contrast, cells in which the IPA-3 concentration was not altered or reduced to 3.75 μM did not recover and remained in a frozen state (Figure 4B; Movie S7). Taken together, these data show that analogous to RhoA- and Rac1-GTP, actin dynamics respond in a bistable manner when perturbed by PAK inhibition.

### Bistable Behavior of Cell Migration

Actin-driven cellular protrusions and retrograde flow are essential for efficient cell migration. It has been previously demonstrated that Rac activity is required for cell migration and that global inhibition of Rac arrests cell migration (Nobes and Hall, 1999). Therefore, based on our observation that IPA-3 affects actin dynamics and Rac1 activity levels in a bistable manner, we hypothesized that cell migration should behave analogously. To test this hypothesis, we next measured how migration of MDA-MB-231 cells was affected by PAK inhibition, performing random and directed migration assays. Random migration is the expression of the intrinsic cell directionality in the absence of any external guiding factor, whereas directed migration requires steering by an external guidance cue (Petrie et al., 2009). In both cases, the interplay and localization of Rac1 and RhoA activity determine migration speed and directionality.

**Figure 5:**
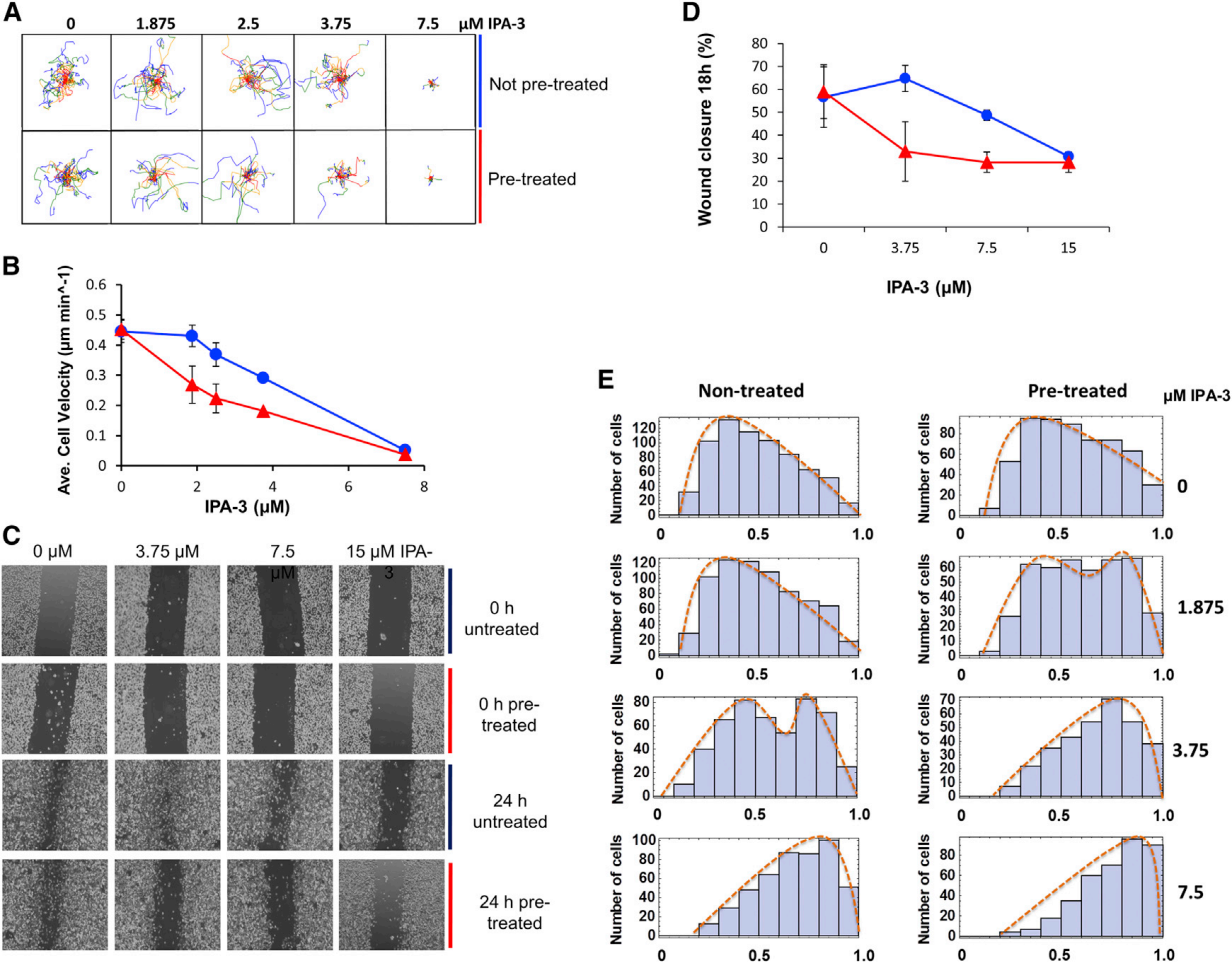
Migration of MDA-MB-231 Cells Is Regulated in a Bistable Manner by PAK (A) Undirected cell migration. Sparsely seeded MDA-MB-231 cells were treated with the PAK inhibitor IPA-3 at the indicated concentrations for 40 min (blue) or by incubating MDA-MB-231 cells with 7.5 μM IPA-3 for 20 min. The inhibitor was subsequently washed out, and the cells were incubated for an additional 20 min with IPA-3 at the indicated concentrations (red). Images were taken every 20 min, and random cell migration of 25 cells was subsequently manually tracked using ImageJ. Each line represents a single cell’s migration in one field of view over 12 hr. The line color shows the distance migrated from 0–3 hr (red), 3–6 hr (orange), 6–9 hr (green), and 9–12 hr (blue). Line origins have been artificially placed at (0,0) for display purposes, but lengths remain unchanged. The width and height of each plot is 200 μm. (B) The mean speed ± SD of three single-cell tracking biological replicates over 12 hr, a representative example of which is found in Figure 5A. The differences between pretreated and non-pretreated 1.185, 2.5, and 3.75 μM IPA-3 were all statistically significant, with p = 0.017, 0.015, and 0.0001, respectively, measured using a two-tailed, non-paired t test. Error bars represent SEM. (C) Directed cell migration. Confluent MDA-MB-231 cells were scratched with a plastic tip. IPA-3 was added at the indicated concentrations for the duration of the experiment (blue) or by incubating MDA-MB-231 cells with 15 μM IPA-3 for 20 min. The inhibitor was subsequently washed out, and the cells were incubated for the remainder of the experiment with IPA-3 at the indicated concentrations (red). Images of the wound were taken at 0 and 18 hr. (D) Graph representing the percentage of wound closure of three biological replicates. Error bars represent SD. (E) MDA-MB-231 cells were sparsely seeded on a thick collagen layer. After 24 hr, they were treated with the PAK inhibitor IPA-3 at the indicated concentrations (non-treated) or by incubating MDA-MB-231 cells with 7.5 μM IPA-3 for 20 min. The inhibitor was subsequently washed out, and the cells were incubated with IPA-3 at the indicated concentrations (pretreated). Images were taken 24 hr later. Histogram represents cell number over cell roundness, with 0 as an infinite line and 1 as a perfect circle.

To determine random migration speed, cells were seeded at low confluency and treated with incrementally increasing concentrations of IPA-3 ranging from 0 to 7.5 μM. An additional set of cells was treated with the highest concentration of IPA-3, 7.5 μM for 20 min. IPA-3 was then washed off and replaced with incrementally increasing inhibitor concentrations from 0 to 7.5 μM IPA-3, as indicated in Figure 5A. The IPA-3 concentrations are slightly different from those used in Figures 3A–3D due to batch-to-batch variability. Preliminary migration experiments done using IPA-3 from the first batch showed that full migration inhibition required 15 μM IPA-3 (Figure S12A). We took transmission-light images every 20 min for 12 hr, and the path of migration of individual cells was manually tracked (see Figure S10 for workflow). The migration paths of individual cells in a representative replicate are plotted in Figure 5A to give a visual representation of migration. The average speed of each cell was calculated and plotted against PAK inhibition (Figure S12). The quantification in Figure 5B shows that the motility of non-pretreated cells was unaffected at 2.5 μM IPA-3 but subsequently decreased in response to linearly increasing IPA-3 concentrations. Furthermore, the transition from high to low and then low to high migration speeds occurs at different threshold levels of IPA-3, indicating hysteresis. Together, these data suggest that migration of the cells is affected by PAK inhibition in a bistable manner.

Examining instantaneous cell velocities, we found that bistability persists for the duration of the experiment (Figures S12A and S12B). Bistable switches in migration velocity occur in individual cells. Consequently, the change in the population cell velocity average is caused by switch-like velocity changes in different cells rather than by gradual changes in the whole population. Therefore, we hypothesized that under conditions in which the system is bistable (1.865, 2.5, and 3.75 μM IPA-3), the velocities of cells at a given time step (instantaneous velocities) would follow a bimodal distribution, which is a hallmark of a bistable system (Birtwistle et al., 2012, Dobrzyński et al., 2014). Analysis of individual cell tracks from Figure 5A showed that this was the case (Figure S12C).

We also tested the effect of PAK inhibition on directed cell migration using a wound-healing assay. The cells were treated as before and were photographed immediately after treatment and again after 18 hr (see Figure S10 for workflow). A representative experiment is shown in Figure 5C. A quantification of three replicates is shown in Figure 5D. The results show switch-like inhibition of cell motility in response to graded PAK inhibition. In addition, the switch occurs at different thresholds depending on the initial condition of the systems, indicating the presence of hysteresis (Figures 5C and 5D). These results suggest that directed migration features a bistable response to PAK inhibition.

Finally, to show that the results produced by inhibition of PAK by IPA-3 were not due to an unspecific off-target inhibitor effect, we reproduced some of these results using an unrelated PAK inhibitor (PAK^i^). PAK^i^, a genetically encoded PAK1-3 specific inhibitor consisting of a truncated PAK1 regulatory domain, includes the kinase-binding segment that can bind to and inhibit the catalytic domain of PAK1-3 but does not have an intact CRIB domain and therefore cannot bind Rac1 directly (Volinsky et al., 2006). Expression of GFP-coupled PAK (PAK^i^) or an inactive mutant (PAK^i^-2 m) was induced by transient transfection in MDA-MB-231 cells. This alternative PAK inhibitor also inhibited Rac1 activity and migration (Figure S13), whereas the inactive mutant had no effect on either. These results confirm that these observed effects are specific to PAK inhibition.

### Bistability and Bimodality of Cell Morphology in 3D Matrices

Cells embedded in three-dimensional (3D) matrices have been shown to invade into the surrounding area by two fundamentally disparate modes of migration (Friedl and Alexander, 2011): mesenchymal and amoeboid. Mesenchymal migration requires cells to proteolytically degrade the matrix through the secretion of matrix metalloproteases (MMPs). This migration mode requires high Rac1-GTP, and it is independent of RhoA activity. Amoeboid migration, however, is independent of MMPs but requires RhoA activity. This migration type is driven by actinomyosin contractility and characterized by a rounded morphology with high RhoA activity.

MDA-MB-231 cells have predominantly elongated morphology in 3D matrices but switch to a rounded cell shape upon RhoA activation or MMP inhibition (von Thun et al., 2013). Thus, we hypothesized that RhoA-GTP elevated by IPA-3 treatment may also alter the morphology of MDA-MB-231 cells. This transition between two morphologies is driven by the RhoA activity changes. Thus, we would expect that, analogous to what we have seen at the molecular level, this switch would be bistable.

To test whether this was the case, we seeded MDA-MB-231 cells on a thick layer of collagen and allowed them to invade the collagen gel over 24 hr. We then incubated the cells either with a range of IPA-3 concentrations or with a 20 min burst of 7.5 μM IPA-3, which was replaced by a set of IPA-3 dilutions. We then imaged the cells embedded in the collagen matrix and determined their shape by automated image analysis (see Figure S10 for workflow). As expected, MDA-MB-231 cells adopted an elongated phenotype in a collagen matrix (quantified in Figure 5E). The 7.5 μM IPA-3 shifted most of the population toward a rounded phenotype. At the intermediate concentration of 3.75 μM IPA-3, we could observe that the distribution of the roundness within the cell population was bimodal. Pretreatment with IPA-3 followed by a washout shifted the switch concentration. In this case, we observed bimodality at the lower concentration of 1.875 μM IPA-3, whereas the cells were predominantly rounded at 3.75 and 7.5 μM IPA-3 (Figure 5E). Taken together, these data showed that the change in morphology is likely to be bistable, because we observed that the IPA-3 concentration needed to induce the switch depends on the initial condition. In addition, we observed bimodality, a general feature of bistable systems.

## Discussion

Rac1 and RhoA are embedded in a wider network of interactions containing extra positive feedback regulations, which were not included in our models. For example, a positive feedback between Rac1 and PAK is formed via the protein Cool-2 (cloned out of library-2) (Baird et al., 2005, Feng et al., 2002), and Rac1 can positively regulate itself in a PAK-independent manner (Tsyganov et al., 2012). However, adding these feedbacks to the system with existing double-negative feedback, or incorporating GDP dissociation inhibitors in the model, only enlarges the bistability range; it did not significantly affect the network behavior (Figure S14) (Nikonova et al., 2013).

For cells to migrate effectively, there must be a highly coordinated interplay between protrusion extension and both adhesion formation and rear end retraction driven by high Rac1 and high RhoA activities, respectively. The high activity of one GTPase requires the low activity of the other to ensure that there is no conflict in the organization of the actin cytoskeleton. The traditional view of Rac1 and RhoA activity in migrating cells was of high Rac1 activity only at the leading edge and high RhoA activity only at the trailing edge. This hypothesis of wide spatial segregation of Rac1 and RhoA activities has since been challenged by localization experiments that describe RhoA activity at the leading edge of migrating cells (Machacek et al., 2009, Pertz et al., 2006) and Rac1 activity at the tail (Gardiner et al., 2002). Though there seems to be a lack of consensus over the exact localizations of active RhoA and Rac1 during the cell movement, and we are likely only seeing a small part of a larger, more complex picture (Wang et al., 2013), a unifying motif is that the activation zones of Rac1 and RhoA are mutually exclusive, either spatially or temporally (Guilluy et al., 2011, Pertz, 2010). This exclusivity found in the spatial and temporal localization of Rac1 and RhoA has been proposed to arise from the bistability of the system (Jilkine et al., 2007, Semplice et al., 2012). Bistability allows the cell (or area of a cell) to make a discrete digital decision in the face of a range of conflicting external signal gradients and ensures that the decision made by the cell is fairly robust. In that way, the cell is responsive to its surroundings but not so sensitive that it is unable to make efficient progress.

Due to the hysteresis present in the system, once PAK is inhibited, it remains locked in that state even at lower inhibitor concentrations. Such circuitries with bistable and hysteretic response characteristics could make attractive targets for therapy approaches using dose variation, because an initial high dose would switch the system to the inhibited state, which can be maintained using lower drug concentrations. This behavior is desirable for minimizing drug toxicity and in terms of pharmacokinetics ensuring a long-lasting inhibition even when the drug concentrations decline. Our cell motility experiments show that undirected and directed migration are arrested in two dimensions by strong inhibition of PAK (e.g., Figure 4A). This indicates that PAK could be an attractive drug target for blocking the migration of cancer cells. Although IPA-3 is not suitable for clinical use (Zhao and Manser, 2010), our results are valid for various mechanisms of PAK inhibition, and this may stimulate the development of therapeutically appropriate PAK inhibitors in the future.

## Experimental Procedures

For a detailed description of the models and model analyses, see Supplemental Information.

### Cell Culture and Reagents

MDA-MB-231 cells were cultured using standard techniques. The plasmids, reagents, and antibodies used in this study are listed in Supplemental Information.

### Liquid Chromatography-Tandem Mass Spectrometry, Protein Identification and Quantitation, and IPA-3 Quantitation

Samples were analyzed on a Q Exactive mass spectrometer (Thermo Scientific) coupled to ultra-high-performance liquid chromatography as described (Farrell et al., 2014). Identification and quantitation was performed using the MaxQuant/Perseus software suite for proteins or Xcalibur for IPA-3.

### Immunofluorescence

Standard protocols were followed. Images were acquired in an unsupervised manner on an ImageXpress Micro wide-field microscope and were analyzed using the MetaXpress Custom Module Editor (Molecular Devices).

### Live-Cell Imaging

Standard methods were employed. MDA-MB-231 or a clone stably expressing LifeAct-mCherry was seeded in an optical-bottomed plate. Images were acquired on a spinning-disc laser confocal Nikon microscope, an Incucyte ZOOM system, or a Zeiss Axiovert 200M.

### Mathematical Modeling

Mathematical modeling was implemented in Mathematica v.8.0.1.0 (Wolfram Research, 2010). Stability analysis was carried out using XPPaut and Mathematica. Multi-dimensional dynamic analysis and visualization were conducted using DYVIPAC (Nguyen et al., 2015). Bimodal data analysis and plotting was conducted using the R package ggplot2 (Wickham, 2009).

## Author Contributions

Design of Project, A.v.K., L.K.N., M.R.B., and B.N.K.; Biochemical and Cell Biological Assays, A.v.K., K.M.B., N.V., K.K., N.M., J.C.D., J.-C.B.-W., A.J.F., and N.O.C.; Model Development and Analysis, K.M.B., L.K.N., B.N.K., M.A.T., M.D., A.K., and A.D.; Manuscript Writing, W.K., K.M.B., A.v.K., L.K.N., and B.N.K. A.v.K., L.K.N., and B.N.K. contributed equally to this work.

## Supplemental Information

Document S1. Supplemental Experimental Procedures, Figures S1–S14, and Tables S1–S5

Table S6. Copy Numbers as Determined by the Proteome Ruler Approach across the Identified Proteome, Related to Figure 2

Movie S1. Time Lapse of Actin Dynamics of Untreated Cells, Related to Figure 4

Movie S2. Time Lapse of Actin Dynamics Cells Treated with 1.875 μM IPA-3, Related to Figure 4

Movie S3. Time Lapse of Actin Dynamics Cells Treated with 3.75 μM IPA-3, Related to Figure 4

Movie S4. Time Lapse of Actin Dynamics Cells Treated with 7.5 μM IPA-3, Related to Figure 4

Movie S5. Time Lapse of Actin Dynamics Cells Pre-treated with 7.5 μM and Then 0 μM IPA-3, Related to Figure 4

Movie S6. Time Lapse of Actin Dynamics Cells Pre-treated with 7.5 μM and Then 1.875 μM IPA-3, Related to Figure 4

Movie S7. Time Lapse of Actin Dynamics Cells Pre-treated with 7.5 μM and Then 3.75 μM IPA-3, Related to Figure 4

Movie S8. Time Lapse of Actin Dynamics Cells Pre-treated with 7.5 μM and Then 7.5 μM IPA-3, Related to Figure 4

Document S2. Article plus Supplemental Information
