# Supplementary material for: Bistability in the Rac1, PAK, and RhoA Signaling Network Drives Actin Cytoskeleton Dynamics and Cell Motility Switches
Source: Cell Syst. 2016 Jan 27;2(1):38–48. doi: 10.1016/j.cels.2016.01.003 (PMC4802415; doi:10.1016/j.cels.2016.01.003)
Supplement: Document S1. Supplemental Experimental Procedures, Figures S1–S14, and Tables S1–S5 [file mmc1.pdf]

Cell Systems

Supplemental Information

Bistability in the Rac1, PAK, and RhoA Signaling Network Drives Actin Cytoskeleton Dynamics and Cell Motility Switches

Kate M. Byrne, Naser Monsefi, John C. Dawson, Andrea Degasperi, Jimi-Carlo Bukowski-Wills, Natalia Volinsky, Maciej Dobrzyński, Marc R. Birtwistle, Mikhail A. Tsyganov, Anatoly Kiyatkin, Katarzyna Kida, Andrew J. Finch, Neil O. Carragher, Walter Kolch, Lan K. Nguyen, Alex von Kriegsheim, and Boris N. Kholodenko

Content:

Supplemental Figures and Files

Fig. S1. Related to Figure 1. Additional experimental data
Fig. S2. Related to Figure 2. Illustration of the multi-dimensional dynamic analysis and visualization using Parallel Coordinates.
Fig. S3. Related to Figure 2. Multi-parametric dynamical analyses for 5 model state variables: Rac1, RhoA, PAK, GEF-H1 and protein 14-3-3 totals.
Fig. S4. Related to Figure 2. Comparative multi-parametric dynamical analyses for 5 model state variables: Rac1, RhoA, PAK, GEF-H1 and protein 14-3-3 totals.
Fig. S5. Related to Figure 2. Effect of kinetic parameters' variation on the bistable region in the 5D protein abundance space (identified in Fig. 2c).
Fig. S6. Related to Figure 2. Multi-dimensional bistability analysis of kinetic parameter variations when the protein abundances in the model are fixed at the values determined in MDA-MB-231 cells.
Fig. S7. Related to Figure 2. Multi-parametric dynamical analyses for 5 model state variables in the dimensionless model.
Fig. S8. Related to Figure 2. Dependence of bistability on PAK inhibition and the dose-response curves on parameter variations
Fig. S9. Related to Figure 3. Quantitation of inter-cellular IPA-3 in MDA-MB-231 cells after a drug washout
Fig. S10. Related to Figure 3, 4, 5. Workflow diagram of experimental validations
Fig. S11. Related to Figure 3. Hysteresis of pMLC and F-Actin in response to PAK inhibition.
Figure S12. Related to Figure 5. Bistability and bimodality is present in instantaneous cell velocities.
Figure S13. Related to Figure 5. PAK inhibition negatively regulates cell migration and Rac1 activity.
Figure S14. Related to Figure 2. Effects of additional positive feedback loops on the bistable behaviour

Movies S1. Related to Figure 4. Time laps of actin dynamics of untreated cells
Movies S2. Related to Figure 4. Time laps of actin dynamics cells treated with 1.875 μM IPA-3
Movies S3. Related to Figure 4. Time laps of actin dynamics cells treated with 3.75 μM IPA-3
Movies S4. Related to Figure 4. Time laps of actin dynamics cells treated with 7.5 μM IPA-3
Movies S5. Related to Figure 4. Time laps of actin dynamics cells pre-treated with 7.5 and then 0 μM IPA-3
Movies S6. Related to Figure 4. Time laps of actin dynamics cells pre-treated with 7.5 and then 1.875 μM IPA-3
Movies S7. Related to Figure 4. Time laps of actin dynamics cells pre-treated with 7.5 and then 3.75 μM IPA-3
Movies S8. Related to Figure 4. Time laps of actin dynamics cells pre-treated with 7.5 and then 7.5 μM IPA-3

Supplemental Figure Legends

Supplemental Tables

Table S1. Related to Figure 1. Reactions and reaction rates for mechanistic model.
Table S2. Related to Figure 1. Ordinary differential equations of mechanistic model.
Table S3. Related to Figure 1. Protein totals for the mechanistic model.
Table S4 .Related to Figure 1. Parameter values used in the mechanistic model.
Table S5. Related to Figure 2. Protein copy numbers and concentrations**.**
Table S6. Related to Figure 2. Copy numbers as determined by the proteome ruler approach across the identified proteome.

Supplemental Experimental Procedures.

Mathematical Models Development, Analysis and Supplemental Methods

S1. Construction of a kinetic model for the integrated Rac1-PAK-RhoA pathway.
S1.1. Model description and assumptions
S1.1.1. Activation of PAK by Rac1
S1.1.2. PAK inhibition of RhoA through inhibition of RhoGEF
S1.1.3. Potential pathways of Rac1 inhibition by RhoA through Rac1 GAPs
S1.1.4. Inhibition of PAK by a chemical inhibitor IPA-3
S1.2. Model reaction rates and equations

S1.3. Selection of model parameter values

S2. Model dynamical analysis
S2.1. Dynamical assessment based on linear stability analysis
S2.2. Bifurcation diagrams in low dimension (2D)
S2.3. Multi-dimensional analysis of model dynamics to probe the parameter space

S3. Dimensionless model of the Rac1-PAK-RhoA system
S3.1. Transformation to the dimensionless model
S3.2. Bistability analysis of the dimensionless model using DYVIPAC

S4. Modelling the effects of additional positive feedbacks
S4.1. Simplified model of the Rac1-PAK-RhoA system
S4.2. Examining the effect of added positive feedbacks

S5. Supplemental Materials and Methods.

Supplemental References

Figure S1

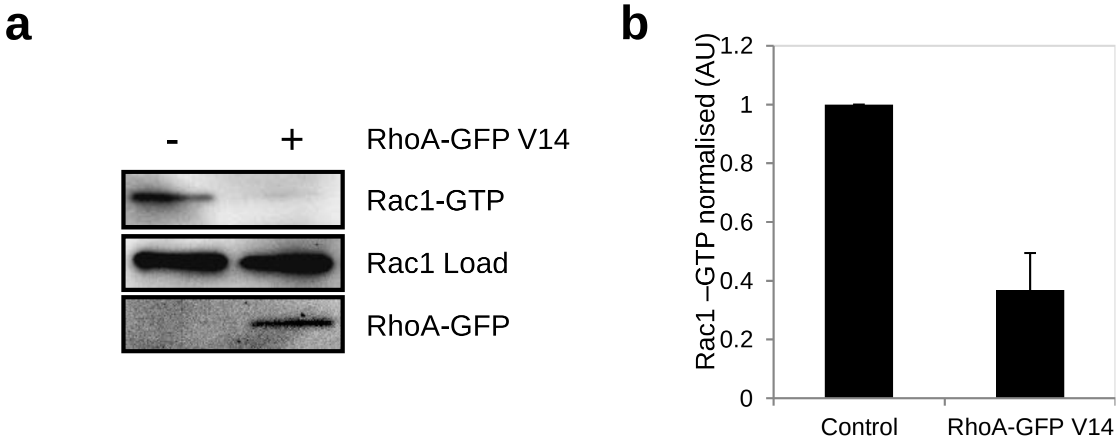
a
-
+
RhoA-GFP V14
Rac1-GTP
Rac1 Load
RhoA-GFP
b
Rac1 –GTP normalised (AU)
1.2
1
0.8
0.6
0.4
0.2
0
Control
RhoA-GFP V14

Figure S2

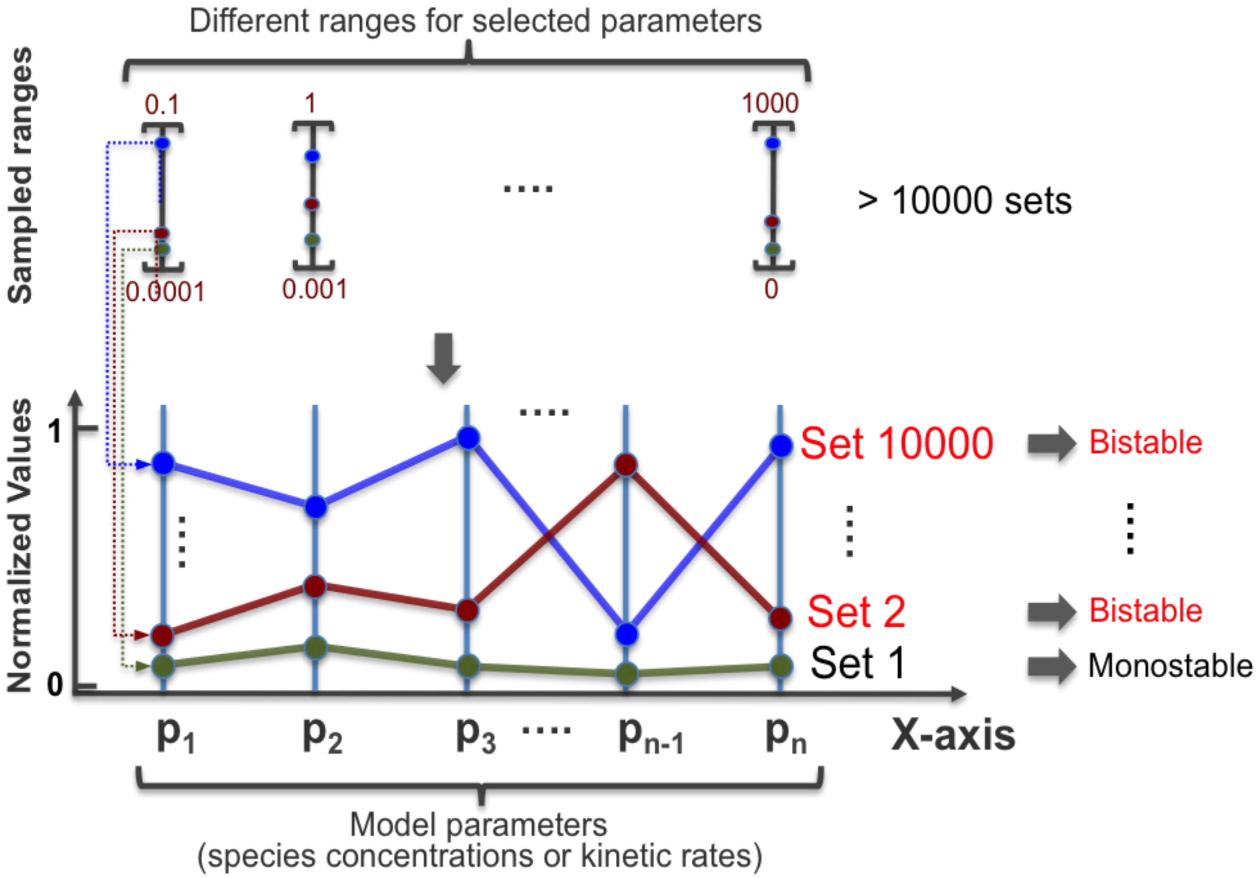

Different ranges for selected parameters
Sampled ranges
0.1
0.0001
1
0.001
1000
0
....
> 10000 sets
Normalized Values
1
0
....
Set 10000
Set 2
Set 1
Bistable
Bistable
Monostable
p1
p2
p3
....
pn-1
pn
X-axis
Model parameters
(species concentrations or kinetic rates)

Figure S3

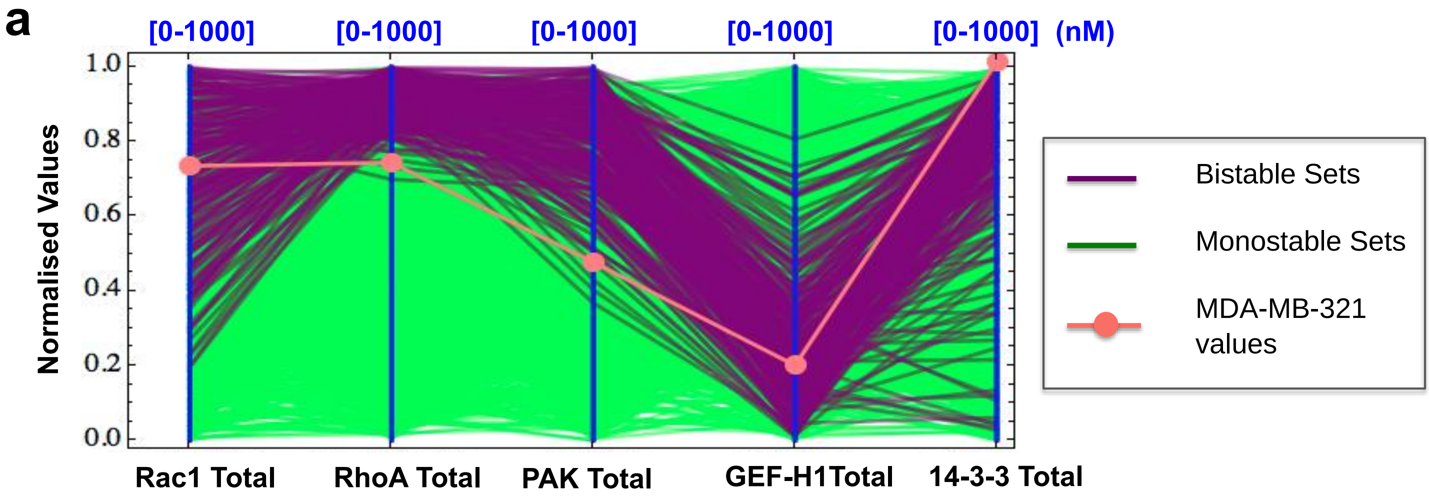
a
[0-1000]
[0-1000]
[0-1000]
[0-1000]
[0-1000] (nM)
1.0
0.8
0.6
0.4
0.2
0.0
Normalised Values
Rac1 Total
RhoA Total
PAK Total
GEF-H1Total
14-3-3 Total
Bistable Sets
Monostable Sets
MDA-MB-321 values

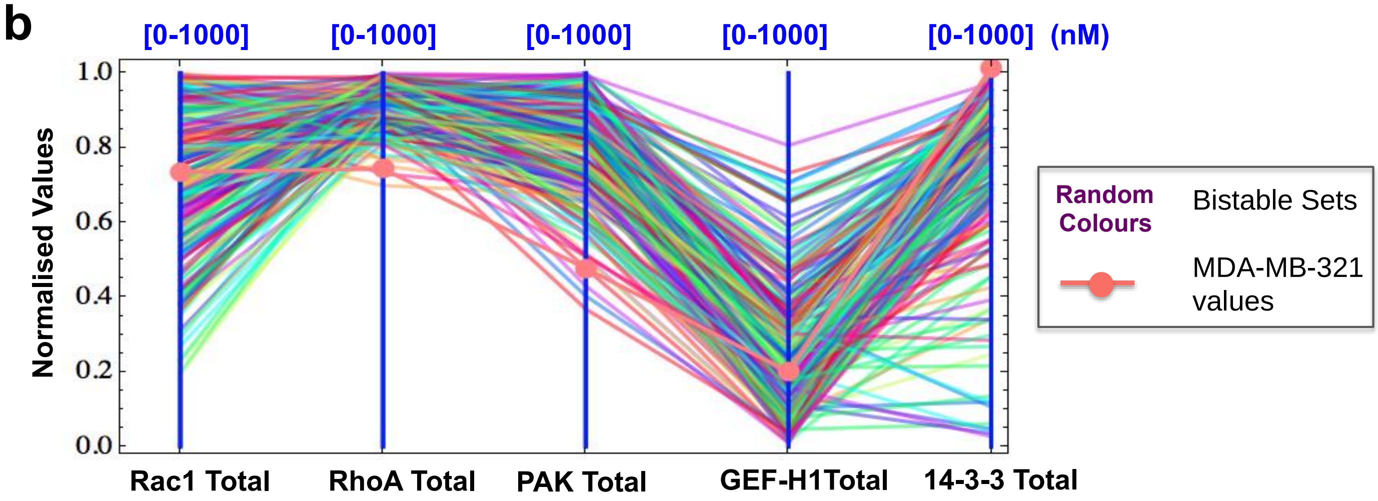
b
[0-1000]
[0-1000]
[0-1000]
[0-1000]
[0-1000] (nM)
1.0
0.8
0.6
0.4
0.2
0.0
Normalised Values
Rac1 Total
RhoA Total
PAK Total
GEF-H1Total
14-3-3 Total
Random Colours
Bistable Sets
MDA-MB-321 values

Figure S4

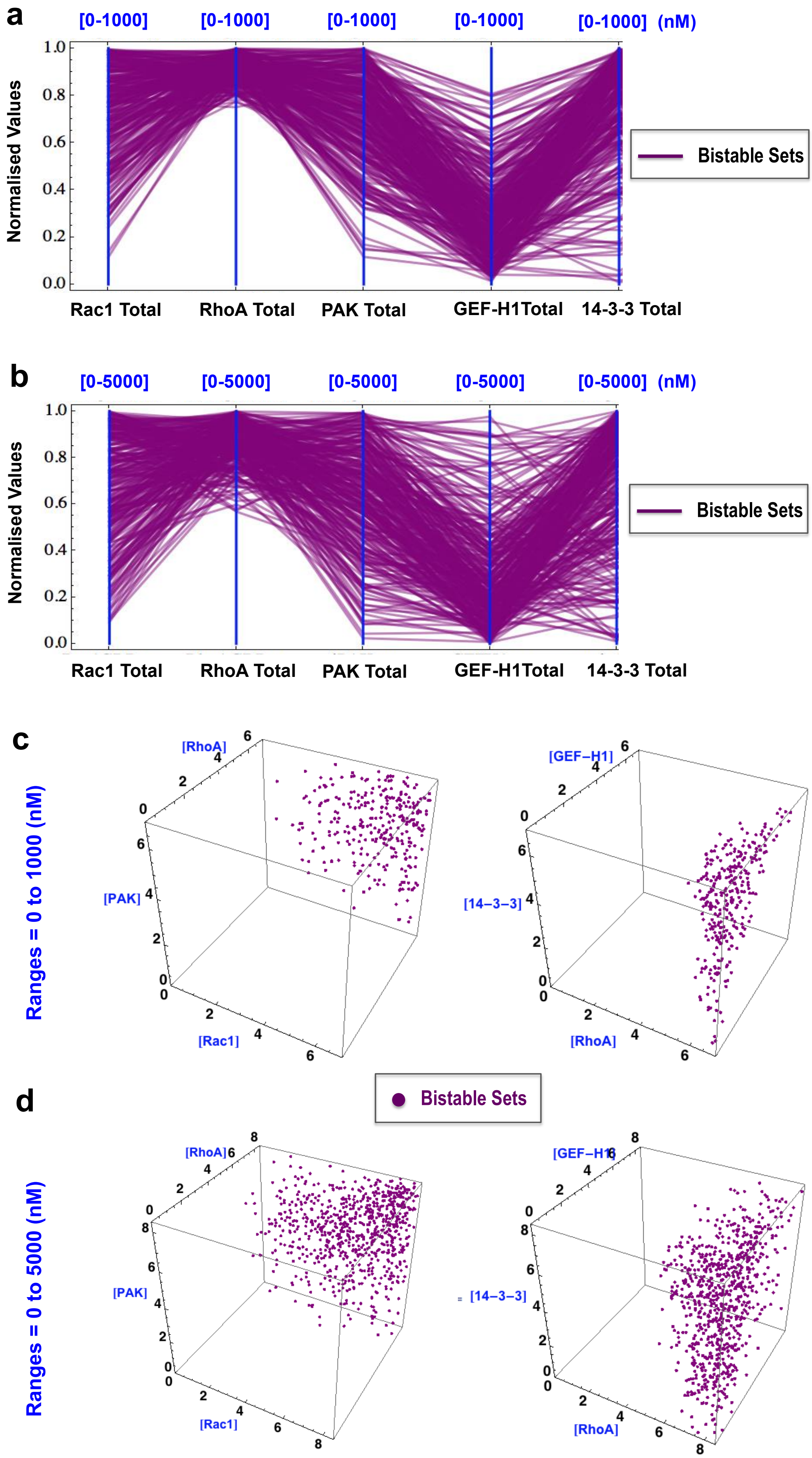

a
[0-1000]
[0-1000]
[0-1000]
[0-1000]
[0-1000] (nM)
Normalised Values
1.0
0.8
0.6
0.4
0.2
0.0
Bistable Sets
Rac1 Total
RhoA Total
PAK Total
GEF-H1Total
14-3-3 Total
b
[0-5000]
[0-5000]
[0-5000]
[0-5000]
[0-5000] (nM)
Normalised Values
Bistable Sets
Rac1 Total
RhoA Total
PAK Total
GEF-H1Total
14-3-3 Total
c
Ranges = 0 to 1000 (nM)
[RhoA]
[PAK]
[Rac1]
[GEF–H1]
[14–3–3]
[RhoA]
d
Bistable Sets
Ranges = 0 to 5000 (nM)
[RhoA]
[PAK]
[Rac1]
[GEF–H1]
[14–3–3]
[RhoA]

Figure S5

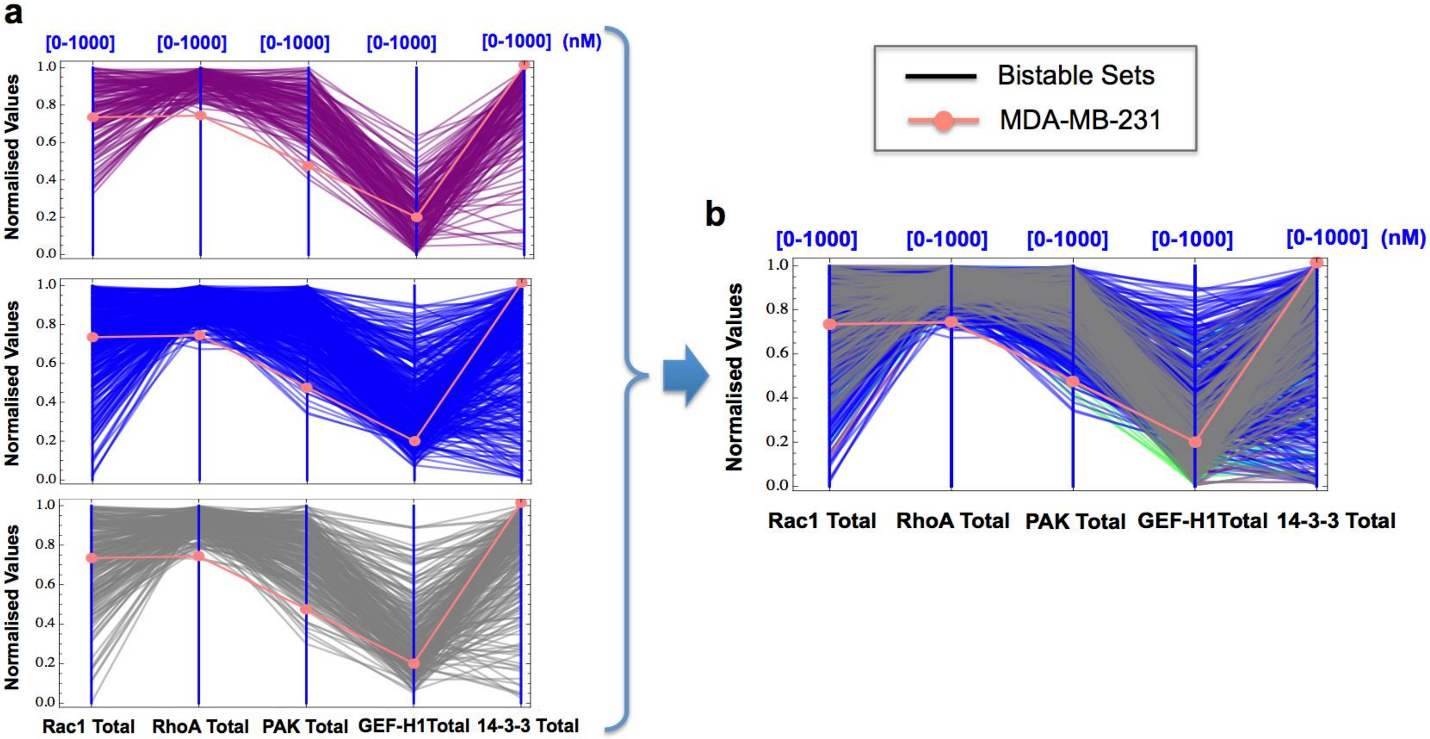
a
[0-1000]
[0-1000]
[0-1000]
[0-1000]
[0-1000] (nM)
Normalised Values
1.0
0.8
0.6
0.4
0.2
0.0
Rac1 Total
RhoA Total
PAK Total
GEF-H1Total
14-3-3 Total
Bistable Sets
MDA-MB-231
b
[0-1000]
[0-1000]
[0-1000]
[0-1000]
[0-1000] (nM)
Normalised Values
Rac1 Total
RhoA Total
PAK Total
GEF-H1Total
14-3-3 Total

Figure S5

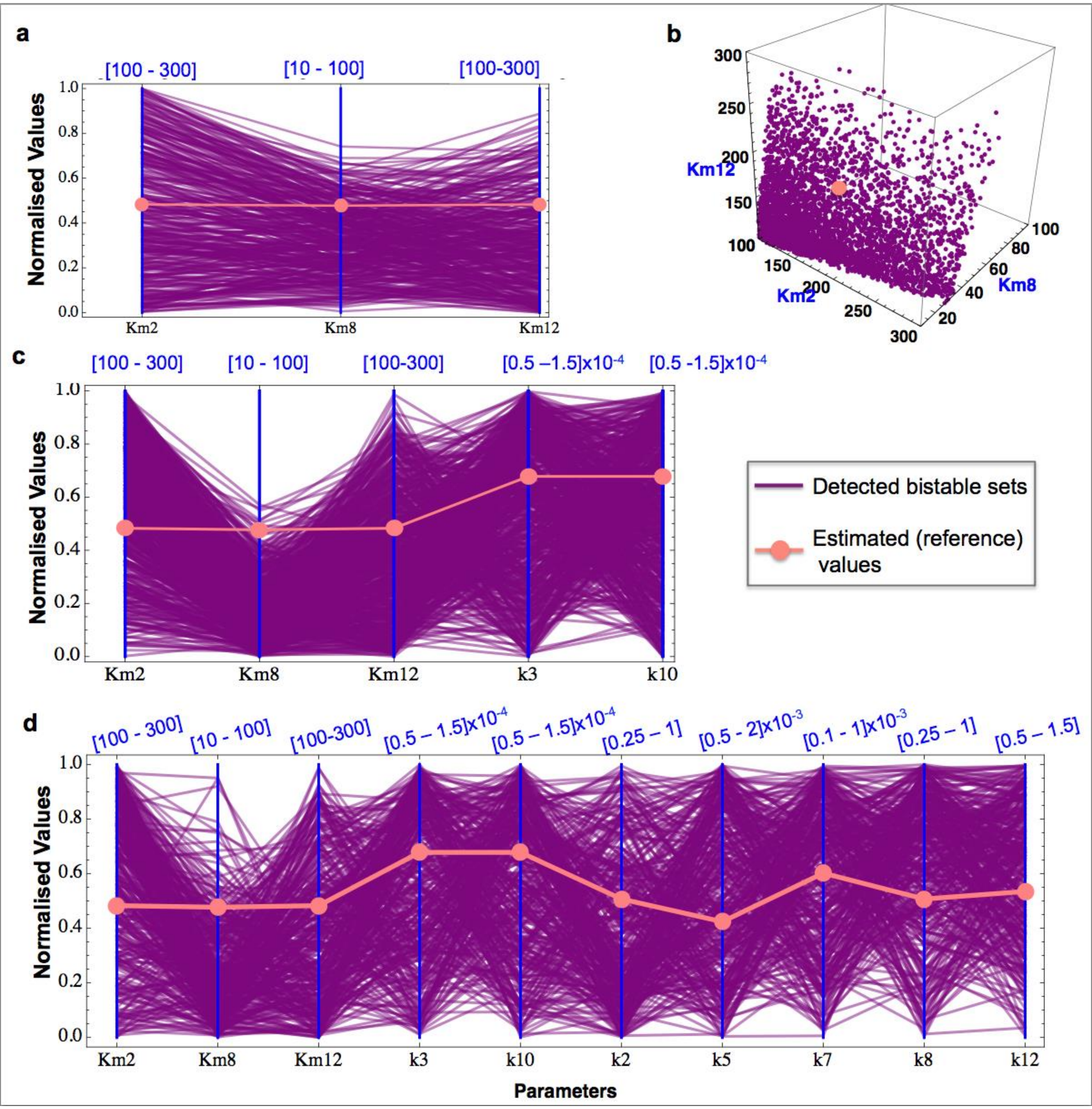

a
[100 - 300]
[10 - 100]
[100-300]
Normalised Values
1.0
0.8
0.6
0.4
0.2
0.0
Km2
Km8
Km12
b
300
250
200
150
100
Km12
150
200
250
300
Km2
100
80
60
40
20
Km8
c
[100 - 300]
[10 - 100]
[100-300]
[0.5 –1.5]x10-4
[0.5 -1.5]x10-4
Normalised Values
1.0
0.8
0.6
0.4
0.2
0.0
Km2
Km8
Km12
k3
k10
Detected bistable sets
Estimated (reference) values
d
[100 - 300]
[10 - 100]
[100-300]
[0.5 – 1.5]x10-4
[0.5 – 1.5]x10-4
[0.25 – 1]
[0.5 - 2]x10-3
[0.1 - 1]x10-3
[0.25 – 1]
[0.5 – 1.5]
Normalised Values
1.0
0.8
0.6
0.4
0.2
0.0
Km2
Km8
Km12
k3
k10
k2
k5
k7
k8
k12
Parameters

Figure S7

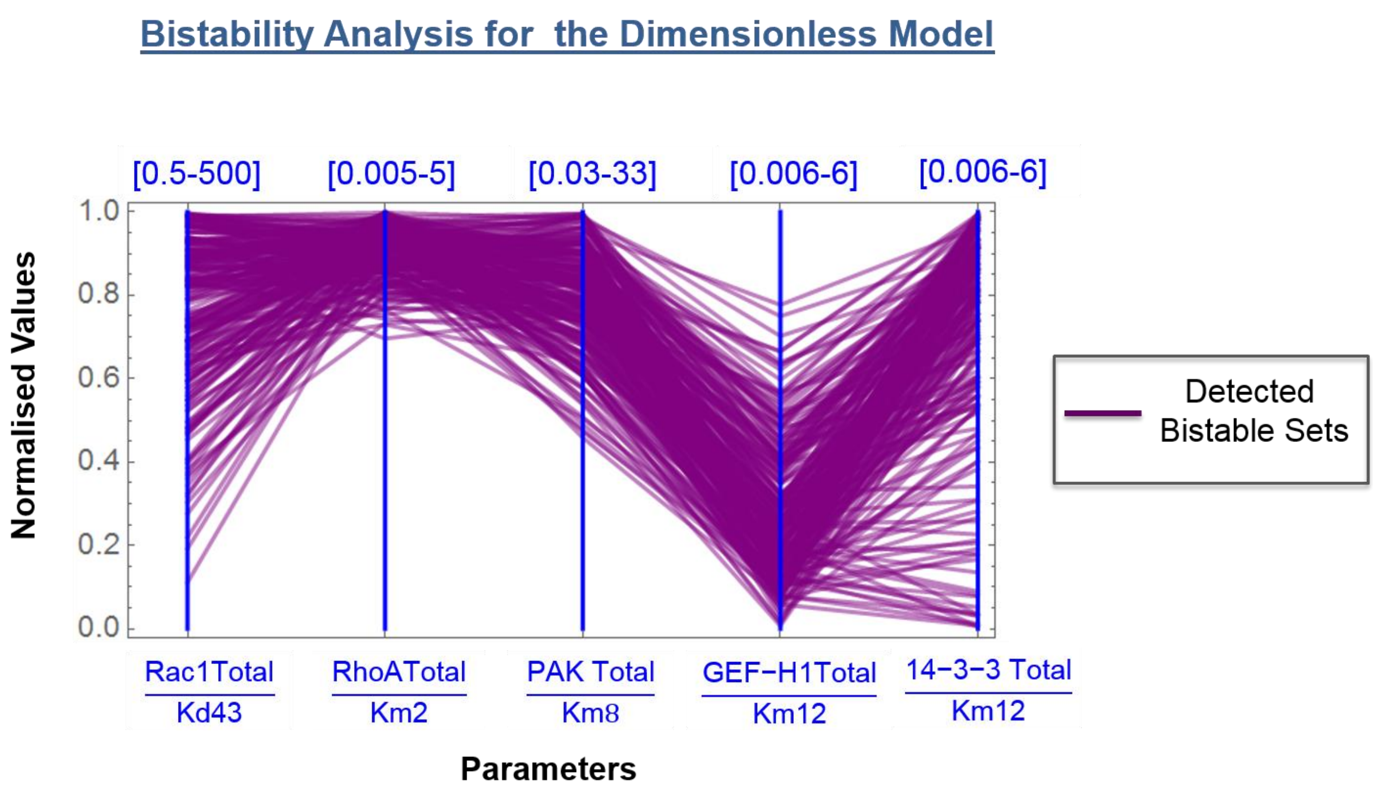

Bistability Analysis for the Dimensionless Model
[0.5-500]
[0.005-5]
[0.03-33]
[0.006-6]
[0.006-6]
1.0
0.8
0.6
0.4
0.2
0.0
Normalised Values
Rac1Total / Kd43
RhoATotal / Km2
PAK Total / Km8
GEF−H1Total / Km12
14−3−3 Total / Km12
Parameters
Detected Bistable Sets

Figure S8

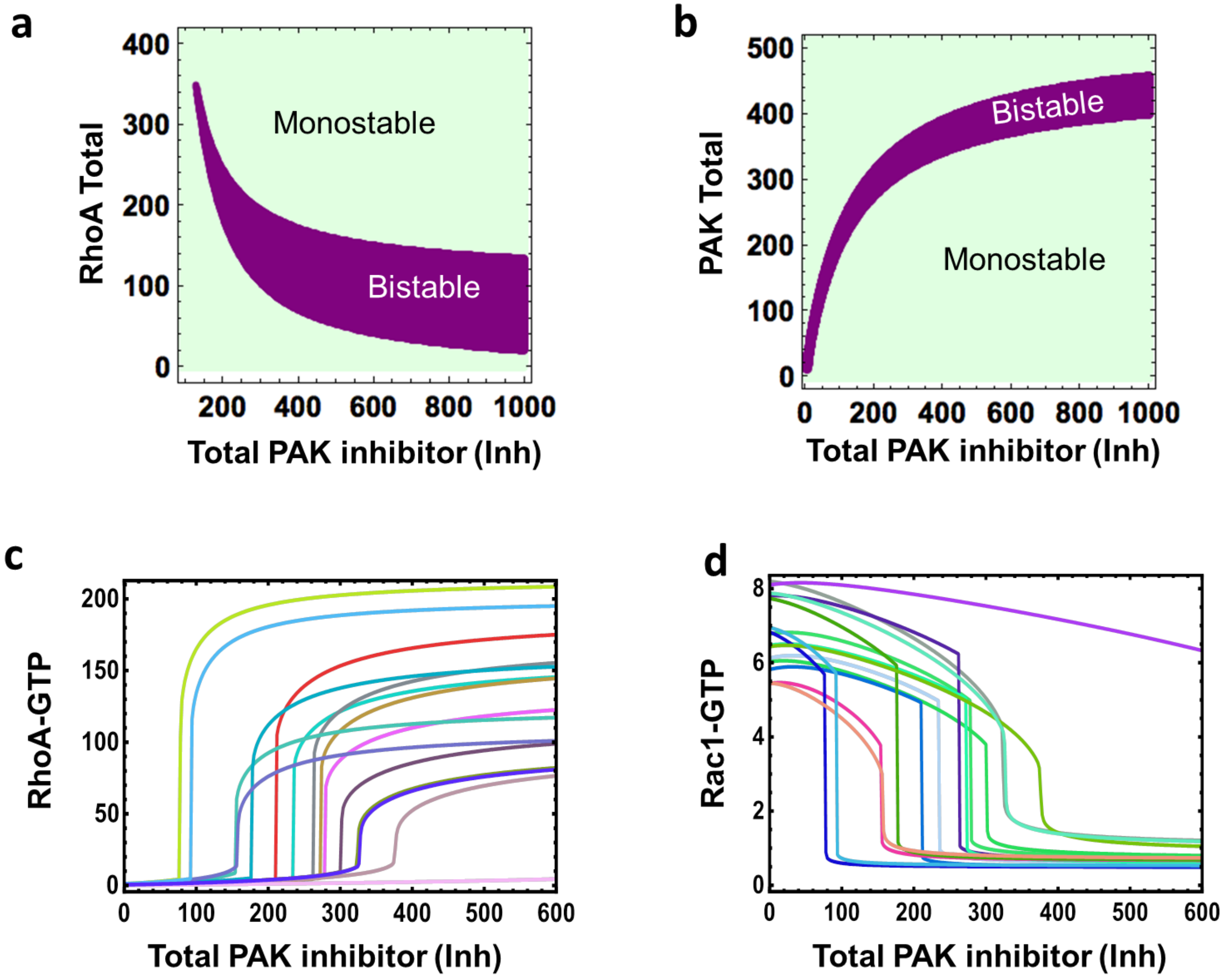
a
RhoA Total
400
300
200
100
0
Monostable
Bistable
200
400
600
800
1000
Total PAK inhibitor (Inh)
b
PAK Total
500
400
300
200
100
0
Bistable
Monostable
0
200
400
600
800
1000
Total PAK inhibitor (Inh)
c
RhoA-GTP
200
150
100
50
0
0
100
200
300
400
500
600
Total PAK inhibitor (Inh)
d
Rac1-GTP
8
6
4
2
0
0
100
200
300
400
500
600
Total PAK inhibitor (Inh)

Figure S9

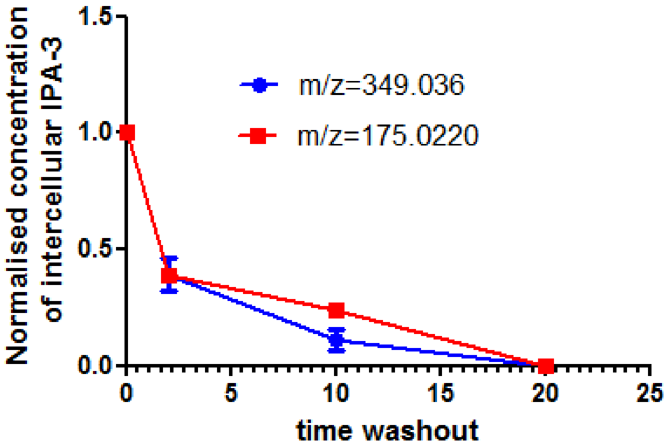

Normalised concentration of intercellular IPA-3
1.5
1.0
0.5
0.0
m/z=349.036
m/z=175.0220
0
5
10
15
20
25
time washout

Figure S10

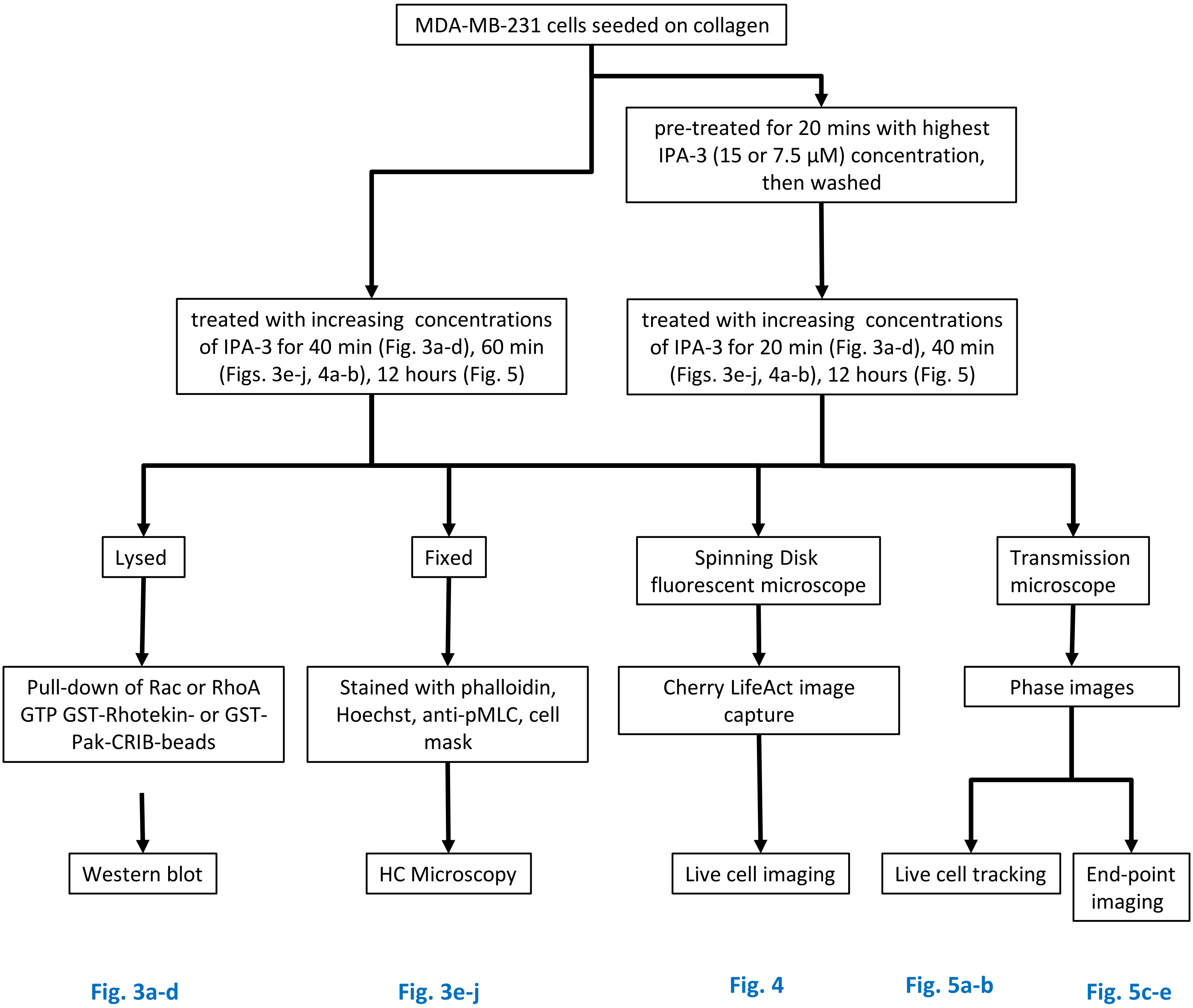

MDA-MB-231 cells seeded on collagen
pre-treated for 20 mins with highest IPA-3 (15 or 7.5 µM) concentration, then washed
treated with increasing concentrations of IPA-3 for 40 min (Fig. 3a-d), 60 min (Figs. 3e-j, 4a-b), 12 hours (Fig. 5)
treated with increasing concentrations of IPA-3 for 20 min (Fig. 3a-d), 40 min (Figs. 3e-j, 4a-b), 12 hours (Fig. 5)
Lysed
Fixed
Spinning Disk fluorescent microscope
Transmission microscope
Pull-down of Rac or RhoA GTP GST-Rhotekin- or GST-Pak-CRIB-beads
Stained with phalloidin, Hoechst, anti-pMLC, cell mask
Cherry LifeAct image capture
Phase images
Western blot
HC Microscopy
Live cell imaging
Live cell tracking
End-point imaging
Fig. 3a-d
Fig. 3e-j
Fig. 4
Fig. 5a-b
Fig. 5c-e

Figure S11

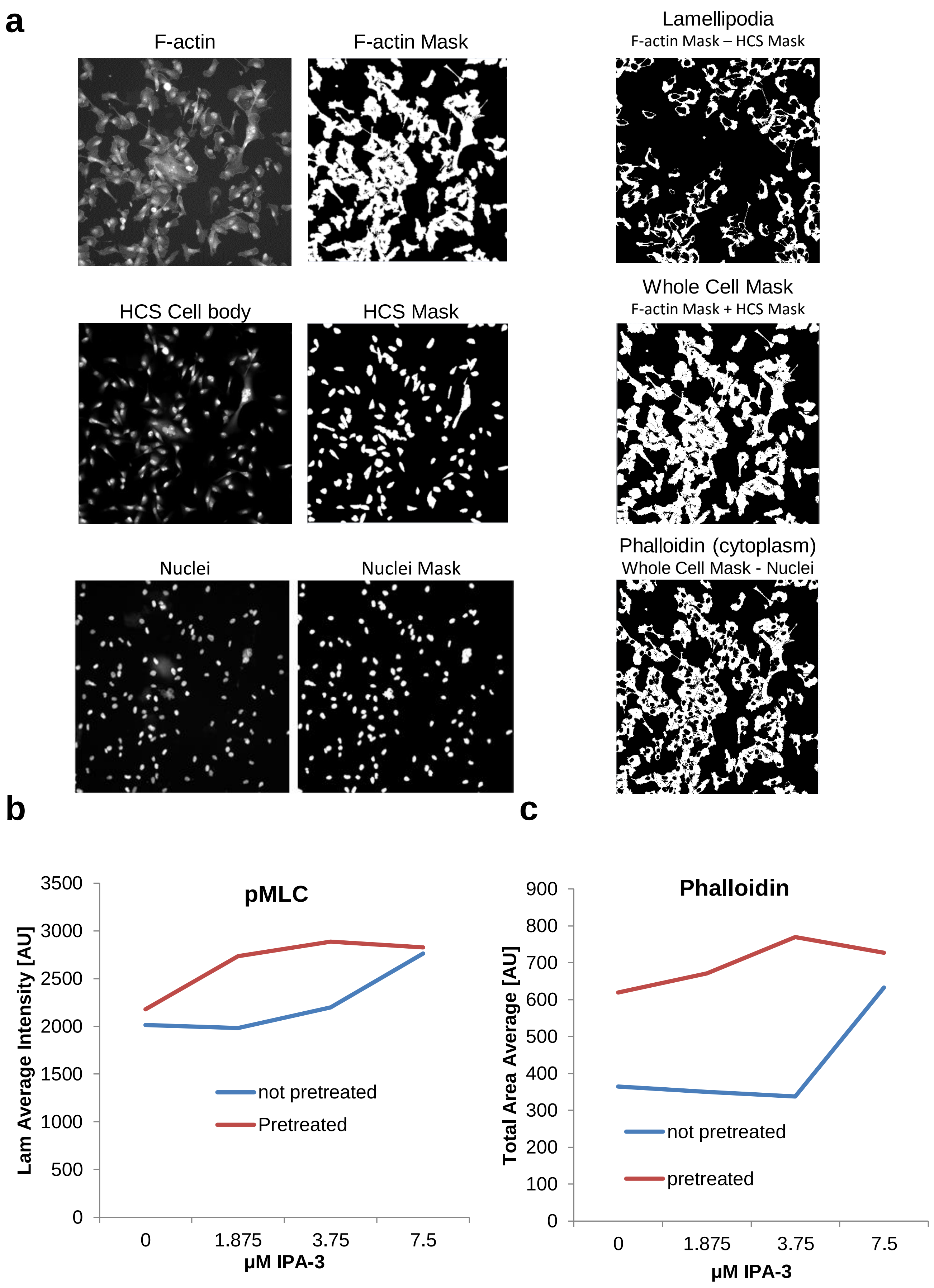

a
F-actin
F-actin Mask
Lamellipodia
F-actin Mask – HCS Mask
HCS Cell body
HCS Mask
Whole Cell Mask
F-actin Mask + HCS Mask
Nuclei
Nuclei Mask
Phalloidin (cytoplasm)
Whole Cell Mask - Nuclei
b
pMLC
Lam Average Intensity [AU]
3500
3000
2500
2000
1500
1000
500
0
not pretreated
Pretreated
0
1.875
3.75
7.5
µM IPA-3
c
Phalloidin
Total Area Average [AU]
900
800
700
600
500
400
300
200
100
0
not pretreated
pretreated
0
1.875
3.75
7.5
µM IPA-3

Figure S12

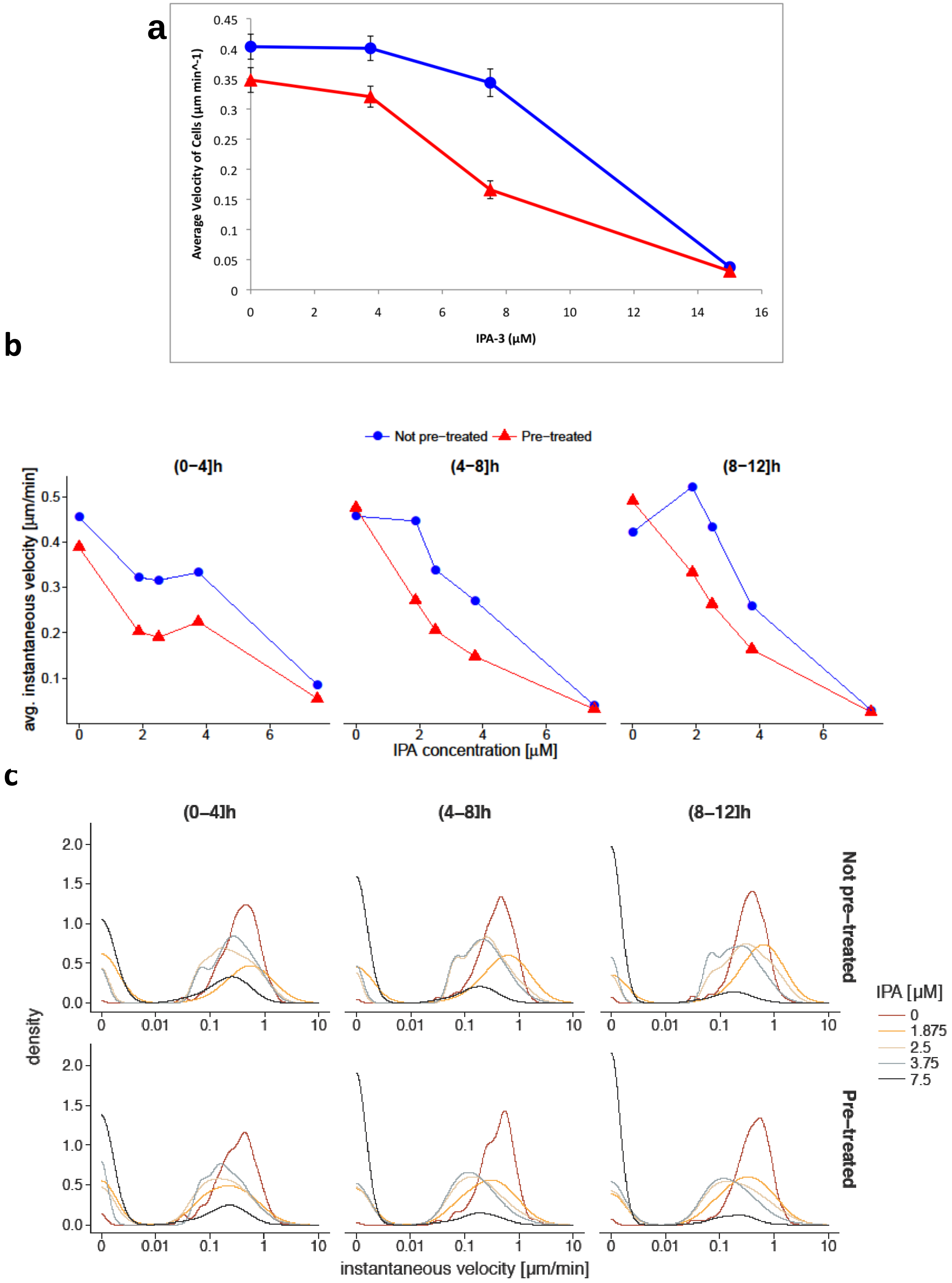

a
Average Velocity of Cells (µm min^-1)
0.45
0.4
0.35
0.3
0.25
0.2
0.15
0.1
0.05
0
0
2
4
6
8
10
12
14
16
IPA-3 (µM)
b
Not pre−treated
Pre−treated
(0−4]h
(4−8]h
(8−12]h
avg. instantaneous velocity [µm/min]
0.5
0.4
0.3
0.2
0.1
IPA concentration [µM]
c
(0−4]h
(4−8]h
(8−12]h
density
2.0
1.5
1.0
0.5
0.0
0
0.01
0.1
1
10
Not pre−treated
Pre−treated
IPA [µM]
0
1.875
2.5
3.75
7.5
instantaneous velocity [µm/min]

Figure S13

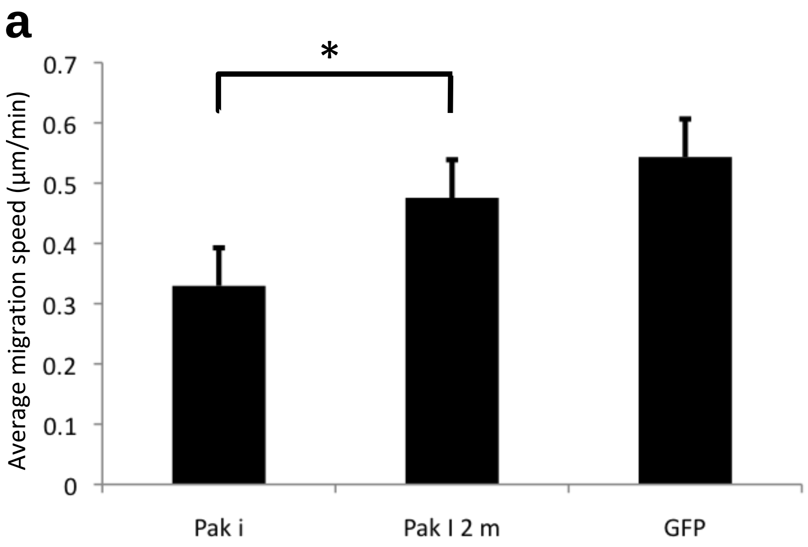

a
*
Average migration speed (µm/min)
0.7
0.6
0.5
0.4
0.3
0.2
0.1
0
Pak i
Pak I 2 m
GFP

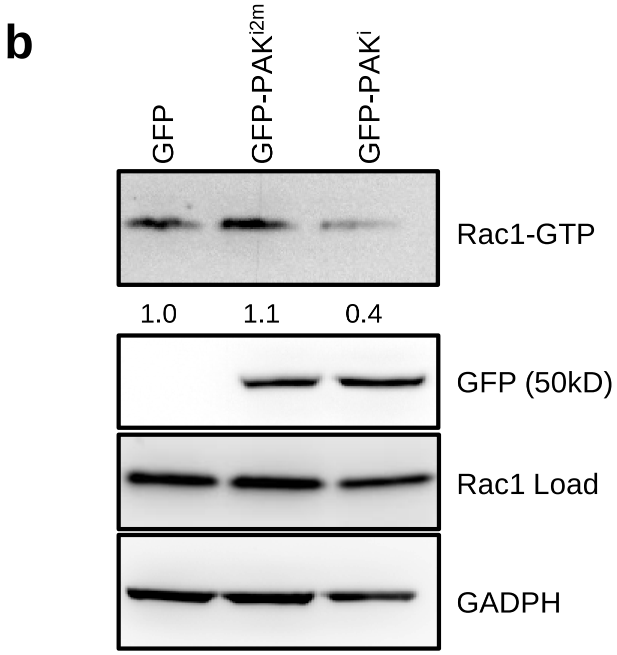

b
GFP
GFP-PAK^{i2m}
GFP-PAK^{i}
Rac1-GTP
1.0
1.1
0.4
GFP (50kD)
Rac1 Load
GADPH

Figure S14

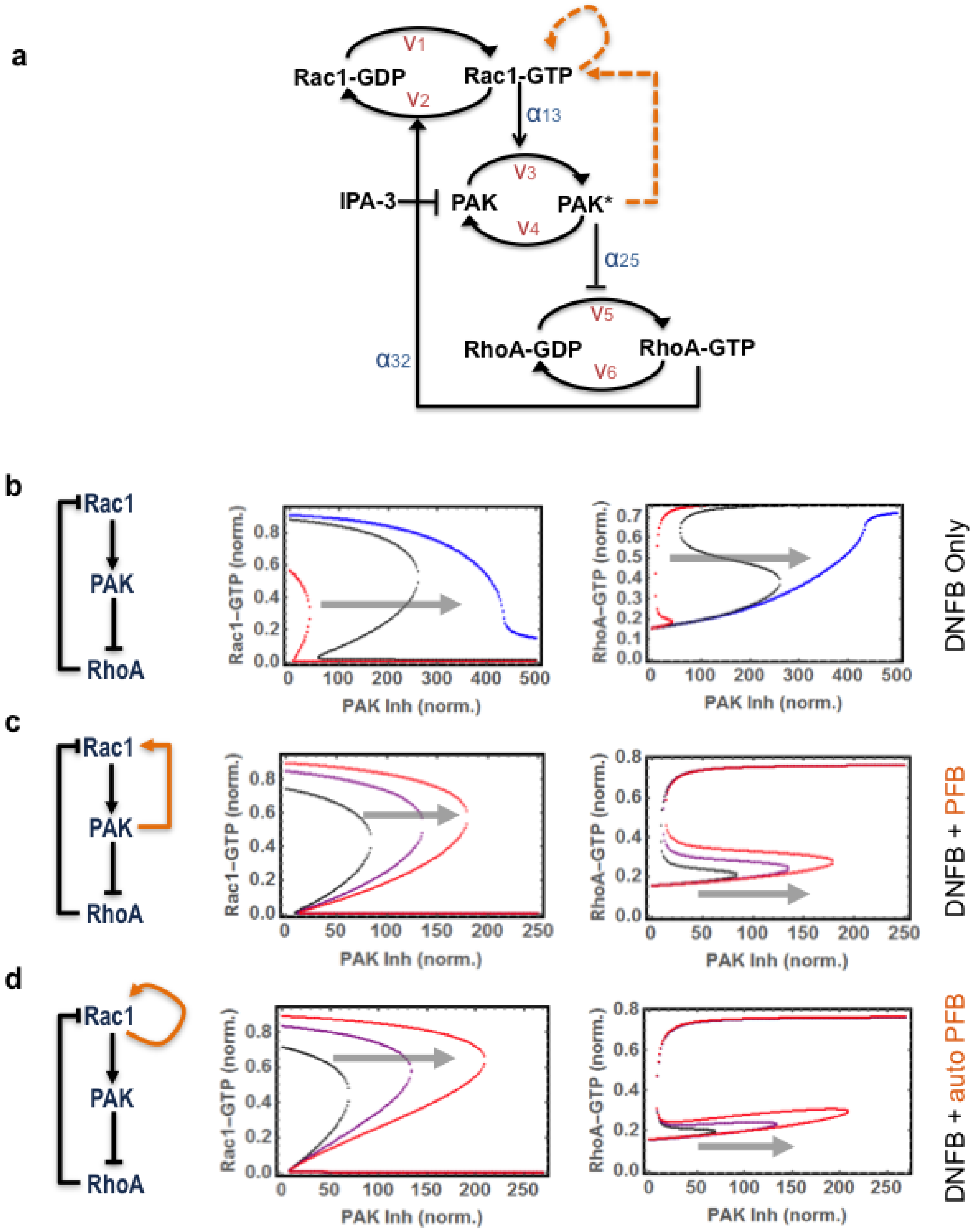
a
Rac1-GDP
Rac1-GTP
V1
V2
α13
IPA-3
PAK
PAK*
V3
V4
α25
RhoA-GDP
RhoA-GTP
V5
V6
α32
b
Rac1
PAK
RhoA
Rac1-GTP (norm.)
RhoA-GTP (norm.)
PAK Inh (norm.)
DNFB Only
c
DNFB + PFB
d
DNFB + auto PFB

Supplemental Figure Legends

Figure S1 Related to Figure 1. Constitutively active RhoA Rac1-GTP **(a)** A constitutively active, EGFP tagged RhoA V14 mutant was transfected into MDA-MB-231 cells. Rac1-GTP was precipitated with GST-PAK-CRIB beads and western blotted. **(b)** Right panel represents the densitometric analysis of three biological replicates

Figure S2. **Related to Figure 2.** Scheme illustrating the ensemble dynamic analysis method DYVIPAC and data representation using Parallel Coordinates Plot. Large sets (typically > 10,000) of selected parameters ($p_1, p_2,..., p_n$ where typically $n \geq 3$) randomly sampled from specified ranges of values. The dynamic behaviour of the modelled system at each set is assessed using linear stability analysis, then classified (e.g. bistable or monostable, etc.) and visualized on the parallel coordinate plot. Note that all the sampling ranges are scaled to the [0,1] range to facilitate comparison and visualization of all sampled sets on the same plot. The whole process can be repeated multiple times for different selected parameters.

Figure S3. **Related to Figure 2.** Different presentation of the multi-dimensional dynamical analysis as in Fig. 2c (main text): **(a)** Here, the bistable sets (purple) are overlaid with a much larger number of monostable sets (green), totalling 100,000 sampled sets. The pink line indicates values measured in MDA-MB-231 cells. **(b)** Here, only the bistable sets are plotted but individual sets are coded with different colours.

Figure S4. **Related to Figure 2.** Comparison of the multi-dimensional dynamical analysis results for 5 parameters as in Fig.2c, main text, for different sampling range. **(a)** All the species totals are varied within 0-1000 nM while in **(b)** All the species totals are varied within 0-5000 nM. The patterns however are similar in both cases. This is further supported as observed in panels **(c)** and **(d)** where the bistable sets projected on 3D with various parameters also show consistent patterns between the small and large sampling range.

Figure S5. **Related to Figure 2.** Effect of kinetic parameters' variation on the bistable region in the 5D protein abundance space (identified in Fig. 2c). **(a)** Parallel coordinate plots showing the 5D bistable region for three representative parameter sets where 10 kinetic model parameters (Km2, Km8, Km12, k3, k2, k5, k7, k8, k10 and k12) are randomly sampled within 50% range around their assumed physiological values. **(b)** Superposition of plots as in panel (a) for 10 different sampled parameter sets, where the bistable region resulting from each set is coded with the same colour. The pink line represents values measured in MDA-MB-231 cells.

Figure S6. **Related to Figure 2.** Multi-dimensional bistability analysis of kinetic parameter variations when the protein abundances in the model are fixed at the values determined in MDA-MB-231 cells. **(a)** Parallel coordinate plot showing occurrence of bistability when 3 Michaelis-Menten constants (Km2, Km8, Km12) are sampled within large ranges containing their nominal values (pink lines). **(b)** Equivalent representation of data in panel (a) in conventional 3D graph. **(c, d)** Similar plots showing occurrence of bistability when more kinetic parameters are simultaneously sampled around their assumed physiological values. These plots together suggest that the bistable behaviour predicted for the MDA-MB-231 cells appears not to be significantly sensitive to changes in other kinetic parameters.

Figure S7. **Related to Figure 2.** Multi-dimensional bistability analysis for 5 independent parameter combinations in the dimensionless model (section S3, SI). These independent parameter combinations are essentially the abundance variables in the original model scaled by selected kinetic parameters, in order to reduce the dimensionality of the original model and mitigate parameter dependency related issues. The plot shows large region of bistability when these scaled independent parameters (the ratio of protein abundances (totals) and the Km/Kd values) are sampled within large ranges spanning 3 orders of magnitudes.

Figure S8. Related to Figure 2. Dependence of bistability on PAK inhibition and the dose-response curves on parameter variations. **(a,b)** 2D bifurcation diagrams showing dependence of bistability on changes in the total level of PAK inhibitor, Inh, and various model parameters. **(c,d)** Ensemble modelling of multiple dose-response curves of active RhoA and Rac1 against increasing Inh for 15 different sets of values of Rac1, RhoA, PAK, GEF-H1 and 14-3-3 totals, which are randomly varied within 2-fold of the reference values measured in MDA-MB-231 cells.

Figure S9. Related to Figure 3. Quantitation of inter-cellular IPA-3 in MDA-MB-231 cells after a drug washout. Intercellular IPA-3 was quantified by mass spectrometry in negative ion mode. We detected IPA-3 as singly charged or doubly charged ion with a mass-over-charge (m/z) of 349.036 or 175.0220 Da. Graph represent the relative concentration of intercellular IPA-3 of two biological replicates at the given time points in minutes.

Figure S10. Related to Figure 3, 4, 5. Workflow diagram of experimental validations

Figure S11. Related to Figure 3. Hysteresis of pMLC and F-Actin in response to PAK inhibition. **(a)** Imaging processing work flow: Phalloidin stain is used to determine the outline of the cells, HCS cell body stain outlines stains the nucleus and cell body and is used to outline the thicker, three dimensional regions of the cell. Hoechst stains the nucleus. Subtracting HCS mask from the F-actin mask reveals the thin, lamellipodial region of the cells, subtracting the nucleus from the F-actin mask reveals the cytoplasmatic region (b) Lamellipodial pMLC in MDA-MB-231 cells is regulated in a bistable manner by PAK. MDA-MB-231 cells were treated with the PAK inhibitor IPA-3 at the indicated concentrations for 80 minutes (blue) or by incubating MDA-MB-231 cells with 7.5 µM IPA-3 for 20 minutes. The inhibitor was subsequently washed out and the cells were incubated for an additional 60 minutes with IPA-3 at the indicated concentrations (red). Graphs represent the median of the lamellipodial average intensity. n>1000, two biological repeats (c) F-actin in MDA-MB-231 cells is regulated in a bistable manner by PAK. MDA-MB-231 cells were treated with the PAK inhibitor IPA-3 at the indicated concentrations for 80 minutes (blue) or by incubating MDA-MB-231 cells with 7.5 µM IPA-3 for 20 minutes. The inhibitor was subsequently washed out and the cells were incubated for an additional 60 minutes with IPA-3 at the indicated concentrations (red). Graphs represent the median of total cell area average intensity. n>1000, two biological repeats

Figure S12. Related to Figure 5. Bistability and bimodality is present in instantaneous cell velocities. **(a)** Preliminary experiments show full migration inhibition occurs at 15 µM. The IPA-3 concentrations used in Fig. 4a-b are different to those in Fig. 3a-d due to batch-to-batch variablility. Preliminary experiments here show that full inhibition of migration occurs at 15 µM, in agreement with Fig. 3c-f. The methods used were those outlined in the main text. (b,c) Bistability and bimodality is present in instantaneous cell velocities **(b)** For the duration of the experiment, the instantaneous velocities exhibit hysteretic response to IPA-3 treatment. The hysteresis and the ensuing bistability are consistent with Fig. 4b in the main text, where velocities were averaged over the entire experiment. **(c)** The instantaneous velocities for all IPA-3 treatments follow a bimodal distribution. At 0 and 7.5 µM IPA-3, all cells have near zero and maximum instantaneous velocities, respectively. At the intermediate treatments, where the system is bistable (1.865, 2.5 and 3.75 µM IPA-3), the distribution of velocities is strongly bimodal. The behaviour is present at each stage of the 12-hour experiment.

Figure S13. Related to Figure 5. PAK inhibition negatively regulates cell migration and Rac1 activity. **(a)** A PAK inhibitor peptide coupled to GFP (PAK^i) or an inactive mutant (PAK^i-2m) was transfected into MDA-MB-231 cells. The migration of individual cells was tracked over 12 hours using the same method as Fig. 4 a, b. The mean speed ±SD of three independent experiments are shown. The asterisk * indicates $P < 0.05$ using a two-tailed, unpaired t–test. **(b)** MDA-MB-231 cells were transfected with PAK^i or PAK^i-2m. Rac1-GTP was precipitated with GST-Pak-CRIB beads and western blotted.

Figure S14. **Related to Figure 2.** Effect of additional positive feedback loops on bistability. **(a)** Schematic diagram of the simplified 3-tier Rac1-PAK-RhoA model described in section S4 of the SI. **(b)** Simulations using the model containing only the double negative feedback loop. Dependence of active Rac1 and RhoA on PAK inhibitor doses for decreasing double negative feedback strength (b_{32}=80, blue; 100, black; and 200, red). Bistability is observed to be more pronounced at stronger feedback and it lost for weak feedback. **(c)** Simulations using the model containing an additional Rac1-PAK positive feedback: Dependence of active Rac1/RhoA on increasing PAK inhibitor for increasing positive feedback strength (b_{21}=2.5, blue; 5, black; and 8, red). **(d)** Simulations conducted in the model containing an additional Rac1 auto-regulatory positive feedback: Dependence of active Rac1 and RhoA on increasing PAK inhibitor for increasing positive feedback strength (b_{11}=2.5, blue; 5, black; and 8, red).

Supplemental Tables:

Table S1. Related to Figure 1. Reactions and reaction rates for mechanistic model.

**Rate number**	**Reactions**	**Reaction Rates**
v_1	$[Rac1{-}GDP] \xrightarrow{[Rac1-GEF]} [Rac1{-}GTP]$	$\frac{k_1 \cdot [Rac1{-}GEF] \cdot [Rac1{-}GDP]}{K_{m1} + [Rac1{-}GDP]}$
v_2	$[Rac1{-}GTP] \xrightarrow{[Rac1-GAP]} [Rac1{-}GDP]$	$\frac{k_2 \cdot [Rac1{-}GAP] \cdot [Rac1{-}GTP]}{K_{m2} + [Rac1{-}GTP]}$
α_{32}		$\alpha_{32} = \frac{1 + \beta_{32} \frac{[RhoA{-}GTP]}{K_{32}}}{1 + \frac{[RhoA{-}GTP]}{K_{32}}}$
v_3	$[Rac1{-}GTP] + [iPAK] \rightarrow [Rac1{-}GTP{-}iPAK]$	$k_3 \cdot [Rac1{-}GTP] \cdot [iPAK]$
v_4	$[Rac1{-}GTP{-}iPAK] \rightarrow [Rac1{-}GTP] + [iPAK]$	$k_4 \cdot [Rac1{-}GTP{-}iPAK]$
v_5	$[Rac1{-}GTP{-}iPAK] \rightarrow [Rac1{-}GTP{-}pPAK]$	$k_5 \cdot [Rac1{-}GTP{-}iPAK]$
v_6	$[Rac1{-}GTP{-}pPAK] \rightarrow [Rac1{-}GTP{-}iPAK]$	$k_6 \cdot [Rac1{-}GTP{-}pPAK]$
v_7	$[Rac1{-}GTP{-}pPAK] \rightarrow [pPAK] + [Rac1{-}GTP]$	$k_7 \cdot [Rac1{-}GTP{-}pPAK]$
v_8	$[GEF{-}H1] \xrightarrow{[pPAK]} [pGEF{-}H1]$	$\frac{k_8 \cdot [pPAK] \cdot [GEF{-}H1]}{K_{m8} + [GEF{-}H1]}$
v_9	$[pGEF{-}H1] \rightarrow [GEF{-}H1]$	$\frac{V_{\max 9} \cdot [pGEF{-}H1]}{K_{m9} + [pGEF{-}H1]}$
v_{10}	$[14{-}3{-}3] + [pGEF{-}H1] \rightarrow [14{-}3{-}3{-}pGEF{-}H1]$	$k_{10} \cdot [14{-}3{-}3] \cdot [pGEF{-}H1]$
v_{11}	$[14{-}3{-}3{-}pGEF{-}H1] \rightarrow [14{-}3{-}3] + [pGEF{-}H1]$	$k_{11} \cdot [14{-}3{-}3{-}pGEF{-}H1]$
v_{12}	$[RhoA{-}GDP] \xrightarrow{[GEF-H1]} [RhoA{-}GTP]$	$\frac{k_{12} \cdot [GEF{-}H1] \cdot [RhoA{-}GDP]}{K_{m12} + [RhoA{-}GDP]}$
v_{13}	$[RhoA{-}GTP] \xrightarrow{[RhoA-GAP]} [RhoA{-}GDP]$	$\frac{k_{13} \cdot [RhoA{-}GAP] \cdot [RhoA{-}GTP]}{K_{m13} + [RhoA{-}GTP]}$
v_{14}	$[IPA{-}3] + [iPAK] \rightarrow [IPA{-}3{-}iPAK]$	$k_{14} \cdot [IPA{-}3] \cdot [iPAK]$
v_{15}	$[IPA{-}3{-}iPAK] \rightarrow [IPA{-}3] + [iPAK]$	$k_{15} \cdot [IPA{-}3{-}iPAK]$
v_{16}	$[pPAK] \rightarrow [iPAK]$	$k_{16} \cdot [pPAK]$

Table S2. Related to Figure 1. Ordinary differential equations of mechanistic model.

**Left hand side**	**Right hand side**
d[Rac1-GDP]/dt	$-v_1 + v_2*\alpha_{32}$
d[Rac1-GTP]/dt	$v_1 - v_2* \alpha_{32} - v_3 + v_4 + v_7$
d[IPA-3]/dt	$-v_{14} + v_{15}$
d[IPA-3-iPAK]/dt	$v_{14} - v_{15}$
d[iPAK]/dt	$-v_3 + v_4 - v_{14} + v_{15} + v_{16}$
d[Rac1-GTP-pPAK]/dt	$v_5 - v_6 - v_7$
d[Rac1-GTP-iPAK]/dt	$v_3 - v_4 - v_5 + v_6$
d[pPAK]/dt	$v_7 - v_{16}$
d[protein14-3-3]/dt	$-v_{10} + v_{11}$
d[GEF-H1]/dt	$-v_8 + v_9$
d[pGEF-H1]/dt	$v_8 - v_9 - v_{10} + v_{11}$
d[pGEF-H1-protein14-3-3]/dt	$v_{10} - v_{11}$
d[RhoA-GDP]/dt	$-v_{12} + v_{13}$
d[RhoA-GTP]/dt	$v_{12} - v_{13}$

Table S3. Related to Figure 1. Protein totals for the mechanistic model.

	**Totals**
$Rac1_{TOT}$	$[Rac1-GTP]+[Rac1-GDP]+[Rac1-GTP-iPAK]+[Rac1-GTP-pPAK]$
$IPA\text{-}3_{TOT}$	$[IPA-3]+[IPA-3-iPAK]$
PAK_{TOT}	$[iPAK]+[IPA-3-iPAK]+[Rac1-GTP-iPAK]+[Rac1-GTP-pPAK]+[pPAK]$
$14\text{-}3\text{-}3_{TOT}$	$[14-3-3]+[14-3-3-pGEF-H1]$
$GEF\text{-}H1_{TOT}$	$[GEF-H1]+[pGEF-H1]+[14-3-3]+[14-3-3-pGEF-H1]$
$RhoA_{TOT}$	$[RhoA-GTP]+[RhoA-GDP]$

Table S4. Related to Figure 1. Parameter values used in the mechanistic model.
Concentrations and the Michaelis-Menten constants (K_m) are given in nM. Protein concentrations are expressed in nM. First- and second-order rate constants are expressed in s^{-1} and $nM^{-1}\ s^{-1}$. Maximum rates V_{max} are expressed in nM s^{-1}. β_{32} and K_{32} are dimensionless parameters.

**Parameter**	**Value**	**References**
k_1	0.06	Estimated based on typical ranges
Rac1-GEF	8.5	(Nagaraj et al., 2011)
K_{m1}	130	Estimated
k_2	0.505	Estimated
Rac1-GAP	15	(Nagaraj et al., 2011)
K_{m2}	200	Estimated
β_{32}	130	(Tsyganov et al., 2012)
K_{32}	1650	(Tsyganov et al., 2012)
k_3	0.0001	Estimated
k_4	0.0002	Estimated
k_5	0.0009	Estimated
k_6	0.00035	Estimated
k_7	0.0004	Estimated
k_8	0.505	Estimated
K_{m8}	30	Estimated
V_{max9}	5.05	Estimated
K_{m9}	20	Estimated
k_{10}	0.0001	Estimated
k_{11}	0.001	Estimated
k_{12}	0.9	Estimated
K_{m12}	170	Estimated
k_{13}	0.06	Estimated
K_{m13}	10	Estimated
RhoA-GAP	10	(Nagaraj et al., 2011)
k_{14}	0.001	Estimated
k_{15}	0.01	Estimated
k_{16}	0.0005	Estimated
$Rac1_{TOT}$	160	Measured in this study
PAK_{TOT}	27	Measured in this study
$RhoA_{TOT}$	161	Measured in this study
IPA-3_{TOT}	0 (or 270) (*)	
GEF-$H1_{TOT}$	4	Measured in this study
14-3-3_{TOT}	1200	Measured in this study

(*) Total IPA-3 = 0 when the model was simulated with no PAK inhibitor. IPA-3 total = 270 nM when the model was simulated with PAK inhibitor (at which the system is bistable, as seen in Fig. 2e,f in the main text).

Table S5. Related to Figure 2. Protein copy numbers and concentrations.

**Gene names**	**Protein name**	**Copy number Intensity**	**Concentration (nM) (*)**
PAK2	Serine/threonine-protein kinase PAK 2	**661,552**	**27**
RAC2	Ras-related C3 botulinum toxin substrate 2	277,301	
RAC1;RAC3	Ras-related C3 botulinum toxin substrate 1;Ras-related C3 botulinum toxin substrate 3	**3,984,262**	**160**
ARHGEF7	Rho guanine nucleotide exchange factor 7	20,973	
ARHGEF12	Rho guanine nucleotide exchange factor 12	3,610	
ARHGEF10	Rho guanine nucleotide exchange factor 10	3,718	
ARHGEF1	Rho guanine nucleotide exchange factor 1;ARHGEF1 protein	124,608	
ARHGEF18	Rho guanine nucleotide exchange factor 18	19,482	
ARHGEF2	Rho guanine nucleotide exchange factor 2	**100,443**	**4**
ARHGEF28	Rho guanine nucleotide exchange factor 28	6,658	
RHOT1	Mitochondrial Rho GTPase 1;Mitochondrial Rho GTPase	7,391	
RHOC	Rho-related GTP-binding protein RhoC	530,247	
RHOA;RHO C	Transforming protein RhoA;Rho-related GTP-binding protein RhoC	**4,013,548**	**161**
RHOG	Rho-related GTP-binding protein RhoG	490,041	
RHOT2	Mitochondrial Rho GTPase 2	7,850	
RHOF	Rho-related GTP-binding protein RhoF	25,597	
YWHAQ	14-3-3 protein theta	3,147,878	
YWHAB	14-3-3 protein beta/alpha	3,602,803	
YWHAB	14-3-3 protein beta/alpha	587,444	
SFN	14-3-3 protein sigma	1,937,124	
YWHAG	14-3-3 protein gamma	385,879	
YWHAE	14-3-3 protein epsilon	9,115,957	
YWHAZ	14-3-3 protein zeta/delta	10,513,216	
YWHAH	14-3-3 protein eta	456,572	
	Total 14-3-3	**29,746,874**	**1200**

(*) Concentrations were calculated assuming an average cell volume = 4 x 10^{-14}(L). It is worth noting that our simulations show that bistablity depends on the relative rather than absolute concentrations of these species; therefore a larger or smaller cell volume will not affect our results.

Supplemental Experimental Procedures.

S1. Construction of a kinetic model for the integrated Rac1-PAK-RhoA pathway.

In this section, we present description and assumptions of a kinetic and dynamic model of the Rac1-PAK-RhoA interaction network used for simulation and analysis in the main text (the model scheme is given in Fig. 1a of the main text). This model was aimed to capture the up-to-date details of the network interactions in light of experimental evidence, incorporating protein-protein interactions, phosphorylation events and feedback regulation. The model was formulated using the laws of mass-action and enzymatic Michaelis-Menten (MM) kinetics. Note that IPA-3 is explicitly used in the following model reactions and equations instead of the general inhibitor INH depicted in Fig. 1.

S1.1. Model description and assumptions

S1.1.1. Activation of PAK by Rac1

Membrane bound Rac1-GTP recruits p21-activated kinases (PAKs) by binding to PAK's Cdc42-Rac interactive binding (CRIB)-domain, causing PAK to undergo a conformational switch. This exposes its activation loop, which is subsequently auto-phosphorylated through an intra-molecular mechanism, resulting in full activation of the kinase (Bokoch, 2003; Zhao and Manser, 2012). The mass-action model reaction scheme is given in Fig. 1a.

S1.1.2. PAK inhibition of RhoA through inhibition of RhoGEF

Activated PAK phosphorylates GEF-H1, a GEF for RhoA, on inactivating inhibitory sites (Zenke et al., 2004) Following phosphorylation, GEF-H1 binds to 14-3-3 protein which causes its relocation to microtubules (Zenke et al., 2004) where it has been shown to have decreased GEF activity (Krendel et al., 2002) In this way, Rac1 inhibits RhoA activity through PAK (Fig. 1a).

S1.1.3. Potential pathways of Rac1 inhibition by RhoA through Rac1 GAPs

We have shown experimentally that expression of constitutively active RhoA (RhoAV14) in MDA-MB-231 cells decreases the amount of active Rac1 (Fig. S1). There are several potential routes for the inhibition of Rac1 by RhoA including through the regulation of RacGAPs, e.g. ARHGAP22 (Sanz-Moreno et al., 2008) and FilGAP (Saito et al., 2012), which are phosphorylated and activated by the Rho effector kinase ROCK. In our kinetic model, we assume that active RhoA deactivates Rac1 via activation of Rac1 GAPs (Fig.1a). In addition, due to the lack of exact mechanistic detail, we describe this negative regulation in a generic way using a dimensionless multiplier that captures Rac1 GAPs activation specifying the mechanism of interaction.

Generally, we denote this multiplier α_{ij}, where the index i refers to the rate equation number being modified, and j refers to the protein that modifies the regulation. This modeling approach and notation follow our previous publication (Tsyganov et al., 2012).

$$\alpha_{ij} = \frac{1 + \beta_{ij} \dfrac{P_j}{K_{ij}}}{1 + \dfrac{P_j}{K_{ij}}} \qquad \text{(S1)}$$

P_j represents the concentration of the protein j. The coefficient β_{ij} determines the maximal degree of regulation. If $\beta_{ij} < 1$, α_{ij} describes downregulation. If $\beta_{ij} > 1$, the term α_{ij} describes upregulation, as in the case of Rac1-GTP inhibition of RhoA-GTP here by upregulation of Rac1-GAPs. K_{ij} is the activation or inhibition constant. In this scheme, Rac1-GTP, pPAK and RhoA-GTP are labelled as proteins 1, 2 and 3 respectively.

In the case where RhoA-GTP (P_3) stimulates the deactivation of Rac1 (rate v_2, TableS1), the corresponding multiplier reads:

$$\alpha_{32} = \frac{1 + \beta_{32} \dfrac{[RhoA\text{–}GTP]}{K_{32}}}{1 + \dfrac{[RhoA\text{–}GTP]}{K_{32}}}$$

S1.1.4. Inhibition of PAK by a chemical inhibitor IPA-3

Our model-based simulations and analysis suggested that PAK perturbation by inhibition is a promising way to reveal bistable behaviour for the network components (see main text). To selectively inhibit PAK in our experiments, we use IPA-3, a specific chemical inhibitor for PAK. It is known that Rac1 activates PAK by binding

directly to PAK's regulatory domain, which relieves its autoinhibition and the resulting conformational change leads to PAK autophosphorylation (Bokoch, 2003). IPA-3 is a highly selective, non-ATP-competitive inhibitor that binds directly to the regulatory domain of inactive PAK1-3 and thus prevents the Rac1 dependent activation of PAKs in a dose dependent manner. Consequently, IPA-3 has a substantially reduced effect on already active PAK (Deacon et al., 2008; Viaud and Peterson, 2009). We modelled the series of steps of IPA-3 mediated inhibition of PAK using mass action kinetics and corresponding reaction rates v_{14}, v_{15}, and v_{16} can be found in Table S1.

S1.3. Selection of parameter values

As many of the kinetic parameters in the Rac1-PAK-RhoA system are unknown at the present time, the parameter values used as the reference set for model analysis were guided by typical ranges of physiological values and constrained by experimental data where possible (Table S4). For example, the association of protein molecules into dimers or larger complexes occurs with typical rate constants of the order of 10^{-4} to 10^{-1} nM^{-1} s^{-1} (Kholodenko et al., 1999) In addition, the reaction rates were always constrained to be not faster than the diffusion limit. Moreover, it is worthwhile to note that a major aim of modelling is to provide a basis for guiding experimental analysis and testing explicit hypotheses; a model by itself is not an objective "truth," but it can be used to falsify or confirm a specific hypothesis. Therefore, comprehensive systematic parameter exploration compatible with experimentally observed behaviour constitutes an appropriate approach to mitigate the lack of measured parameters. This is the approach we adopted in this study.

Since abundances of Rac1, RhoA, PAK and other proteins are not available for MDA-MB-231 cells, plausible concentration ranges were based on those reported by quantitative proteomic studies reported for HeLa (Nagaraj et al., 2011) and U2OS (Beck et al., 2011) cell lines. The cell volumes used for calculation in HeLa and U2OS cell lines were $2.6\text{x}10^3$ μm^3 (Zhao et al., 2008) and $4\text{x}10^3$ μm^3 (Beck et al., 2011) respectively. For each protein, we divide the molecular weight (given in Da or g mol^{-1}) by Avagadro's constant (6.02×10^{23} mol^{-1}) to obtain the mass per molecule in grams. This is then multiplied by the number of copies per cell to obtain the mass of protein per cell, *m*. Therefore the molarity is obtained from the equation

$$C = \frac{p}{V \cdot A}$$

where p is the number of copies per cell, V is the cell volume (L) and A is Avagadro's constant.

For the unspecific Rac1-GEF, Rac1-GAP and RhoA-GAP, concentrations based on the average of several common Rac1 or RhoA GEFs and GAPs, including p114-RhoGEF, ARH-GEF17, RacGAP1, p50-RhoGAP and p105-RhoGAP (Beck et al., 2011; Nagaraj et al., 2011). Selected values for V_{max} and K_m are based on typical values. Values for V_{max} are assumed to be in the range 10^{-1} to 10^2 (Kholodenko et al., 1999). Parameters β_{32} and K_{32} are within parameter ranges used in (Tsyganov et al., 2012). In this paper, the parameter values given are normalized with respect to the total protein concentration and so parameter values are for the normalized activation or inhibition constant $m_{ij} = G_i^{tot}/K_{ij}$, where G_i^{tot} is the total concentration of protein i. As the parameters in our models are not normalized, we obtain values for K_{ij} from the formula $K_{ij}=m_{ij}$* (Tsyganov et al., 2012), using the total concentration ranges described previously.

2. Model dynamical analysis

2.1. Dynamical assessment based on linear stability analysis

Tools of nonlinear dynamics provide a useful framework to assess the dynamical properties of the Rac1-PAK-RhoA model described above (equations in Table S2). We are mainly interested in the asymptotic states and transitions between different dynamic regimes, in particular bistable and non-bistable (mostly fixed point) dynamics in this case. The steady states of the system are obtained by equating the right hand side (RHS) of the system ODEs to zero and solving for the values of the model states' concentrations. An important point to note is that it is convenient here and necessary for the next steps to reduce the full algebraic system of equations to minimal form containing only independent equations using the conditions of concentration conservation (conservation laws). These conditions are the total mass conservation for the state variables Rac1, PAK, RhoA, GEF-H1 and 14-3-3 for the mechanistic model; and Rac1, PAK, RhoA for the phenomenological model.

A solution different from the trivial one (all the species equal to zero) can exist and the temporal evolution of the system can be described by the independent selected species. If we assume that S_{indep} is the vector composed by these independent concentrations, the temporal evolution of the systems is completely determined by

$$\frac{d\boldsymbol{S}_{\text{indep}}}{dt} = f(\boldsymbol{S}_{\text{indep}}), \tag{S2}$$

where $f(S_{\text{indep}})$ is the vector formed by the reaction rates of the independent selected species. The steady states denoted by S^0_{indep} are obtained by solving $f(S^0_{\text{indep}})$=0. The asymptotic stability of these steady states can be determined by linear analysis upon perturbations. Thus, in the vicinity of any of these steady states the temporal evolution of a perturbation from this state, denoted by $\Delta S^0_{\text{indep}} = S_{\text{indep}} - S^0_{\text{indep},}$ is given by

$$\frac{d\Delta\boldsymbol{S}_{\text{indep}}}{dt} = J(\boldsymbol{S}_{\text{indep}})\Delta\boldsymbol{S}^0_{\text{indep}}, \tag{S3}$$

where $J(S^0_{\text{indep}})$ is the Jacobian matrix of the reduced system evaluated at the considered steady state. Thus, the dynamical behavior of the system is entirely specified by the Jacobian matrix and by its eigenvalues and eigenvectors. If we assume that the eigenvalues of $J(S^0_{\text{indep}})$ for a given steady state S^0_{indep}, denoted by λ_i (i=1,2,..n where n is the number of independent species) are ordered decreasingly by the values of their real part, Re(λ_i), then the dominant growth term of the perturbation is governed by

$$\Delta\boldsymbol{S}^0_{\text{indep}}(t) \propto \boldsymbol{E}_1 e^{\text{Re}(\lambda_1)t}, \tag{S4}$$

where E_1 is the eigenvector corresponding to the eigenvalue with the largest real part. The state S^0_{indep} is asymptotically stable if, and only if, Re(λ_1) is negative and $\Delta S^0_{\text{indep}}$ tends to exponentially decrease in the course of time. If there is at least one positive eigenvalues' real part (i.e. at least Re(λ_i) is positive) then the perturbation grows exponentially and S^0_{indep} is unstable in response to the perturbation.

In order to study the asymptotic states and the transition between different dynamical behaviors, we have solved numerically the system for the parameters values given in Tables S4 and S7 (and other explored values mentioned in the text) using Mathematica 8.0. Although linear models can generally be solved analytically, non-linear models like ours cannot usually be solved analytically and resorting to numerical solvers are required. For the Rac1-PAK-RhoA model and the considered parameter ranges, only one or three real positive solutions are possible. Once the solutions have been obtained, we have substituted them into the Jacobian Matrix and numerically calculated their eigenvalues to classify the different dynamical behaviors. As discussed in section S1.3 and in the main text, we have repeated this procedure for many different values of the state variables (species concentrations) and kinetic parameters to obtain a more complete picture of the system dynamics in the parameter space.

When a unique steady state exists, the sign of the largest real part of the eigenvalue associated with that solution allows us to classify that steady state as stable (represented by black in Figs.2b-d) or unstable. The same analysis is possible when three steady states coexist. Evaluating the Jacobian matrix and calculating the eigenvalues for each solution enabled classification of the possible dynamical behaviors. For the considered ranges of parameters, typically two stable and one unstable solution indicating a bistable dynamical behavior is reported (represented by red in Figs.2b-d). The borders separating these different regions correspond to different kind of transitions. The points belonging to the borders separating the cases when one or three solutions exist are called saddle nodes bifurcation.

S2.2. Bifurcation diagrams in low dimension (2D)

To show that bistability exists for a wide range of parameters and is not specific to the reference parameter sets chosen for simulation, we generated a series of 2D bifurcation diagrams using the software XPPaut. XPPaut finds fixed points of a system and tracks them as a parameter is varied, giving lines along which equilibrium points exist for the set of parameters being examined (Ermentrout, 2002; XPPAUT). The system is bistable for all values of the parameters on the x and y axes in the area enclosed by these lines, marked in red (e.g. see Fig.2b,c). The system is monostable for all parameter values outside the enclosed area, marked in black.

S2.3. Multi-dimensional analysis of model dynamics to probe the parameter space

Although the dynamical properties of a dynamic system are often judged based on conventional 2D bifurcation analysis (e.g. using XPPaut and AUTO (Ermentrout, 2002; XPPAUT) and presented on 2D bifurcation diagrams, the fact that usually only two parameters are varied at the same time (while remaining parameters are set at fixed values) poses concrete limitation in our effort to obtain a global, multi-dimensional picture of the systems dynamics.

To overcome this limitation, we employ an ensemble approach called DYVIPAC (Nguyen et al., 2015) where multiple model parameters can be simultaneously sampled; and thus the dynamic behaviour of the studied system can be probed over a much wider region of the multi-dimensional parameter space. Importantly, we adapt the Parallel Coordinates graphs (Inselberg, 1985) as a new way to effectively represent the multi-dimensional data from the ensemble dynamical analysis (Nguyen et al., 2015).

First, we select the parameters for simultaneous analysis, the number of which is not limited (typically at least three). Next, parameter sampling algorithms (e.g. Monte Carlo) are carried out to sample a large number of parameter sets (often tens of thousands) over defined ranges for each parameter in a random, unbiased manner from uniform or loguniform distributions. These defined parameter ranges typically span physiologically relevant values for each parameter, but can also be relaxed from biological constraints to take any plausible value range for exploration purpose. For each parameter set generated, assessment of the network's dynamic property at that set is conducted based on linear stability analysis outlined in section S2.1 above. This analysis enables the generic subdivision of the multi-parameter space into regions with distinct dynamic behaviours, including but not limited to monostability, bistability and oscillations. For our Rac1-PAK-RhoA model, monostable and bistable dyamics are typically obtained. Subsequently, results from such dynamics classification can be effectively visualised in a multi-parameter manner using Parallel Coordinates plots, as illustrated in in Fig.2d and Fig.S4. These plots can be loosely considered as *multi-dimensional bifurcation plots* of systems dynamics, analogous to the 2D bifurcation diagram produced by conventional methods, but for multiple model parameters.

We repeated the above 5D analysis for different sets of the kinetic parameters varied around their assumed physiological values. For multiple parameter sets where 10 kinetic parameters (Km2, Km8, Km12, k3, k2, k5, k7, k8, k10 and k12) are sampled randomly within 50% deviation range around their nominal values, simulations show that the identified bistable region is not significantly affected by these variations (Fig. S5). When we fixed the protein abundances in our model at the values determined in MDA-MB-231 cells and varied the kinetic parameters (in multidimensional kinetic parameter space using DYVIPAC (Nguyen et al., 2015)), we observed that bistability occurs over large ranges of these parameters (Fig. S6).

S3. Dimensionless model of the Rac1-PAK-RhoA system

S3.1. Transformation to the dimensionless model

To minimize issues related to parameter dependencies when analyzing the Rac1-PAK-RhoA systems dynamic properties, we transformed the original ODE system given in Table S2 to a dimensionless form where the protein abundance variables are appropriately scaled to selected Michealis-Menten constants (Kms) or binding affinities (Kds). This resulted in the independent parameter combinations that could be used to determine the bistability region. We then analysed this dimensionless model using DYVIPAC, varying the independent parameters over the ranges spanning three orders of magnitudes of our assumed original parameter values that were based on experimentally measured and estimated values.

For derivation of the dimensionless model, we introduced the following dimensionless variables:

$$\text{dGEF-H1}=\frac{\text{GEF-H1}}{K_{m12}};\ \text{dpGEF-H1}=\frac{\text{pGEF-H1}}{K_{m12}};$$

$$\text{dIPA-3}=\frac{\text{IPA-2}}{K_{m8}};\ \text{dIPA-3-iPAK}=\frac{\text{IPA-3-iPAK}}{K_{m8}};$$

$$\text{diPAK}=\frac{\text{iPAK}}{K_{m8}};\ \text{dpPAK}=\frac{\text{pPAK}}{K_{m8}};$$

$$\text{d14-3-3}=\frac{\text{14-3-3}}{K_{d1110}};\text{d14-3-3-pGEF-H1}=\frac{\text{14-3-3-pGEF-H1}}{K_{m12}};$$

$$\text{dRac1GDP}=\frac{\text{Rac1GDP}}{K_{d43}};\text{dRac1GTP-iPAK}=\frac{\text{Rac1GDP-iPAK}}{K_{d43}};\text{dRac1GTP}=\frac{\text{Rac1GTP}}{K_{d43}};$$

$$\text{dRac1GTP-pPAK}=\frac{\text{Rac1GDP-pPAK}}{K_{m8}};$$

$$\text{dRhoAGDP}=\frac{\text{RhoAGDP}}{K_{m2}}\ \text{and}\ \text{dRhoAGTP}=\frac{\text{RhoAGTP}}{K_{m2}}$$

where the prefix "d" indicates dimensionless variables; $K_{d1110}=k_{11}/k_{10}$ and $K_{d43}=k_4/k_3$ are the dissociation constants of the respective binding reactions. Given these new variables, the conservation laws for the new dimensionless model now becomes:

$$[Rac1\text{–}GTP]+[Rac1\text{–}GDP]+[Rac1\text{–}GTP\text{–}iPAK]+\frac{K_{m8}}{K_{m12}}[Rac1\text{–}GTP\text{–}pPAK]=\text{dRac1}_{\text{TOT}}$$

$$[IPA\text{–}3]+[IPA\text{–}3\text{–}iPAK]=\text{dIPA-3}_{\text{TOT}}$$

$$[iPAK]+[IPA\text{–}3\text{–}iPAK]+\frac{K_{m8}}{K_{m12}}[Rac1\text{–}GTP\text{–}iPAK]+[Rac1\text{–}GTP\text{–}pPAK]+[pPAK]=\text{dPAK}_{\text{TOT}}$$

$$\frac{K_{d1110}}{K_{m12}}[14\text{–}3\text{–}3]+[14\text{–}3\text{–}3\text{–}pGEF\text{–}H1]=\text{d14–3–3}_{\text{TOT}}$$

$$[GEF\text{–}H1]+[pGEF\text{–}H1]+[14\text{–}3\text{–}3]+[14\text{–}3\text{–}3\text{–}pGEF\text{–}H1]=\text{dGEF-H1}_{\text{TOT}}$$

$$[RhoA\text{–}GTP]+[RhoA\text{–}GDP]=\text{dRhoA}_{\text{TOT}}$$

where $\text{dRac1}_{\text{TOT}}=\text{Rac1}_{\text{TOT}}/K_{d43};\text{dIPA-3}_{\text{TOT}}=\text{IPA-3}_{\text{TOT}}/K_{m8};\text{dPAK}_{\text{TOT}}=\text{PAK}_{\text{TOT}}/K_{m8};$ $\text{d14-3-3}_{\text{TOT}}=\text{14-3-3}_{\text{TOT}}/K_{m12};\text{dGEF-H1}_{\text{TOT}}=\text{GEF-H1}_{\text{TOT}}/K_{m12}$ and $\text{dRhoA}_{\text{TOT}}=\text{RhoA}_{\text{TOT}}/K_{m2};$

are the dimensionless total species concentrations.

After substituting these new independent variables into the original ODE system, the ODEs of the dimensionless model have the following forms:

dGEFH1' = ((Vmax9/Km12) *dpGEFH1)/((Km9/Km12) + dpGEFH1) - ((k8*Km8/Km12) *dGEFH1 *dpPAK)/((Km8/Km12) + dGEFH1)

dIPA3iPAK' = -k15 *dIPA3iPAK + (k14*Km8) *dIPA3 *diPAK

dpPAK' = -k16 *dpPAK + k7 *dRac1GTPpPAK

dprotein1433pGEFH1' = (k10*Kd1110) *dpGEFH1 *dprotein1433 - (k10*Kd1110) *dprotein1433pGEFH1

dRac1GTP' = ((k1*Km1/Kd43) *(Rac1GEF/Km1) *dRac1GDP)/(Km1/Kd43 + dRac1GDP) - (k3*Km8) *diPAK *dRac1GTP + (k3*Kd43) *dRac1GTPiPAK + (k7*Km8/Kd43) *dRac1GTPpPAK - ((k2*Km2/Kd43) *(Rac1GAP/Km12) *dRac1GTP *(1/Km2 + (beta32 *dRhoAGTP)/K32))/((Km2/Kd43 + dRac1GTP) *(1/Km2 + dRhoAGTP/K32))

dRac1GTPiPAK' = (k3*Km8) *diPAK *dRac1GTP - k4 *dRac1GTPiPAK - k5 *dRac1GTPiPAK + (k6*Km8/Kd43) *dRac1GTPpPAK

dRac1GTPpPAK' = (k5*Kd43/Km8) *dRac1GTPiPAK - (k6 + k7) *dRac1GTPpPAK

dRhoAGDP' = -(((k12*Km12/Km2) *dGEFH1 *dRhoAGDP)/(Km12/Km2 + dRhoAGDP)) + ((k13*Km13/Km2) *(RhoAGAP/Km13) *dRhoAGTP)/(Km13/Km2 + dRhoAGTP)

and (nominal) parameter values:

k1 = 0.06; Rac1GEF = 8.5; Km1 = 130; k2 = 0.505; Rac1GAP = 15; Km2 = 200; beta32 = 130; K32 = 1650; k3 = 0.0001; k4 = 0.0002; k5 = 0.0009; k6 = 0.00035; k7 = 0.0004; k8 = 0.505; Km8 = 30; Vmax9 = 5.05; Km9 = 20; k10 = 0.0001; k11 = 0.001; k12 = 0.9; Km12 = 170; k13 = 0.06; Km13 = 10; RhoAGAP = 10; k14 = 0.001; k15 = 0.01; k16 = 0.0005; Kd43 = k4/k3; Kd1110 = k11/k10;

S3.2. Bistability analysis of the dimensionless model using DYVIPAC

The transformation of the original model into a dimensionless model not only reduced the model's dimension but also resulted in the independent parameter combinations that determine the bistability region. We carried out dynamical analysis on the dimensionless model using DYVIPAC (Nguyen et al., 2015), varying the dimensionless variables over ranges spanning three orders of magnitudes surrounding the nominal parameter values (shown in S3.1) based on experimentally measured and estimated values. As shown in Fig. S7, bistability is still observed over these large ranges of the new independent parameter values, confirming that bistability is also a dominant feature of the dimensionless system.
Note that bimodal distributions may arise in a number of situations: a purely stochastic genetic switch (Acar et al., 2008), a bistable system with stochastically induced transitions (Samoilov et al., 2005), noisy networks with the sigmoidal response function (Niepel et al., 2009; Ochab-Marcinek and Tabaka, 2010), or even as a result of heterogeneous deterministic oscillations when protein abundances vary between isogenic cells (Dobrzynski, 2012). In our case, the combined modelling and validation experimental data strongly suggest that bimodality arises from bistability.

S4. Modelling the effects of additional positive feedbacks

S4.1. Simplified model of the Rac1-PAK-RhoA system

Rac1 and RhoA are embedded in a wider network of interactions, which were not included in our original models. For example, there is feedback from PAK to upstream Rac1 via the protein Cool-2 (cloned out of library-2, also known as α-PIX) that form a positive feedback between Rac1 and PAK. Specifically, when in dimeric form, Cool-2 can act as a specific GEF for Rac1. Upon dissociation into monomers, a process facilitated by PAK, it can act as a GEF for both Rac1 and Cdc42. This generates a positive effect from PAK to Rac1, closing a positive feedback between Rac1 and PAK ((Baird et al., 2005; Feng et al., 2002). However, adding the positive feedback from PAC to Rac1 to the system with existing double negative feedback only enlarges the bistability range, but did not significantly affect the network behaviour, Fig. S14. Similarly, adding Rac1 auto-regulatory positive loop that is PAK-independent also only intensifies bistable behaviour, but did not significantly alter bistability dynamics (Tsyganov et al., 2012), Fig. S14. Also, the incorporation of GDP dissociation inhibitors (GDIs) in the model only modifies the parameter range where bistability exists, but bistability remains a feature of the Rac1-RhoA system (Nikonova et al., 2013).

In this section, we describe the formulation of a simplified model of the Rac1-PAK-RhoA network which is aimed to facilitate the analysis of the roles of additional positive feedbacks. This model allows incorporation of either a Rac1-PAK positive feedback as well as PAK-independent Rac1 autoregulatory positive feedback loop into the Rac1–RhoA double negative feedback. The schematic diagram of this model is given in Fig. S12a. Model formulation and notation convention follow that described in our previous study (Tsyganov et al., 2012).

Model's ODEs:

$g1p'(t) = a_{11}*a_{21}*w_1 - a_{32}*w_2;$
$g2p'(t) = a_{13}*w_3 - w_4;$
$g3p'(t) = a_{25}*w_5 - w_6;$

Here for convenience, g1p, g2p, g3p represent the dimensionless concentrations of the active Rac1-GTP, active pPAK and active RhoA-GTP, respectively (normalized by the respective total abundances).

The basic reaction rates w_i (i=1-6) are defined as:

$$w_1 = \frac{r_1 \cdot (1 - \mathrm{g1p}(t)) / m_1}{1 + (1 - \mathrm{g1p}(t)) / m_1};$$

$$w_2 = \frac{r_2 \cdot \mathrm{g1p}(t) / m_2}{1 + \mathrm{g1p}(t) / m_2};$$

$$w_3 = \frac{r_3 \cdot (\frac{1 - \mathrm{g2p}(t)}{1 + \mathrm{Inh}}) / m_3}{1 + (\frac{1 - \mathrm{g2p}(t)}{1 + \mathrm{Inh}}) / m_3};$$

$$w_4 = \frac{r_4 \cdot \mathrm{g2p}(t) / m_4}{1 + \mathrm{g2p}(t) / m_4};$$

$$w_5 = \frac{r_5 \cdot (1 - \mathrm{g3p}(t)) / m_5}{1 + (1 - \mathrm{g3p}(t)) / m_5};$$

$$w_6 = \frac{r_6 \cdot \mathrm{g3p}(t) / m_6}{1 + \mathrm{g3p}(t) / m_6};$$

where the parameter Inh represents the normalized concentration of the PAK inhibitor. The modifier terms that describe respective feedbacks are defined as below:

$$a_{11} = \frac{1 + b_{11} \cdot \mathrm{g1p}(t) / m_{11}}{1 + \mathrm{g1p}(t) / m_{11}};$$

$$a_{13} = \frac{1 + b_{13} \cdot \mathrm{g1p}(t) / m_{13}}{1 + \mathrm{g1p}(t) / m_{13}};$$

$$a_{21} = \frac{1 + b_{21} \cdot \mathrm{g2p}(t) / m_{21}}{1 + \mathrm{g2p}(t) / m_{21}};$$

$$a_{25} = \frac{1 + b_{25} \cdot \mathrm{g2p}(t) / m_{25}}{1 + \mathrm{g2p}(t) / m_{25}};$$

$$a_{32} = \frac{1 + b_{32} \cdot \mathrm{g3p}(t) / m_{32}}{1 + \mathrm{g3p}(t) / m_{32}};$$

Here a_{13}, a_{25} and a_{31} describes the positive regulation of PAK (g2p) by active Rac1 (g1p), negative regulation of RhoA (g3p) by active PAK (g2p) and negative regulation of Rac1 (g1p) by active RhoA (g3p), forming the Rac1-PAK-RhoA double-negative feedback loop.

On the other hand, a_{21} describes possible positive regulation of Rac1 by PAK, forming a positive feedback between Rac1 and PAK ($b_{21} > 1$). And a_{11} describes possible auto-positive regulation of Rac1, forming a autoregulatory positive feedback of Rac1 that is PAK independent ($b_{11} > 1$).

Nominal parameter values used for simulations:

$r_1 = 3.122$, $r_2 = 0.074$, $r_3 = 30$, $r_4 = 25$, $r_5 = 12$, $r_6 = 11.3$,
$m_1 = 0.236$, $m_2 = 0.00483$, $m_3 = 0.297$, $m_4 = 0.269$, $m_5 = 0.030$, $m_6 = 0.045$,
$b_{13} = 150$, $m_{13} = 1$,
$b_{32} = 200$, $m_{32} = 1$,
$b_{25} = 0.5$`, $m_{25} = 1$,
Inh = 5

S4.2. Examining the effect of added positive feedbacks

The model simplification and formulation described above allowed us to clearly define the strength of each feedback loops and this easily modulate them *in silico*. In agreement with our previous work (Tsyganov et al., 2012) which investigates a large number of two-tiered GTPase cascade topologies, we found that in this three-tiered Rac1-PAK-RhoA system, both the double negative feedback mediated via Rac1-PAK-RhoA and the Rac1-

PAK positive feedback, in principle, can bring bistability on their own. However, adding the positive feedback to the system with existing double negative feedback only enlarges the bistability range, but did not significantly affect the network behaviour (Fig.S12c). Similarly, adding a hypothetical Rac1 auto-regulatory positive that is PAK-independent also only intensify bistability but did not significantly alter bistability dynamics.

S5. Materials and Methods

Cells and reagents. Cells were cultured in DMEM supplemented with 2 mM glutamine and 10% foetal calf serum. Plasmids were transfected with Lipofectamine2000 using the manufacturer's instructions (Invitrogen, UK). Plasmids for PAK^i and PAK^i-2m-eGFP and PAK^i-2m were kindly provided by Debbie Yablonski, GST-Rhotekin-RBD by Mike Olson, GST-Pak-CRIB by Piero Crespo. Antibodies for RhoA were from Santa Cruz (Clane, UK); Rac1 from Millipore (Watford UK), for EGFP from Cell Signalling (Hitchin, UK), IPA-3 was from Merk Millipore (Watford UK).

Cell treatment, lysis and pulldown assays. Cells seeded in collagen-coated plates were either pretreated with 15 μM IPA-3 for 20 minutes or incubated with IPA-3 (0-15 μM) for 40 minutes. The IPA-3 containing media from the pretreated cells was removed and the cells were washed 2 times with 10% FCS DMEM and subsequently incubated in 10% FCS DMEM containing IPA-3 (0-15 μM) for an additional 20 minutes in 10 % FCS-DMEM. Cells were lysed in ice-cold lysis buffer (20 mM HEPES pH7.5, 150 mM NaCl, 1% NP40, 2 mM EDTA) supplemented with protease inhibitors (1 mM PMSF, 5 μg/ml leupeptin, 2.2 μg/ml aprotinin, 2 mM sodium fluoride) and 10 mM $MgCl_2$ (only in pull-down assays). Lysates were cleared of debris by centrifugation at 20,000 g for 10 minutes in a benchtop centrifuge. Cleared lysates were incubated with either 5 μl GST-Rhotekin- or GST-Pak-CRIB-beads for 30 min at 4ºC under end-to-end rotation (for pull-down assays) or boiled in Laemmli buffer (for RhoA/Rac1 input). The beads were washed, boiled in Laemmli buffer and Western blotted. The Western blot bands were quantified using ImageJ. Graphs represent RhoA-GTP/input RhoA, Rac1-GTP/input Rac1.

Motility assays. MDA-MB-231 cells were seeded at 15,000 cells/ml in collagen-coated 12-well plates. After treatment with IPA-3, cells were imaged using a Zeiss Axiovert 200M at 10x, using Andor iQ software, over 12 hours in a temperature and CO_2-controlled environmental chamber. Images were taken every 20 minutes. Individual cells were manually tracked using the Manual Tracking Plugin in ImageJ 1.44o software package. The Manual Tracking plugin gives the position (in pixels) of each cell at each time step and uses this to calculate the speed of each cell per time step (20 minutes). The paths taken by 25 cells under each treatment were plotted in Fig. 4a from the position readouts using MATLAB (Inc., 2010). The average speed of individual cells was calculated over 12 hours and these were used to calculate the average speed per well. For wound healing assays, cells were plated in 6-well dishes and grown to confluence. The cell layer was subsequently scratched with a pipette tip. Three positions were marked on each plate and photographed immediately and after 18h. The wound closure was measured manually on the images.

Cell morphology assays. 0.5 ml of rat-tail collagen I was polymerised in 12-well dishes. 500 MDA-MB-231 cells were seeded and were let to adhere and invade into the collagen gel. 24 hours after seeding the cells were either pretreated with 7.5 μM IPA-3 for 20 minutes or incubated with IPA-3 (0-7.5 μM). The IPA-3 containing media from the pretreated cells was removed and the cells were washed 2 times by incubating the collagen plug for 2 minutes with 10% FCS DMEM and subsequently incubated in 10% FCS DMEM containing IPA-3 (0-7.5 μM). The cells were imaged 24 hours after the treatment by using an Incucyte ZOOM. Cell masks of individual cells were detected using the Incucyte analysis software and cell roundness was measured using ImageJ.

Actin dynamics imaging. *LifeAct-mCherry probe* was delivered to the cells using pHIV lentiviral transfection. This probe was generously provided by Prof. Olivier Pertz (Department of Biomedicine, University of Basel). Cells were seeded and treated as described above for motility assays. The probe expressing cells were imaged at 15 sec intervals for 1 hour, using a Nikon Plan Apo 40x/1.5 DIC oil objective on a spinning-disk laser confocal Nikon microscope with Andor iXonEM+ EMCCD camera, resulting in an effective pixel size of 234 nm. The 561 nm excitation laser and 610 nm emission filter were used. Montage images and movies were created using ImageJ software.

Cellular pMLC imaging. 2000 MDA-MB-231 cells were seeded in collagen I coated polymer optical-bottomed 96-well dishes (Thermo Fisher). 24 hours later cells were either pretreated with 7.5 μM IPA-3 for 20 minutes or incubated with IPA-3 (0-7.5 μM) for 80 minutes. The IPA-3 containing media from the pretreated cells was removed and the cells were washed 2 times with 10% FCS DMEM and subsequently incubated in 10% FCS DMEM containing IPA-3 (0-7.5 μM) for an additional 60 minutes in 10 % FCS-DMEM. Post-treatment the cells were fixed in 3.7% Formaldehyde (PIPES 100 mM pH 6.8, EGTA 10 mM, MgCl2 10 mM, Triton X-100 0.2 %) for 10 minutes. F-actin was stained with Phalloidin 594 nm (Molecular Probes, 1:250 dilution), nucleus by

Hoechst (Invitrogen, 1:5000 dilution), pMLC by pSer19-Myosin Light Chain 2 (Cell signalling, 1/200 dilution) and Goat-anti-Mouse 488 nm Alexa secondary (Invitrogen, 1/200 dilution) and the cell body with HCS cell mask deep red (Invitrogen, 1:150,000 dilution). Cells were imaged on an ImageXpress Micro widefield microscope and images analysed using the MetaXpress Custom Module Editor (Molecular Devices). Briefly, using a local thresholding image analysis technique, we generated binary masks of the nuclei, whole cell mask (derived from the Phalloidin labelling) and cell body mask (from the HCS stain) for each cell. Subtraction of the cell body mask from the whole cell mask gave us a third mask of the lamellipodia area of each cell. Subtraction of the nuclear mask from the whole cell mask allowed us to measure cytoplasmic Phalloidin staining (Supplemental Figure S11a). We then measured the integrated mean fluorescent intensity of the pMLC or phalloidin labelling in each of the masks measuring >2000 cells per treatment.

Quantitative mass spectrometry. MDA-MB-231 cells were lysed in 1% SDS and lysates were washed, reduced, alkylated, digested with trypsin and analysed on a Q-Exactive mass spectrometer as previously reported (Farrell et al., 2014). Proteins were identified and quantified by using the MaxQuant software suite (Cox et al., 2011) by searching against the human swissprot database, with Carbamylations of Cysteine as fixed and N-terminal acetylation and Methionine oxidation as variable modifications. The absolute cellular protein concentration was calculated by the Perseus software suite with the proteome ruler method (Wisniewski et al., 2014). Expression data is summarised in Table S6 and the data are available via ProteomeXchange with identifier PXD003213.

Determining retention time of cellular IPA-3. MDA-MB-231 cells were seeded in collagen coated 6-well dishes and incubated with 7.5 μM IPA-3 for 0-20 minutes. The IPA-3 containing media was removed and the cells were washed three times with ice-cold PBS. Inter-cellular IPA-3 was extracted by scraping the cells with cold methanol. Samples were cleared of cell debris by centrifugation and the methanol was removed with a rotary vacuum concentrator. IPA-3 was detected on a Thermo QExactive using ZIC pHILIC 20 x 2.1 mm guard and 150 x 4.6 mm column (SeQuant) with a 10 minute gradient from 90% to 5% acetonitrile against 20 mM ammonium carbonate, in negative SIM mode, monitoring 348.6 to 349.5 m/z at resolution 70,000, AGC target 3e6 and max IT 200 milliseconds. Peak detection and integration was performed using Thermo Xcalibur 3.0.63.

Supplemental References:

Acar, M., Mettetal, J.T., and van Oudenaarden, A. (2008). Stochastic switching as a survival strategy in fluctuating environments. Nature genetics *40*, 471-475.

Baird, D., Feng, Q., and Cerione, R.A. (2005). The Cool-2/alpha-Pix protein mediates a Cdc42-Rac signaling cascade. Curr Biol *15*, 1-10.

Beck, M., Schmidt, A., Malmstroem, J., Claassen, M., Ori, A., Szymborska, A., Herzog, F., Rinner, O., Ellenberg, J., and Aebersold, R. (2011). The quantitative proteome of a human cell line. Mol Syst Biol *7*, 549.

Bokoch, G.M. (2003). Biology of the p21-activated kinases. Annu Rev Biochem *72*, 743-781.

Cox, J., Neuhauser, N., Michalski, A., Scheltema, R.A., Olsen, J.V., and Mann, M. (2011). Andromeda: A Peptide Search Engine Integrated into the MaxQuant Environment. Journal of proteome research *10*, 1794-1805.

Deacon, S.W., Beeser, A., Fukui, J.A., Rennefahrt, U.E., Myers, C., Chernoff, J., and Peterson, J.R. (2008). An isoform-selective, small-molecule inhibitor targets the autoregulatory mechanism of p21-activated kinase. Chem Biol *15*, 322-331.

Dobrzynski, M., Fey, D., Nguyen L. K. & Kholodenko, B. N. (2012). Bimodal Protein Distribution in Heterogeneous Oscillating Systems. . In Computational Methods in Systems Biology Lecture Notes in Computer Science, D.H.M. Gilbert, ed. (Springer-Verlag Berlin Heidelberg), pp. 17-28.

Ermentrout, B. (2002). Simulating, Analyzing, and Animating Dynamical Systems: A Guide to XPPAUT for Researchers and Students (Philadelphia, USA: SIAM).

Farrell, J., Kelly, C., Rauch, J., Kida, K., Garcia-Munoz, A., Monsefi, N., Turriziani, B., Doherty, C., Mehta, J.P., Matallanas, D*., et al.* (2014). HGF induces epithelial-to-mesenchymal transition by modulating the mammalian hippo/MST2 and ISG15 pathways. Journal of proteome research *13*, 2874-2886.

Feng, Q., Albeck, J.G., Cerione, R.A., and Yang, W. (2002). Regulation of the Cool/Pix proteins: key binding partners of the Cdc42/Rac targets, the p21-activated kinases. J Biol Chem *277*, 5644-5650.

Inc., T.M. (2010). MATLAB (Natick, Massachusetts).

Inselberg (1985). The Plane with Parallel Coordinates. Visual Computer *1*, 69–91.

Kholodenko, B.N., Demin, O.V., Moehren, G., and Hoek, J.B. (1999). Quantification of short term signaling by the epidermal growth factor receptor. The Journal of biological chemistry *274*, 30169-30181.

Krendel, M., Zenke, F.T., and Bokoch, G.M. (2002). Nucleotide exchange factor GEF-H1 mediates cross-talk between microtubules and the actin cytoskeleton. Nature cell biology *4*, 294-301.

Nagaraj, N., Wisniewski, J.R., Geiger, T., Cox, J., Kircher, M., Kelso, J., Paabo, S., and Mann, M. (2011). Deep proteome and transcriptome mapping of a human cancer cell line. Mol Syst Biol *7*, 548.

Nguyen, L.K., Degasperi, A., Cotter, P., and Kholodenko, B.N. (2015). DYVIPAC: an integrated analysis and visualisation framework to probe multi-dimensional biological networks. Scientific Reports *(in press)*.
Niepel, M., Spencer, S.L., and Sorger, P.K. (2009). Non-genetic cell-to-cell variability and the consequences for pharmacology. Current opinion in chemical biology *13*, 556-561.
Nikonova, E., Tsyganov, M.A., Kolch, W., Fey, D., and Kholodenko, B.N. (2013). Control of the G-protein cascade dynamics by GDP dissociation inhibitors. Mol Biosyst *9*, 2454-2462.
Ochab-Marcinek, A., and Tabaka, M. (2010). Bimodal gene expression in noncooperative regulatory systems. Proceedings of the National Academy of Sciences of the United States of America *107*, 22096-22101.
Saito, K., Ozawa, Y., Hibino, K., and Ohta, Y. (2012). FilGAP, a Rho/Rho-associated protein kinase-regulated GTPase-activating protein for Rac, controls tumor cell migration. Mol Biol Cell *23*, 4739-4750.
Samoilov, M., Plyasunov, S., and Arkin, A.P. (2005). Stochastic amplification and signaling in enzymatic futile cycles through noise-induced bistability with oscillations. Proceedings of the National Academy of Sciences of the United States of America *102*, 2310-2315.
Sanz-Moreno, V., Gadea, G., Ahn, J., Paterson, H., Marra, P., Pinner, S., Sahai, E., and Marshall, C.J. (2008). Rac activation and inactivation control plasticity of tumor cell movement. Cell *135*, 510-523.
Tsyganov, M.A., Kolch, W., and Kholodenko, B.N. (2012). The topology design principles that determine the spatiotemporal dynamics of G-protein cascades. Mol Biosyst *8*, 730-743.
Viaud, J., and Peterson, J.R. (2009). An allosteric kinase inhibitor binds the p21-activated kinase autoregulatory domain covalently. Mol Cancer Ther *8*, 2559-2565.
Wisniewski, J.R., Hein, M.Y., Cox, J., and Mann, M. (2014). A "proteomic ruler" for protein copy number and concentration estimation without spike-in standards. Molecular & cellular proteomics : MCP *13*, 3497-3506.
XPPAUT http://www.math.pitt.edu/~bard/xpp/xpp.html.
Zenke, F.T., Krendel, M., DerMardirossian, C., King, C.C., Bohl, B.P., and Bokoch, G.M. (2004). p21-activated kinase 1 phosphorylates and regulates 14-3-3 binding to GEF-H1, a microtubule-localized Rho exchange factor. J Biol Chem *279*, 18392-18400.
Zhao, L., Kroenke, C.D., Song, J., Piwnica-Worms, D., Ackerman, J.J., and Neil, J.J. (2008). Intracellular water-specific MR of microbead-adherent cells: the HeLa cell intracellular water exchange lifetime. NMR Biomed *21*, 159-164.
Zhao, Z.S., and Manser, E. (2012). PAK family kinases: Physiological roles and regulation. Cell Logist *2*, 59-68.
