# Supplementary figures and images for: Bistability in the Rac1, PAK, and RhoA Signaling Network Drives Actin Cytoskeleton Dynamics and Cell Motility Switches
Source: Cell Syst. 2016 Jan 27;2(1):38–48. doi: 10.1016/j.cels.2016.01.003 (PMC4802415; doi:10.1016/j.cels.2016.01.003)

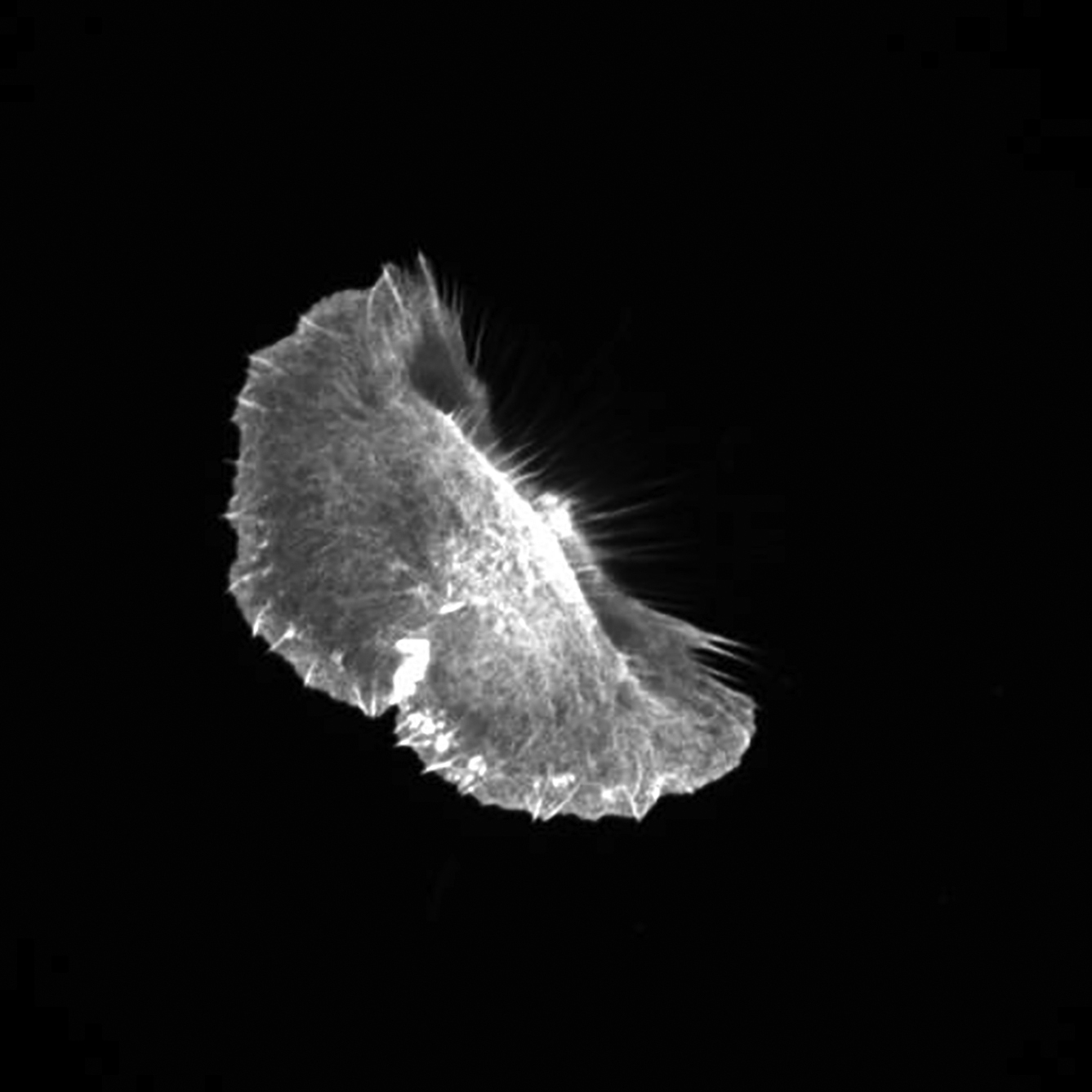

Supplement: Movie S1. Time Lapse of Actin Dynamics of Untreated Cells, Related to Figure 4 [file mmc3.jpg]

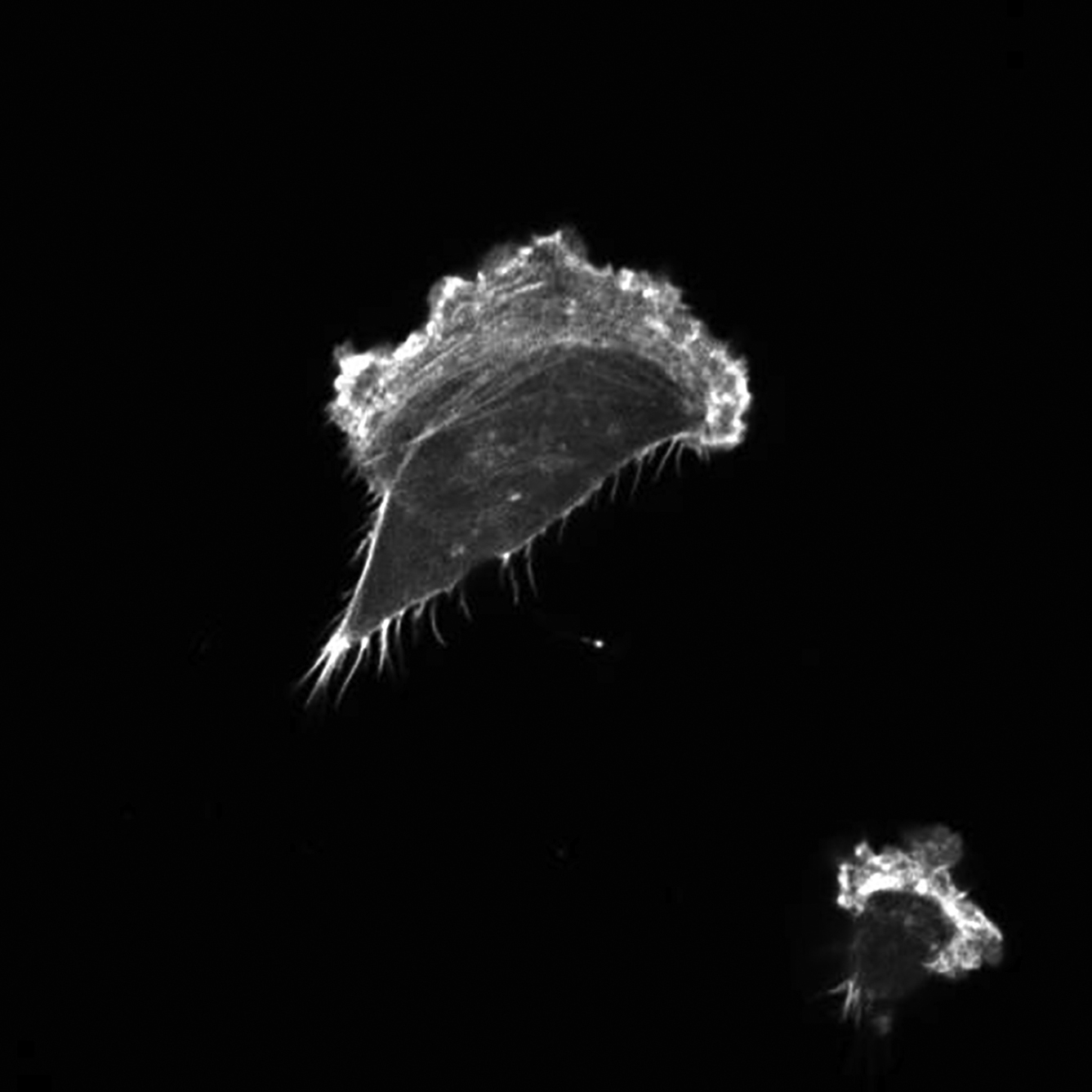

Supplement: Movie S2. Time Lapse of Actin Dynamics Cells Treated with 1.875 μM IPA-3, Related to Figure 4 [file mmc4.jpg]

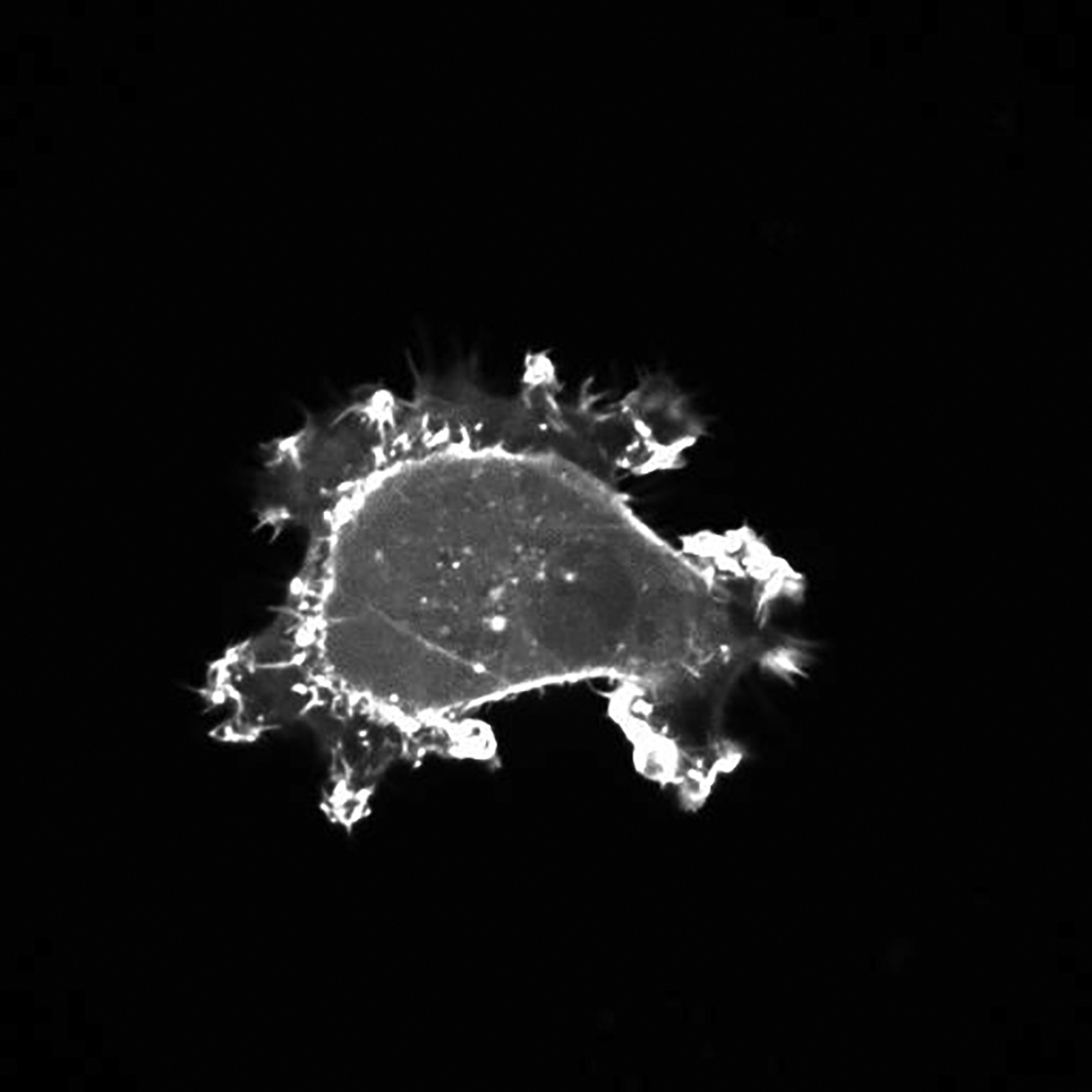

Supplement: Movie S3. Time Lapse of Actin Dynamics Cells Treated with 3.75 μM IPA-3, Related to Figure 4 [file mmc5.jpg]

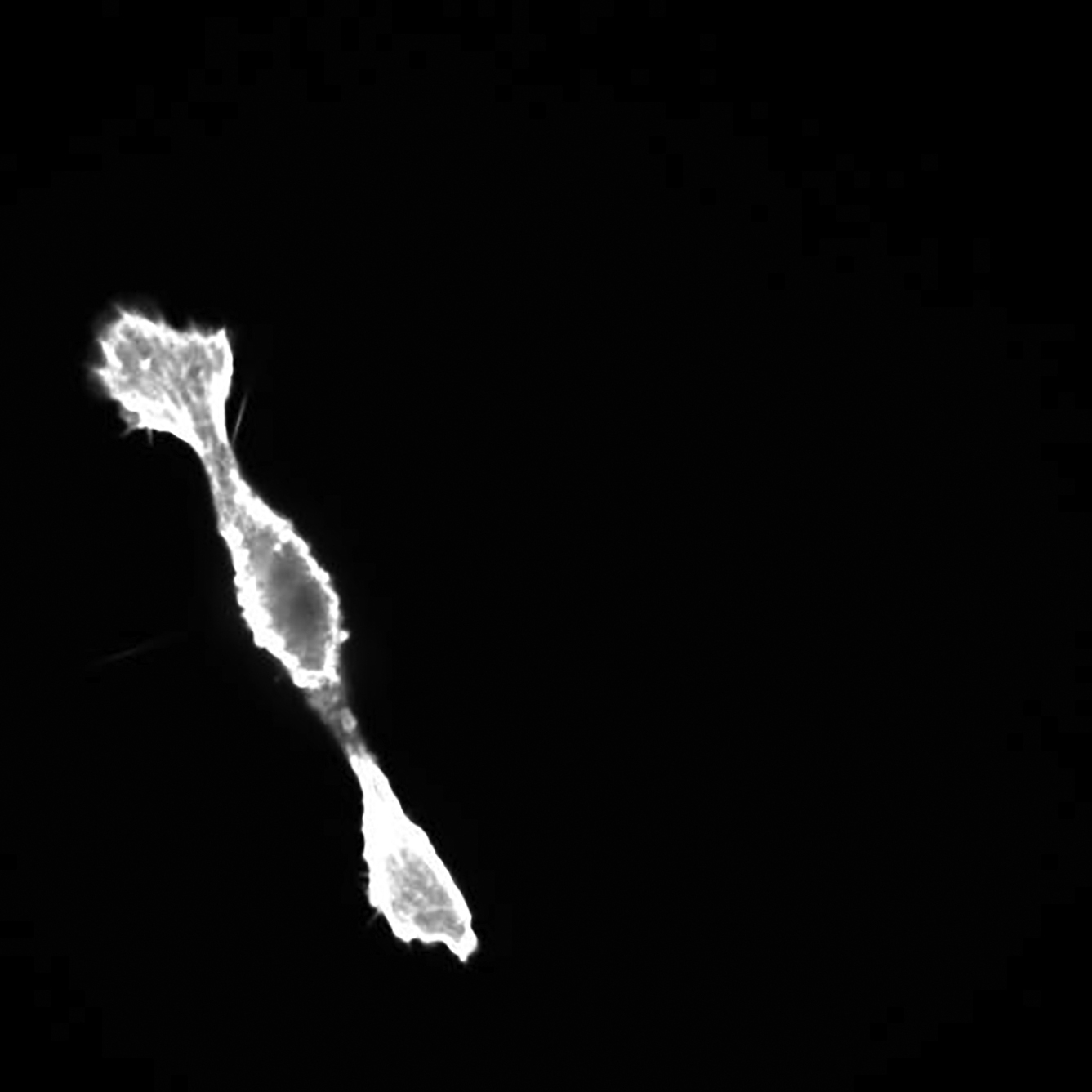

Supplement: Movie S4. Time Lapse of Actin Dynamics Cells Treated with 7.5 μM IPA-3, Related to Figure 4 [file mmc6.jpg]

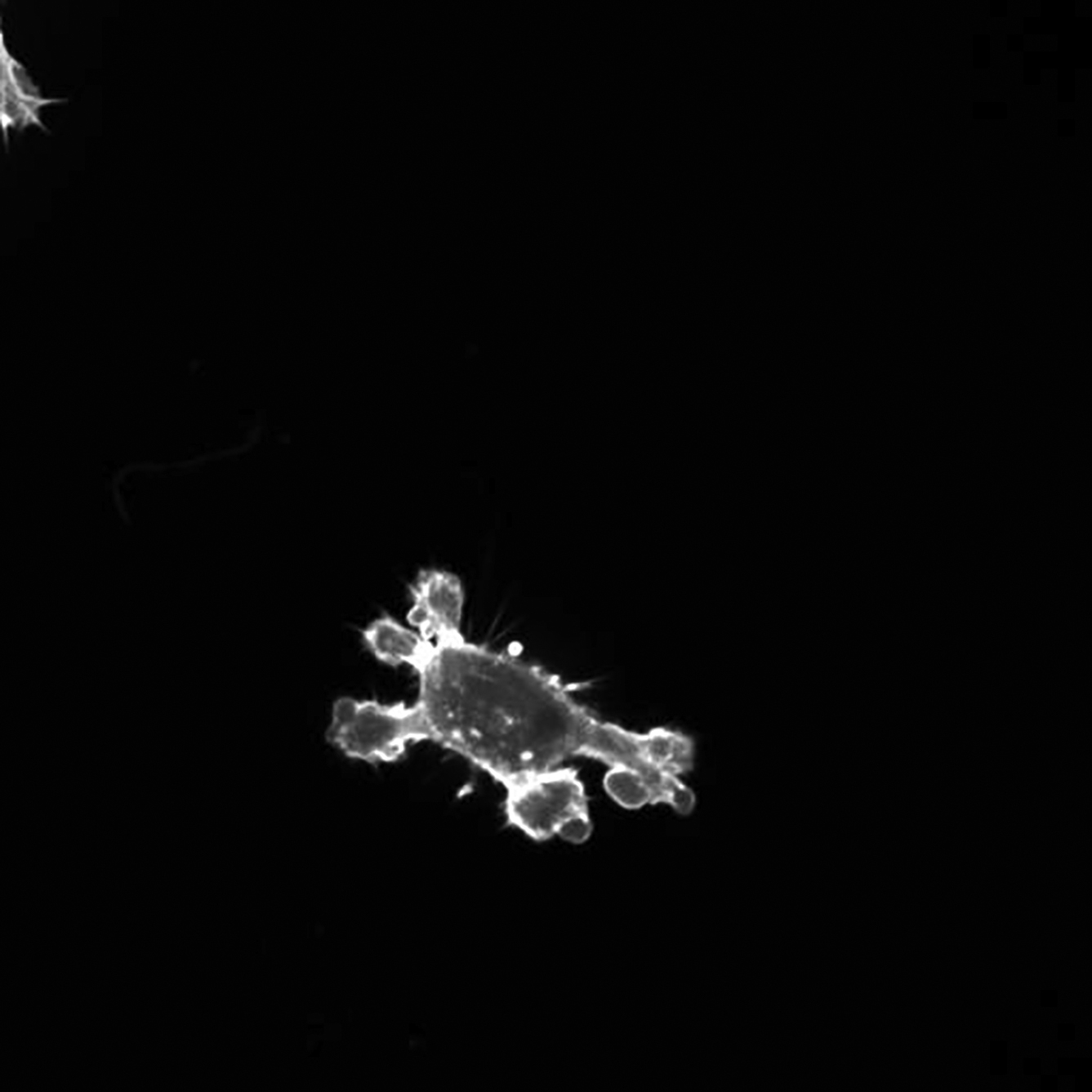

Supplement: Movie S5. Time Lapse of Actin Dynamics Cells Pre-treated with 7.5 μM and Then 0 μM IPA-3, Related to Figure 4 [file mmc7.jpg]

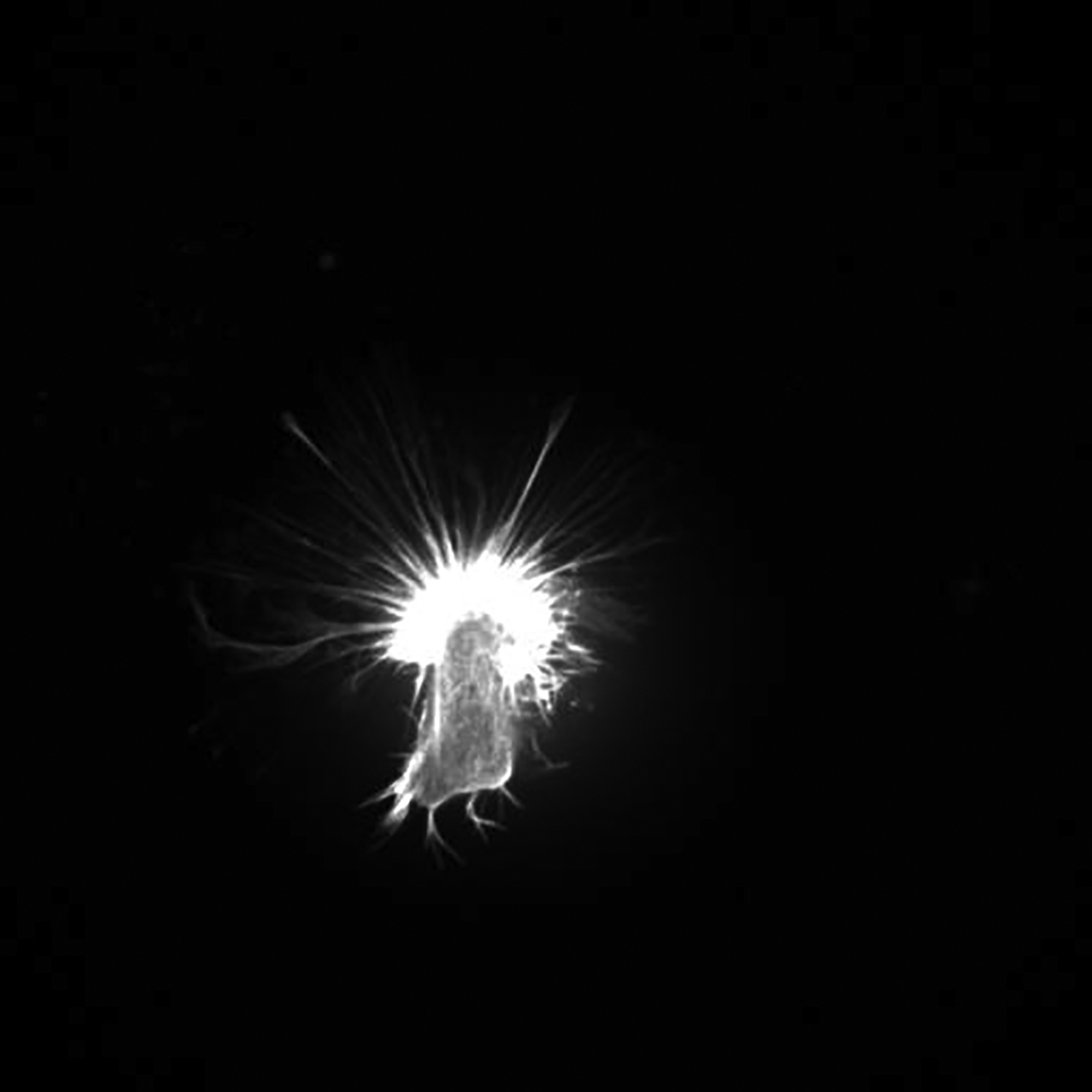

Supplement: Movie S6. Time Lapse of Actin Dynamics Cells Pre-treated with 7.5 μM and Then 1.875 μM IPA-3, Related to Figure 4 [file mmc8.jpg]

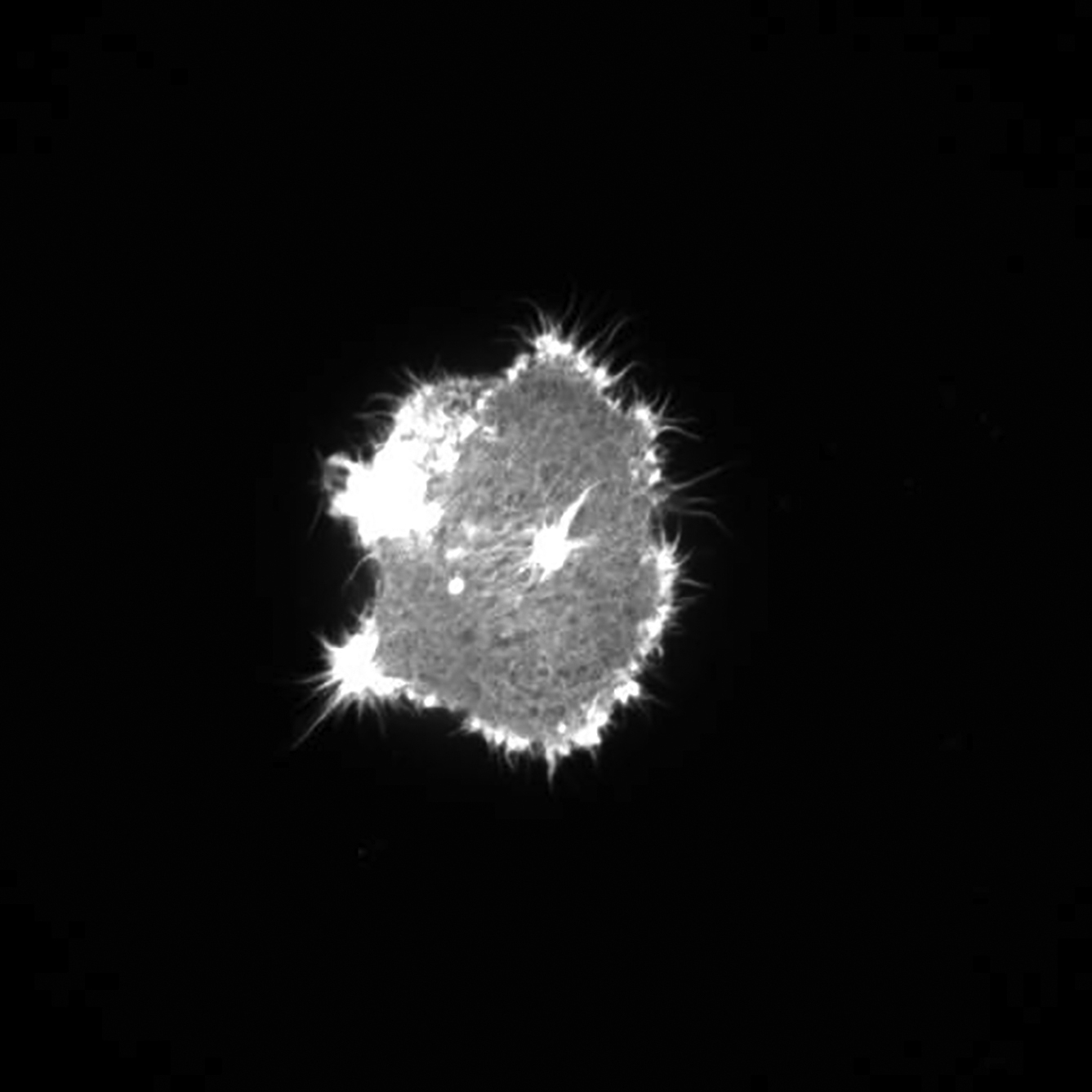

Supplement: Movie S7. Time Lapse of Actin Dynamics Cells Pre-treated with 7.5 μM and Then 3.75 μM IPA-3, Related to Figure 4 [file mmc9.jpg]

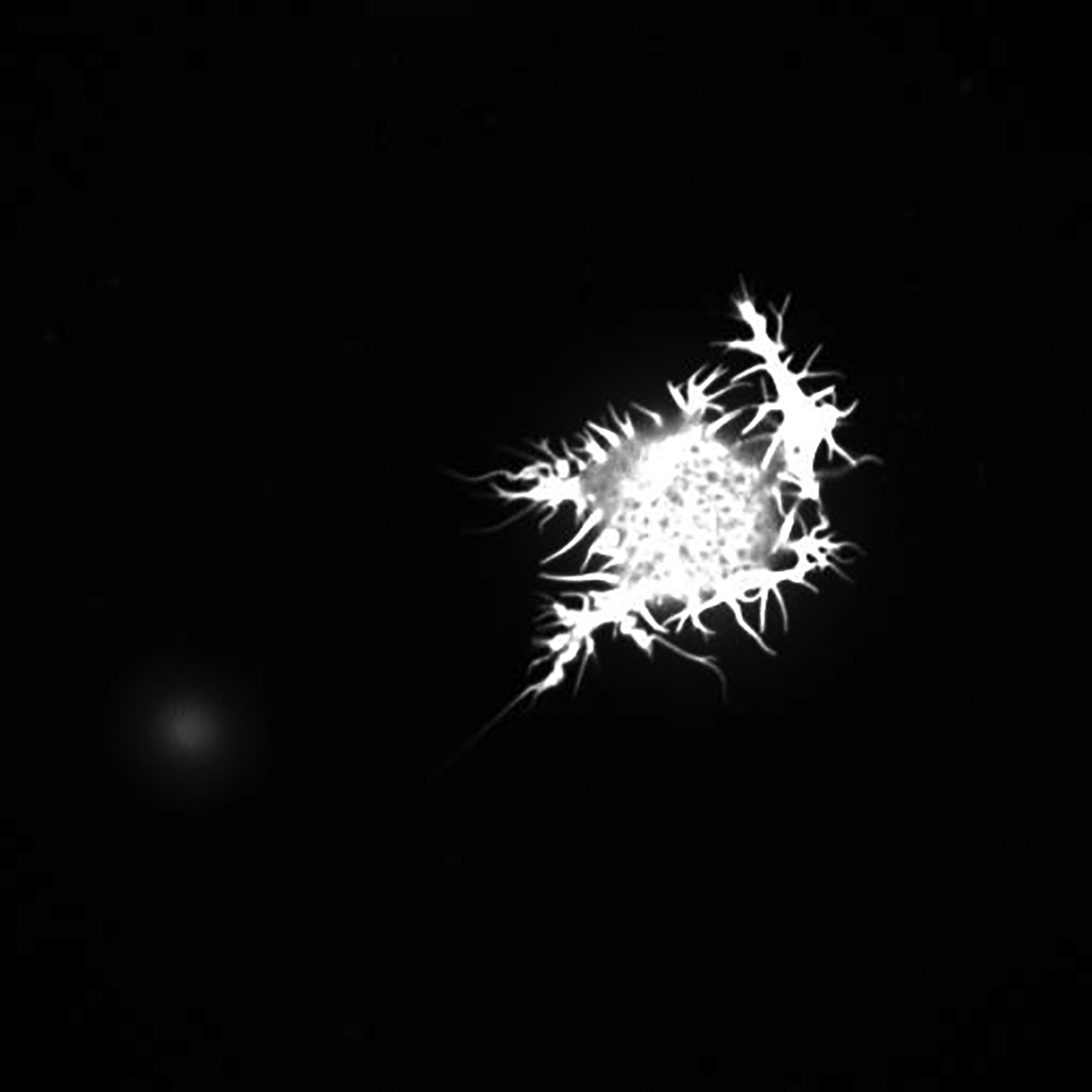

Supplement: Movie S8. Time Lapse of Actin Dynamics Cells Pre-treated with 7.5 μM and Then 7.5 μM IPA-3, Related to Figure 4 [file mmc10.jpg]
