# Supplementary material for: Bistability in the Rac1, PAK, and RhoA Signaling Network Drives Actin Cytoskeleton Dynamics and Cell Motility Switches
Source: Cell Syst. 2016 Jan 27;2(1):38–48. doi: 10.1016/j.cels.2016.01.003 (PMC4802415; doi:10.1016/j.cels.2016.01.003)
Supplement: Document S2. Article plus Supplemental Information [file mmc11.pdf]

Cell Systems

Article

# Bistability in the Rac1, PAK, and RhoA Signaling Network Drives Actin Cytoskeleton Dynamics and Cell Motility Switches

## Graphical Abstract

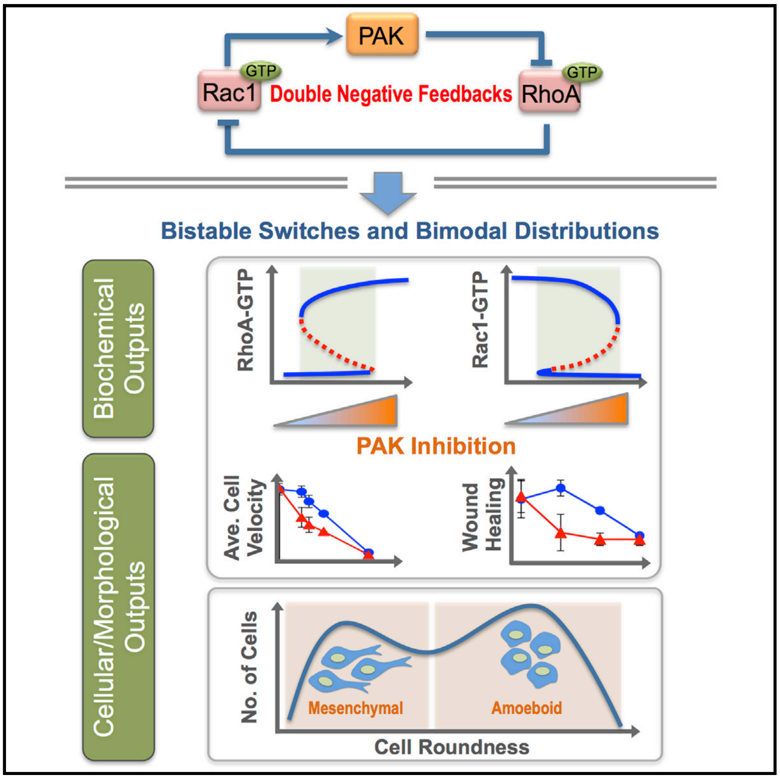


## Highlights

- RhoA and Rac1 are linked by a double-negative feedback loop
- A model predicts bistability of the system within a physiological parameter range
- Rac1 and RhoA activity is bistable in response to PAK inhibition
- Actin dynamics, cell morphology, and migration show hysteresis upon PAK inhibition

## Authors

Kate M. Byrne, Naser Monsefi, John C. Dawson, ..., Lan K. Nguyen, Alex von Kriegsheim, Boris N. Kholodenko

## Correspondence

lan.k.nguyen@monash.edu (L.K.N.), alex.vonkriegsheim@igmm.ed.ac.uk (A.v.K.)

## In Brief

A mathematical model and experiments demonstrate that RhoA, Rac1, actin dynamics, cell morphology, and migration respond in a bistable manner to perturbations.

Byrne et al., 2016, Cell Systems 2, 38–48
January 27, 2016 ©2016 The Authors
http://dx.doi.org/10.1016/j.cels.2016.01.003

CellPress

OPEN ACCESS
CellPress

Cell Systems
Article

# Bistability in the Rac1, PAK, and RhoA Signaling Network Drives Actin Cytoskeleton Dynamics and Cell Motility Switches

Kate M. Byrne,[1] Naser Monsefi,[1] John C. Dawson,[2] Andrea Degasperi,[1] Jimi-Carlo Bukowski-Wills,[2] Natalia Volinsky,[1] Maciej Dobrzyński,[1] Marc R. Birtwistle,[3] Mikhail A. Tsyganov,[4] Anatoly Kiyatkin,[5] Katarzyna Kida,[1] Andrew J. Finch,[2] Neil O. Carragher,[2] Walter Kolch,[1,6,8] Lan K. Nguyen,[1,7,9,*] Alex von Kriegsheim,[1,2,7,8,*] and Boris N. Kholodenko[1,6,8,9]
[1]Systems Biology Ireland, University College Dublin, Belfield, Dublin 4, Ireland
[2]Edinburgh Cancer Research Centre, IGMM, University of Edinburgh, Edinburgh EH4 2XR, UK
[3]Department of Pharmacology and Systems Therapeutics, Icahn School of Medicine at Mount Sinai, New York, NY 10029, USA
[4]Institute of Theoretical and Experimental Biophysics, 142290 Pushchino, Moscow Region, Russia
[5]University of Pennsylvania, Philadelphia, PA 19104, USA
[6]Conway Institute, University College Dublin, Belfield, Dublin 4, Ireland
[7]Co-senior author
[8]School of Medicine and Medical Science, University College Dublin, Belfield, Dublin 4, Ireland
[9]Department of Biochemistry and Molecular Biology, Biomedicine Discovery Institute, Monash University, Melbourne, VIC 3800, Australia
*Correspondence: lan.k.nguyen@monash.edu (L.K.N.), alex.vonkriegsheim@igmm.ed.ac.uk (A.v.K.)
http://dx.doi.org/10.1016/j.cels.2016.01.003
This is an open access article under the CC BY license (http://creativecommons.org/licenses/by/4.0/).

## SUMMARY

**Dynamic interactions between RhoA and Rac1, members of the Rho small GTPase family, play a vital role in the control of cell migration. Using predictive mathematical modeling, mass spectrometry-based quantitation of network components, and experimental validation in MDA-MB-231 mesenchymal breast cancer cells, we show that a network containing Rac1, RhoA, and PAK family kinases can produce bistable, switch-like responses to a graded PAK inhibition. Using a small chemical inhibitor of PAK, we demonstrate that cellular RhoA and Rac1 activation levels respond in a history-dependent, bistable manner to PAK inhibition. Consequently, we show that downstream signaling, actin dynamics, and cell migration also behave in a bistable fashion, displaying switches and hysteresis in response to PAK inhibition. Our results demonstrate that PAK is a critical component in the Rac1-RhoA inhibitory crosstalk that governs bistable GTPase activity, cell morphology, and cell migration switches.**

## INTRODUCTION

The members of the Rho family of small guanosine triphosphatase (GTPases), RhoA and Rac1, play crucial roles in a range of cellular functions, including the regulation of the actin cytoskeleton, cell polarity and migration, gene expression, and cell proliferation (Jaffe and Hall, 2005; Takai et al., 2001). Rho GTPases function as molecular switches, cycling between inactive guanosine diphosphate (GDP)-bound (“off”) and active GTP-bound (“on”) states. In their “on” state, Rho GTPases can bind downstream effector proteins, initiating signaling through multiple pathways. The GTPase activation-deactivation cycle is tightly controlled by two opposing enzyme groups, (1) guanine exchange factors (GEFs), which facilitate switching from GDP to guanosine triphosphate (GTP), and (2) GTPase-activating proteins (GAPs), which stimulate GTP to GDP hydrolysis.

Active Rho family GTPases, Rac1 and RhoA, induce the membrane translocation of downstream effectors and trigger their activation, which commonly involves post-translational modifications and conformational changes of bound proteins (Bos et al., 2007; Bustelo et al., 2007). Membrane-bound Rac1-GTP recruits p21-activated kinases (PAKs) by binding to their Cdc42-Rac interactive binding (CRIB) domain. In resting cells, type I PAKs are localized in the cytoplasm as inactive dimers, with the regulatory domain shielding the kinase domain. Rac1 binding induces a conformational change and subsequent activation of PAKs, which then can phosphorylate downstream substrates. The PAKs’ activity converts the local activation of Rho-type GTPases into cell-wide responses (Bokoch, 2003; Zhao and Manser, 2012).

Rac1 and RhoA, along with their fellow Rho GTPase family member Cdc42, work in a coordinated fashion to control cell migration (for reviews, see Burridge and Wennerberg, 2004; Parri and Chiarugi, 2010; Ridley et al., 2003). Rac1 is responsible for driving actin polymerization at the leading edge of a migrating cell, resulting in the formation of lamellipodia, which pushes the cell membrane forward (Nobes and Hall, 1995, 1999; Parri and Chiarugi, 2010; Ridley et al., 1992). Rac1 also promotes focal complex assembly (Nobes and Hall, 1995; Parri and Chiarugi, 2010) and is essential for migration (Nobes and Hall, 1999). RhoA is required for cell adhesion (Nobes and Hall, 1999). It stimulates contractility in cells through myosin light-chain (MLC) phosphorylation, which induces the formation of stress fibers and focal adhesions (Chrzanowska-Wodnicka and Burridge,

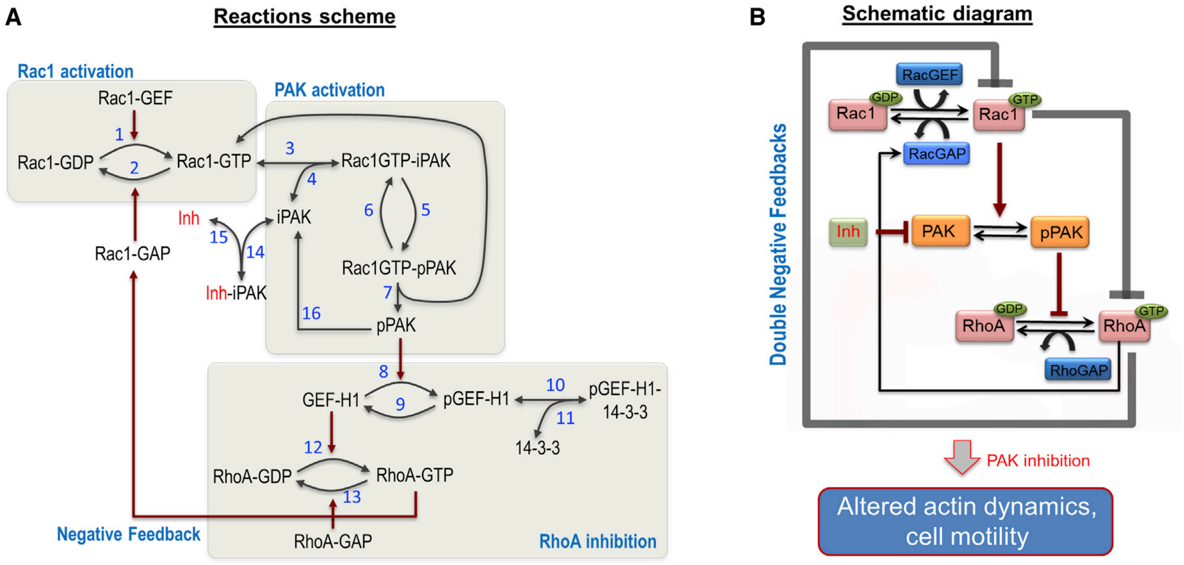


**Figure 1. Reaction Scheme and Schematic Interactions of the Rac1-RhoA Network Model**

(A) Reaction scheme of the kinetic Rac1-RhoA model showing three layers of interconnected regulation of Rac1 and RhoA via PAK (see main text and the Supplemental Information for more details). iPAK and pPAK indicate inactive and active PAK, respectively; we denote PAK Inh as a general but selective PAK inhibitor; and Rac1-GTP, and RhoA-GTP indicate the GTP-bound, active forms of Rac1 and RhoA, respectively.

(B) An abstract-level, schematic diagram of the Rac1-RhoA network shows the flow of signaling and highlights the double-negative feedback regulation between Rac1 and RhoA.

1996; Ridley and Hall, 1992). From the perspective of cell morphology, Rac1 and RhoA oppose each other. Although the picture is likely more complicated (see Discussion), canonical descriptions of cell migration place active Rac1 at the migrating cell's front and active RhoA at its back. Biochemically, Rac1 and RhoA are generally found to interact in mutually antagonistic ways, playing opposing roles in cell migration (Ohta et al., 2006; Sanz-Moreno et al., 2008; reviewed in Guilluy et al., 2011).

Double-negative feedback loops resulting from mutual inhibition can lead to bistability (Kholodenko, 2006). A bistable system can flip between two biochemically distinct steady states; in the proper context, these steady states can promote different cellular phenotypes. Thus, the existence of bistability enables switch-like behaviors in which a graded, analog change in signal inputs could cause abrupt, digital responses in signaling outputs (Ferrell, 2002; Tyson et al., 2003). Bistability has been observed in many biological systems, including the mitogen-activated protein kinase (MAPK) family cascades (Bhalla et al., 2002; Markevich et al., 2004, 2006; Xiong and Ferrell, 2003) and Cdc2 activation circuit (Pomerening et al., 2003; Sha et al., 2003), which play important roles in diverse cellular functions such as development and memory (Ogasawara and Kawato, 2010). Although it was suggested that mutual inhibition between Rac1 and RhoA may result in bistable activity responses (Jilkine et al., 2007; Symons and Segall, 2009; Tsyganov et al., 2012), this behavior and the consequences for cell migration have not yet been experimentally observed. Here, we combine kinetic modeling and experimentation to demonstrate the existence of bistability in the Rac1-RhoA signaling system of highly motile MDA-MB-231 cells. Model analysis and simulations predict that graded changes in PAK activity induce bistable responses of Rac1 and RhoA activities, which are experimentally validated. Furthermore, the bistable properties of the Rac1-RhoA biochemical circuitry are translated into bistability of the actin dynamics and cell migration.

## RESULTS

### Mathematical Modeling of the Rac1-RhoA Interaction Network

To explore the signaling properties of the Rac1-RhoA network, we developed a kinetic model of the network circuitry that captures main molecular events including protein-protein interactions, GTPase activation and deactivation, phosphorylation and dephosphorylation, and feedback regulations. A detailed reaction scheme of the Rac1-RhoA network is given in Figure 1A, while Figure 1B presents a schematic diagram showing the signal flow in the network among Rac1, RhoA, and PAK. The model is formulated as a system of ordinary differential equations using a combination of mass-action and enzyme kinetics laws and is fully described in the Supplemental Information.

Our kinetic model is tailored to MDA-MB-231 cells, where GEF-H1 is one of the dominant RhoA GEFs (Heck et al., 2012; von Thun et al., 2013). The model assumes that Rac1 activates PAK by binding to the PAK CRIB domain, causing a conformational transition of PAK. This exposes PAK's activation loop, which is subsequently auto-phosphorylated through an intramolecular mechanism, resulting in full activation of the kinase (Bokoch, 2003; Zhao and Manser, 2012). Activated PAK phosphorylates GEF-H1, a GEF for RhoA, on inactivating inhibitory sites (Zenke et al., 2004). Following phosphorylation, GEF-H1 binds to 14-3-3 protein, a small, phospho-motif binding, dimeric adaptor protein, which causes the GEF-H1 relocation to microtubules (Zenke et al., 2004), where its activity substantially

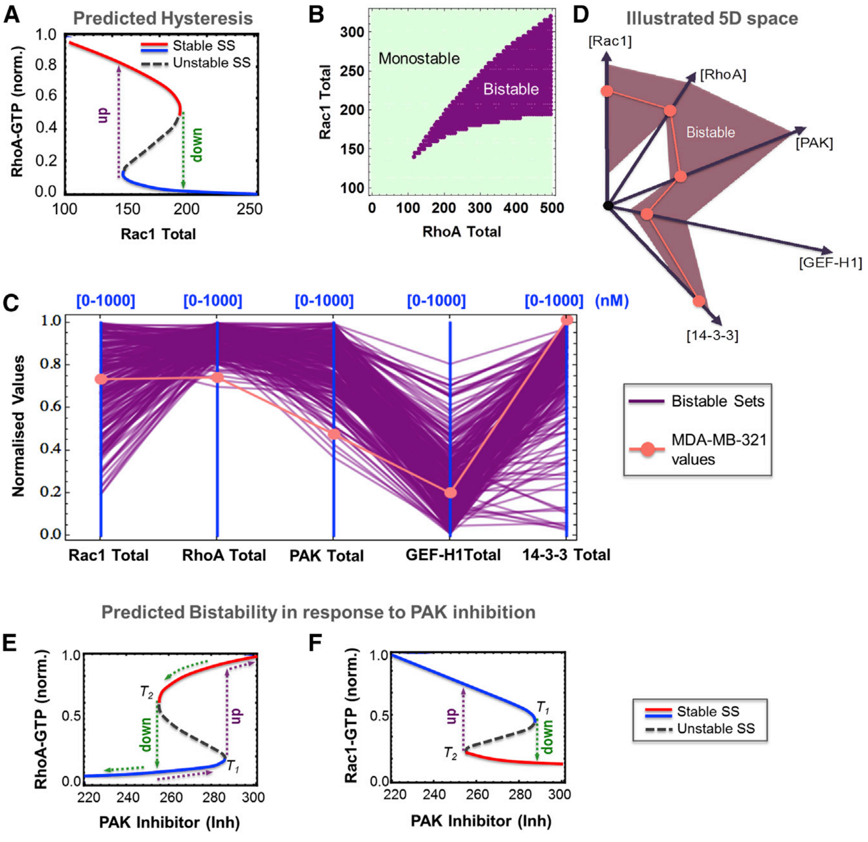


**Figure 2. The Rac1-RhoA Double-Negative Feedback Loop Can Result in Bistable Behavior**

(A) Active RhoA-GTP responses to increasing Rac1 abundance in a bistable manner, resulting in abrupt switches of activity level. SS, Steady State.

(B) Two-parameter (2D) bifurcation diagrams showing dependence of bistability on the abundance of Rac1 and RhoA.

(C) A parallel coordinate plot showing the bistable parameter sets (purple) obtained by assessing the dynamics of 100,000 sets with randomly sampled Rac1, RhoA, PAK, GEF-H1, and 14-3-3 totals within the indicated ranges (in nanomolars). The values are normalized between 0 and 1. A detailed description of the multi-dimensional dynamic analysis and parallel coordinate representation is given in Nguyen et al. (2015) and Supplemental Experimental Procedures.

(D) An intuitive simplified illustration of the bistable region in the corresponding 5D parameter space.

(E and F) Simulated bistability and hysteresis for active Rac1 and RhoA in response to increasing the PAK inhibitor level. All simulations were carried out with parameter values given in Table S4.

decreases (Krendel et al., 2002). In this way, Rac1 inhibits RhoA activity through PAK, seen as the downward negative regulation from Rac1 to RhoA in Figure 1B.

We have experimentally shown that expression of constitutively active RhoA (RhoAV14) in MDA-MB-231 cells decreases the level of active Rac1 (Figure S1). There are several potential routes for the inhibition of Rac1 by RhoA, including through the regulation of RacGAPs, e.g., ARHGAP22 (Sanz-Moreno et al., 2008) and FilGAP (Saito et al., 2012), which are phosphorylated and activated by the Rho effector kinase ROCK. In our model, we assume that active RhoA deactivates Rac1 via activation of Rac1 GAPs (Figure 1A). This constitutes a negative regulation from RhoA to Rac1, as depicted in Figure 1B, closing a double-negative feedback loop between Rac1 and RhoA.

To determine the abundance of the proteins in the network, we quantified the concentration of the proteins by quantitative mass spectrometry. Several mass spectrometry methods have been developed that use the sum of peptide ion current integrals to estimate absolute protein concentrations. Overall, the error of these methods will be below one order of magnitude and will mostly be within a 2-fold window for abundant proteins (Li et al., 2014; Schwanhäusser et al., 2011). Using a combination of filter-aided sample preparation (Wiśniewski et al., 2009) and the proteome ruler approach (Wiśniewski et al., 2014), we quantified the ~5,000 most abundant proteins in MDA-MB-231 cells. PAK2 is the only PAK isoform we identified in this dataset. GEF-H1 and p115-RhoGEF are the most highly expressed RhoA GEFs, and Rac1 and RhoA are the most abundant isoforms among the Rac and Rho family in these cells (Tables S5 and S6). p115-RhoGEF is mostly active downstream of G protein-coupled receptors and was consequently omitted from the model. However, GEF-H1 has been shown to be a major contributor toward RhoA activity in this cell line (von Thun et al., 2013). Our model therefore considers only these Rac, Rho, and PAK isoforms and GEF-H1. This makes the model simple enough for efficient analysis, yet it captures the most essential biological interactions.

## Bistable Switch-like Responses in the Modeled Rac1-RhoA System

To determine MDA-MB-231 cell-specific parameters, we measured the expression levels of key proteins incorporated in the model, including Rac1, RhoA, PAK, GEF-H1, and 14-3-3, by quantitative mass spectrometry (Table S5). After populating the model with these values (see Supplemental Information) and carrying out extensive simulations of steady-state dose responses, bistability of the Rac1-RhoA system was observed in a wide parameter range. In the bistable parameter region, Rac1 and RhoA activity levels responded in a switch-like manner to graded changes in levels of various species. For example, Figure 2A shows a model simulation in which the active RhoA-GTP displays a bistable response to the graded increase in the total Rac1 abundance. We can see that active RhoA levels suddenly jump from a high to a low state when Rac1 gradually increases from an initial low abundance (dashed, green line, Figure 2A), while the backward traverse of Rac1, that is, starting at a high abundance and gradually reducing the total amount of Rac1, abruptly pushes active RhoA from a low to a high state at a different, lower Rac1 threshold (dashed, purple line, Figure 2A). A system's ability to have two quantitatively different thresholds, associated with each of two quantitatively different starting

states, defines the so-called hysteresis phenomenon. Hysteresis is a hallmark of bistable dynamics (Kholodenko, 2006).

Although the ability to support bistable dynamics is a feature of this network's structure, bistable behavior only occurs at certain parameter values. When parameters are changed, bistability can occur or disappear. Such dramatic changes in the system dynamic behavior are called bifurcations. If two parameters are selected, a two-dimensional (2D) plane of these parameters can be conveniently divided into areas where bistability is present or the GTPase network has a single steady state (called monostability). This partitioning of the parameter space, often referred to as 2D bifurcation diagrams, enables one to discern how changes in the abundances of different model species affect the occurrence and existence of bistability, that is, its robustness. Figure 2B displays a 2D bifurcation plot showing the system is bistable over a large region (in purple) of the Rac1 and RhoA abundances at the assumed physiological kinetic parameter values, while the remaining region exhibits monostability (in light green). Next, we investigated the dependence of bistability in more than two dimensions when multiple model parameters are allowed to simultaneously change. This analysis helped us understand the system dynamics in the multi-dimensional parameter space.

To visualize multi-dimensional parameter settings in which bistability is present or absent, we use a software program DYVIPAC (see Supplemental Experimental Procedures and Figure S2, which illustrates the DYVIPAC methodology) (Nguyen et al., 2015). Figure 2C displays the results of multi-dimensional parameter analysis in the form of parallel coordinate plots where the abundances of five model species, Rac1, RhoA, PAK, GEF-H1, and 14-3-3, are allowed to change within physiologically sensible ranges (here, from 0 to 1,000 nM). Systematic sampling of 100,000 sets in this five-dimensional (5D) parameter space for DYVIPAC analysis shows that the system can display either bistable or monostable behavior. For ease of visualization, Figure 2C displays only the bistable sets using the same color, while individually color-coded bistable sets could be seen in Figure S3B alone or with the monostable sets in Figure S3A. Figure 2C indicates that bistability occurs for a large number of parameter sets.

Bistability is found to strongly associate with high RhoA, PAK, and 14-3-3 abundances but low GEF-H1 abundance, while bistability may occur at both high and low Rac1 levels. When we overlay the measured protein levels for MDA-MB-231 cells, we observe that the MDA-MB-231-specific values appear to be within the bistable region (Figure 2C) for the assumed values of kinetic constants. A more intuitive visualization of bistable region in the 5D parameter space is given in Figure 2D. For larger sampling ranges (0 to 5,000 nM) our analysis reveals similar patterns of the bistable region, suggesting that relative rather than absolute values of these species determine the occurrence of bistability (Figure S4). Similar analysis describing the effect of the kinetic parameters on the bistable region (Figures S5 and S6) and the behavior of a comparable dimensionless model (Supplemental Experimental Procedures and Figure S7) demonstrates that this system's bistable region is wide.

Rac1 and RhoA do not act in isolation inside the cell, but through their immediate effectors, PAK and ROCK, these GTPases regulate cytoskeleton dynamics and cell migration. Because PAK activity can be perturbed experimentally, we next asked how Rac1 and RhoA activity would behave in response to PAK inhibition. To this end, we extended our model to include PAK inhibition, which is described as a reaction in which a PAK inhibitor binds to inactive, unphosphorylated PAK, competing with PAK binding to Rac1-GTP (reactions 14 and 15, Figure 1A) (Deacon et al., 2008; Viaud and Peterson, 2009). Model simulations suggest that active RhoA and Rac1 display bistable, switch-on and switch-off responses, respectively, to increasing PAK inhibitor levels (Figures 2E and 2F). Within a defined range of inhibitor concentration, active Rac1 and RhoA achieved one of two stable steady states (solid lines, Figures 2E and 2F) but could not settle in the intermediate, unstable one (dashed lines, Figures 2E and 2F). At low inhibition levels, RhoA-GTP could display a single low steady-state value. As the inhibitor level increases, active RhoA rises slowly until it reaches a threshold level ($T_1$) at which it suddenly switches to a high state (purple dashed lines, Figure 2E). In contrast, when the high active RhoA state is initially induced by the high PAK inhibitor levels and then the inhibitor level decreases, active RhoA slowly decreases until it reaches a second, lower threshold ($T_2$) at which active RhoA drops precipitously and switches to a low state (green dashed line, Figure 2E). Similarly, active Rac1 reacts in a bistable manner to the PAK inhibitor, although in an opposite fashion to RhoA (Figure 2F). A 2D bifurcation analysis further shows that bistability occurs within a bounded range of inhibitor concentration (Figures S8A and S8B). When we vary the levels of the key model species within 2-fold of their measured values in MDA-MB-231, RhoA-GTP and Rac1-GTP continue to exhibit bistable responses to graded PAK inhibition but the switching thresholds are parameter dependent (Figures S8C and S8D). Thus, our model analysis suggests that Rac1 and RhoA activities can display robust bistable responses to PAK inhibition.

## Experimental Validation of Bistable Switches of Rac1 and RhoA Activity Levels

Here, we test model predictions experimentally using the MDA-MB-231 human breast cancer cell line. These experiments rely on chemical inhibition of PAK, because small molecule inhibitors, which are quickly imported and exported from cells, can reach equilibrium within the time frame of the experiments before transcriptional feedback effects take place and the system changes in fundamental ways. We used IPA-3, a chemical inhibitor that specifically targets inactive group 1 PAKs (Deacon et al., 2008; Viaud and Peterson, 2009) by preferentially binding to the inactive conformation of the PAKs' regulatory domain. Accordingly, it has been observed to inhibit Rac1-mediated PAK activation dose dependently, whereas IPA-3 has a substantially reduced effect on already-active PAK (Deacon et al., 2008; Viaud and Peterson, 2009). To determine the half-life of intercellular IPA-3, we treated the cells with IPA-3 and replaced the media after 20 min of incubation. We then washed the cells with PBS, extracted cellular IPA-3 with methanol, and quantified it by mass spectrometry. We found that the half-life was around 2–5 min (Figure S9). After a 10 min washout, the cellular levels had decreased to 10%–25% of the initial concentration, and IPA-3 levels were undistinguishable from an untreated control after 20 min.

To determine whether RhoA and Rac1 behave in a bistable manner in MDA-MB-231 cells, we incubated cells that initially have low RhoA-GTP and high Rac1-GTP levels with different, incrementally increasing concentrations of IPA-3, ranging from 0 to 15 μM, for 40 min, as indicated in Figures 3A–3D. Furthermore, to change the initial RhoA and Rac1 activities, we pretreated cells with the highest concentration of IPA-3 (15 μM) for 20 min, locking the system into a high RhoA (Figures 3A and 3B) and low Rac1 (Figures 3C and 3D) activity state. At this point, the inhibitor was washed off, and then incrementally increasing IPA-3 concentrations from 0 to 15 μM were added for an additional 20 min (Figures 3A–3D; see Figure S10 for a workflow diagram of the experiment). Active, GTP-bound RhoA and Rac1 were precipitated with GST-Rhotekin and GST-PAK-CRIB beads, respectively; detected using western blots; and normalized against total RhoA and Rac1 levels (Figures 3A and 3C, respectively). Densitometric analyses of three replicates are shown in Figures 3B and 3D. The blue curves show the responses of active RhoA and Rac1 to incrementally increasing PAK inhibition, and the red curves show their responses to incrementally decreasing PAK inhibition after being initially locked in the state produced by high PAK inhibition (as predicted by simulations in Figures 2E and 2F). Comparing experimental quantifications with model predictions shows that similar switch-like and hysteretic behaviors are present. Loading controls confirm that expression levels of Rac1 and RhoA remain stable over the course of the experiment, thus excluding the possibility that the observed hysteresis is the consequence of altered protein expression in response to PAK inhibition.

## Bistability of Signaling Downstream of RhoA

Having determined that active Rac1 and RhoA behave in a bistable manner in response to PAK inhibition, we wanted to see whether molecular events downstream of the GTPases behave similarly. RhoA-GTP can bind and activate Rho kinase (ROCK), which in turn inhibits MLC phosphatase, as well as directly phosphorylating MLC (Nobes and Hall, 1999). In addition to phosphorylating MLC via ROCK, active RhoA has been shown to increase the formation of actin stress fibers by enhancing actin nucleation and reducing actin depolymerization (Chesarone and Goode, 2009; Ridley and Hall, 1992). Thus, we decided to determine whether IPA-3 could increase stress fiber formation and phosphorylated MLC (pMLC). F-actin stress fibers and pMLC can be imaged by phalloidin staining and by immunofluorescence, respectively. Both readouts can be quantified by high-content microscopy. In addition to quantifying changes at the population level, this approach would allow us to determine F-actin and pMLC at the single-cell level. These data would reveal how the distribution of either marker of RhoA activity was changed in response to PAK inhibition within the cell population.

We seeded MDA-MB-231 cells in collagen-coated 96-well plates and treated the cells with increasing concentrations of IPA-3 for 80 min (non-pretreated). Alternatively, we pretreated the cells with a high concentration of the inhibitor for 20 min, followed by a washout and incubation with increasing concentrations of IPA-3 for an additional 60 min (see Figure S10 for workflow). After the treatment was completed, we fixed, permeabilized, and stained the cell body, F-actin, nuclei, and pMLC with a high-content screening cell mask, phalloidin, Hoechst, and specific primary and fluorescent secondary antibodies (Figure 3E). We imaged the cells on a high-content automated microscope and processed the acquired images to isolate individual cells and further segment the cells into nucleus, cell body, and lamellipodium (Figure S11A). When we plotted the median intensity of cellular lamellipodial pMLC, we observed hysteresis (Figure S11B). In non-pretreated cells, IPA-3 increased pMLC intensity at the highest concentrations, whereas when cells were pretreated, we observed that the switch occurred at the lowest concentrations (Figure S11B). We then plotted single-cell lamellipodial pMLC levels in a histogram to observe whether IPA-3 levels altered the distribution of pMLC in the cell populations. In MDA-MB-231 without any IPA-3 treatment, pMLC was spread in a long-tailed distribution, possibly already containing two populations with distinct population averages (Figure 3F). This distribution was unaltered for the low and medium concentrations of the inhibitor, but we observed a broadening at the highest IPA-3 concentration (Figure 3F). In cells that were pretreated with IPA-3, we observed that the broadening of the pMLC distribution already occurred at low inhibitor concentrations (Figure 3G). The broadening of the distributions suggests that two, possibly three, cell populations with distinct average pMLC levels can be present under these conditions. This potentially multi-modal distribution suggests that pMLC levels may be determined by more complex signaling, which is not captured in its entirety by our model.

We then quantified cytoplasmatic F-actin in the same sample set. Analogous to what we observed with pMLC, only higher concentration of IPA-3 increased cellular F-actin (Figure S11C). In contrast, pretreating the cells increased the cellular quantity of F-actin across all IPA-3 concentrations (Figure S11C). We then plotted the F-actin single-cell distribution and observed that most non-pretreated MDA-MB-231 cells had no or low amounts of stress fibers at low or medium inhibitor concentrations. Incubation with 7.5 μM IPA-3 gave rise to a second population that was positive for F-actin (Figures 3H and 3I). Pretreatment with IPA-3 for 20 min was also sufficient to increase the presence of this second population (Figure 3J). Washing IPA-3 completely off reduced the levels of the stress fiber-containing cells but not to the levels of the initial condition, suggesting that 20 min of IPA-3 induced actin stress fibers irreversibly within the time frame of the experiment. This is possibly due to the stability of the stress fibers, and 1 hr may not be enough to efficiently disassemble them. In summary, these data showed that both pMLC and F-actin are induced by IPA-3. Instructively, we observed hysteresis for both markers of RhoA activity. Furthermore, our data showed that F-actin is bimodally distributed, and there is a likelihood that this was the case for pMLC.

## Bistable Behavior of Actin Dynamics

Given the requirement of Rac1 and RhoA interactions for actin assembly and disassembly (Burridge and Wennerberg, 2004; Nobes and Hall, 1995; Ridley and Hall, 1992) and in initiating protrusions at the leading edge (Machacek et al., 2009; Pertz et al., 2006), we speculated that IPA-3 would also regulate actin dynamics in a bistable fashion, a hypothesis we tested next.

Live-cell actin polymerization dynamics can be visualized by expressing fluorescent proteins tagged to an F-actin binding peptide (LifeAct) (Riedl et al., 2008). Therefore, we generated

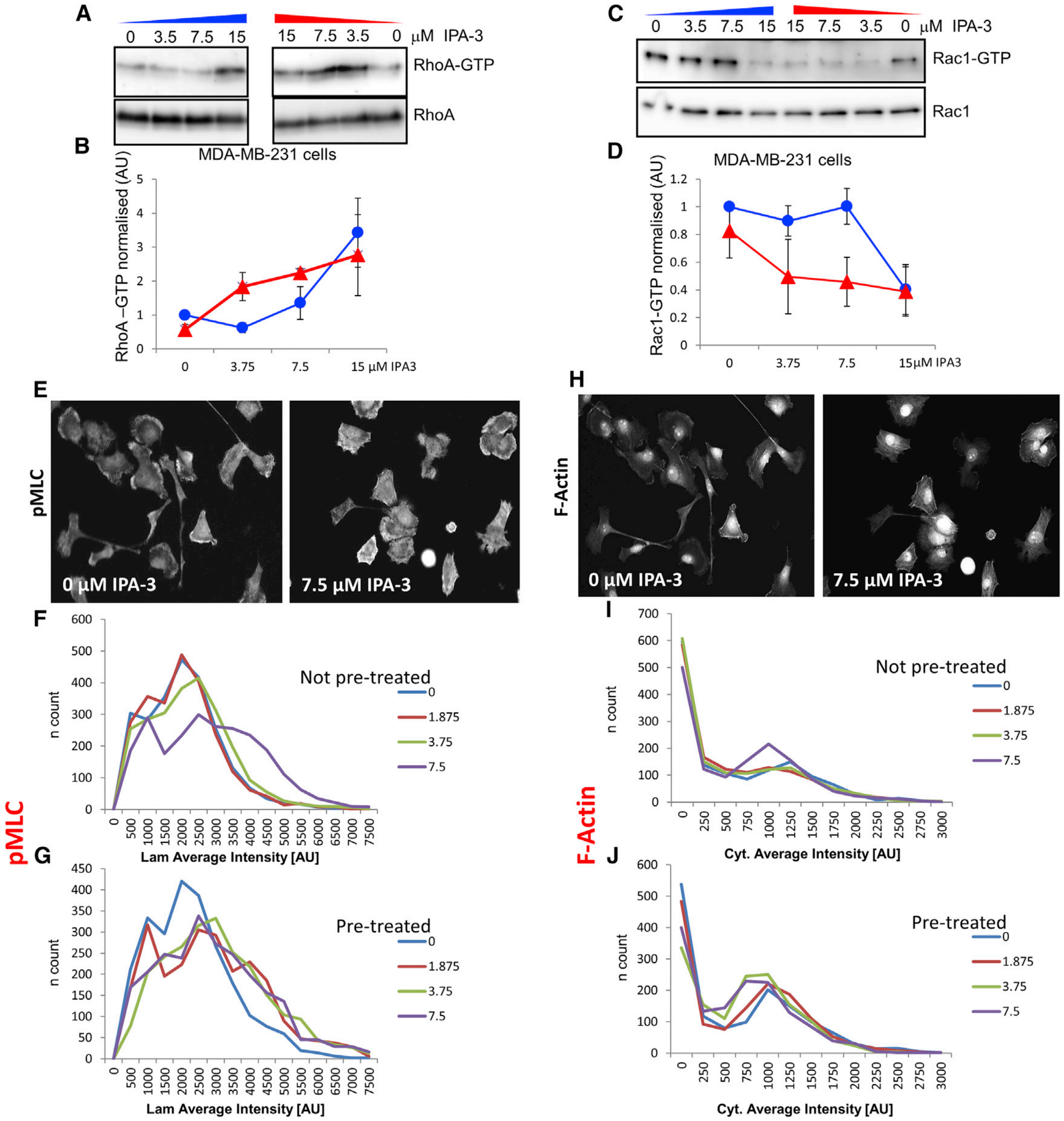


**Figure 3. Experimental Validation of RhoA and Rac1 Bistable Switches in MDA-MB-231 Cells**

(A–D) PAK was inhibited in MDA-MB-231 cells by adding specific inhibitor IPA-3 at the indicated concentrations for 40 min (blue) or by incubating MDA-MB-231 cells with 15 μM IPA-3 for 20 min. The inhibitor was subsequently washed out, and the cells were incubated for an additional 20 min with IPA-3 at the indicated concentrations (red). (A) RhoA-GTP was precipitated with GST-Rhotekin beads and western blotted. (B) Densitometric analysis of three biological replicates. Error bars represent SD. (C) Rac1-GTP was precipitated with GST-PAK-CRIB beads and western blotted. (D) Densitometric analysis of three biological replicates. Error bars represent SD.

(E) MDA-MB-231 cells seeded on collagen were treated for 80 min with the indicated concentrations of IPA-3, fixed and stained with an anti-pS19 MLC2 antibody. 20× image.

(F) MDA-MB-231 cells seeded on collagen were treated for 80 min with the indicated concentrations of IPA-3, fixed and stained with an anti-pS19 MLC2 antibody. Histogram of single-cell, averaged lamellipodial intensity.

(G) MDA-MB-231 cells seeded on collagen were treated for 20 min with 7.5 μM IPA-3. The cells were subsequently washed and treated for an additional 60 min with the indicated concentrations of IPA-3, fixed and stained with an anti-pS19 MLC2 antibody. Histogram of single-cell, averaged lamellipodial intensity.

(H) MDA-MB-231 cells seeded on collagen were treated for 80 min with the indicated concentrations of IPA-3, fixed and stained with fluorescent phalloidin. 20× image.

(I) MDA-MB-231 cells seeded on collagen were treated for 80 min with the indicated concentrations of IPA-3, fixed and stained with fluorescent phalloidin. Histogram of single-cell, averaged cytoplasmatic intensity.

(J) MDA-MB-231 cells seeded on collagen were treated for 20 min with 7.5 μM IPA-2. The cells were subsequently washed and treated for an additional 60 min with the indicated concentrations of IPA-3, fixed and stained with fluorescent phalloidin. Histogram of single-cell, averaged cytoplasmatic intensity.

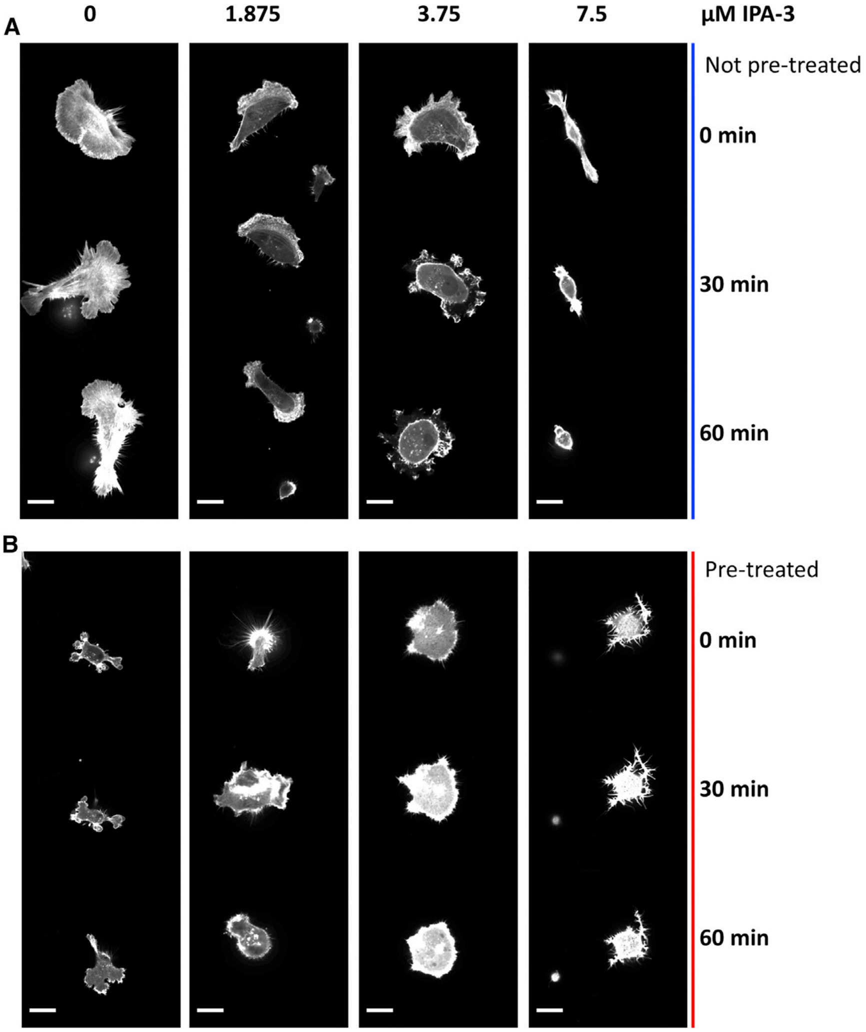


**Figure 4. Actin Dynamics Behave in a Bistable Manner in Response to PAK Inhibition**

Sparsely seeded MDA-MB-231 cells expressing a LifeAct-mCherry probe were treated with the PAK inhibitor IPA-3 (A) at the indicated concentrations for 60 min (blue) or (B) by incubating cells with 7.5 μM IPA-3 for 20 min. The inhibitor was subsequently washed out, and the cells were incubated for an additional 60 min with IPA-3 at the indicated concentrations (red). Images were taken every 15 s. Montage images represent changes in actin dynamics during the cells' migration, showing three images (0, 30, and 60 min) over the 1 hr period. Scale bar, 20 μM.

MDA-MB-231 cells that stably express mCherry-tagged LifeAct. Similar to earlier, we exposed cells in their normal state to incrementally increasing concentrations of IPA-3. Alternatively, we first pretreated cells with high IPA-3 doses for 20 min, thereby inhibiting PAKs, and then washed out the inhibitor and again incubated cells with different IPA-3 concentrations. In both cases, we imaged the actin dynamics over 60 min (see Figure S10 for workflow). Untreated control cells exhibited a highly dynamic F-actin cytoskeleton, which rapidly pushed and retracted cellular protrusions and lamellipodia (Figure 4A; Movie S1). Low concentrations of IPA-3 (1.875 and 3.75 μM) did not affect the dynamic behavior, whereas high concentrations (7.5 μM) initially froze the actin polymerization (Figure 4A; Movies S2, S3, and S4). Subsequently, some cells contracted and membrane blebs appeared (Movie S4), which is indicative of high RhoA activity. In isolated cases, the cells even detached from the glass surface. Equally, pretreatment of the cells with high concentrations of IPA-3 resulted in stalling actin dynamics, blebbing, and a contracted phenotype (Figure 4B; Movies S5, S6, S7, and S8). When the IPA-3 concentration was dropped below 3.75 μM, actin dynamics recovered, blebbing was reduced, and the cells started spreading and initiating lamellipodia (Figure 4B; Movies S5 and S6). In contrast, cells in which the IPA-3 concentration was not altered or reduced to 3.75 μM did not recover and remained in a frozen state (Figure 4B; Movie S7). Taken together, these data show that analogous to RhoA- and Rac1-GTP, actin dynamics respond in a bistable manner when perturbed by PAK inhibition.

## Bistable Behavior of Cell Migration

Actin-driven cellular protrusions and retrograde flow are essential for efficient cell migration. It has been previously demonstrated that Rac activity is required for cell migration and that global inhibition of Rac arrests cell migration (Nobes and Hall, 1999). Therefore, based on our observation that IPA-3 affects actin dynamics and Rac1 activity levels in a bistable manner, we hypothesized that cell migration should behave analogously. To test this hypothesis, we next measured how migration of MDA-MB-231 cells was affected by PAK inhibition, performing random and directed migration assays. Random migration is the expression of the intrinsic cell directionality in the absence of any external guiding factor, whereas directed migration requires steering by an external guidance cue (Petrie et al., 2009). In both cases, the interplay and localization of Rac1 and RhoA activity determine migration speed and directionality.

To determine random migration speed, cells were seeded at low confluency and treated with incrementally increasing concentrations of IPA-3 ranging from 0 to 7.5 μM. An additional set of cells was treated with the highest concentration of IPA-3, 7.5 μM for 20 min. IPA-3 was then washed off and replaced with incrementally increasing inhibitor concentrations from 0 to 7.5 μM IPA-3, as indicated in Figure 5A. The IPA-3 concentrations are slightly different from those used in Figures 3A–3D due to batch-to-batch variability. Preliminary migration experiments done using IPA-3 from the first batch showed that full migration inhibition required 15 μM IPA-3 (Figure S12A). We took transmission-light images every 20 min for 12 hr, and the path of migration of individual cells was manually tracked (see Figure S10 for workflow). The migration paths of individual cells

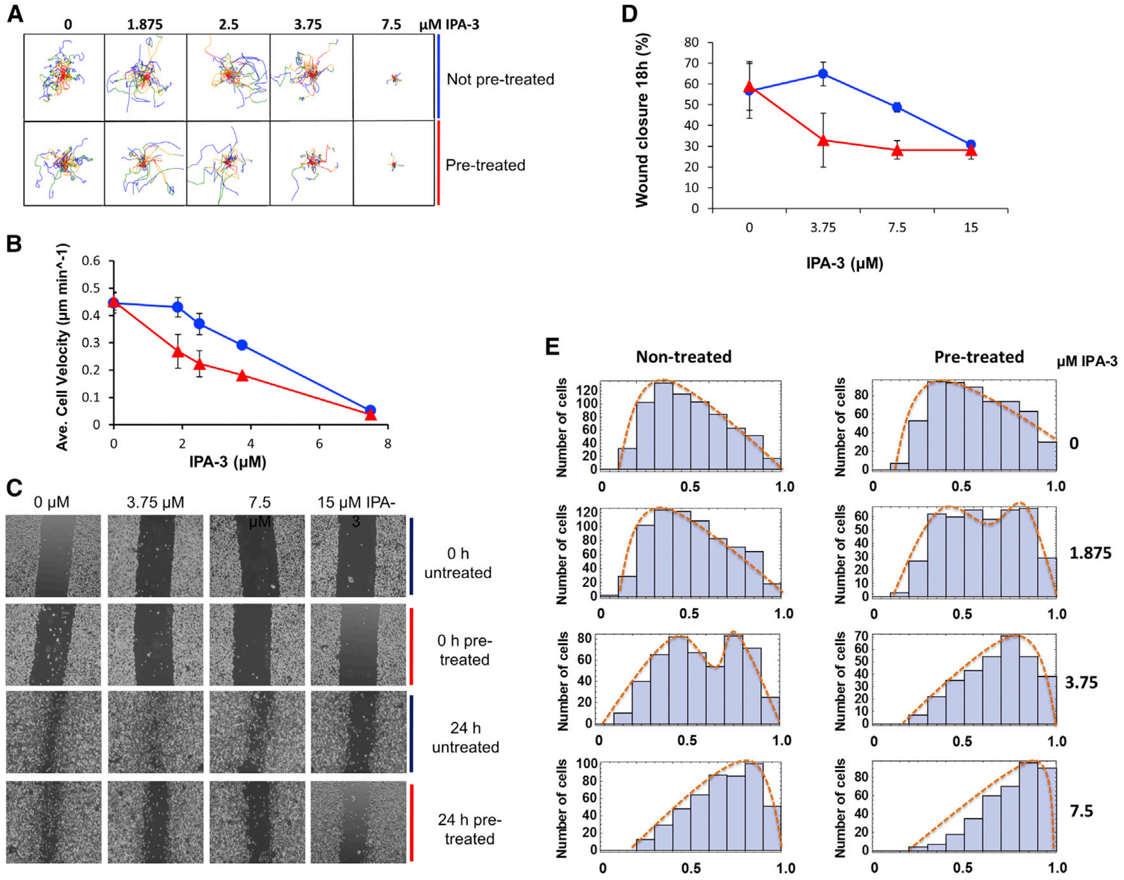


**Figure 5. Migration of MDA-MB-231 Cells Is Regulated in a Bistable Manner by PAK**

(A) Undirected cell migration. Sparsely seeded MDA-MB-231 cells were treated with the PAK inhibitor IPA-3 at the indicated concentrations for 40 min (blue) or by incubating MDA-MB-231 cells with 7.5 μM IPA-3 for 20 min. The inhibitor was subsequently washed out, and the cells were incubated for an additional 20 min with IPA-3 at the indicated concentrations (red). Images were taken every 20 min, and random cell migration of 25 cells was subsequently manually tracked using ImageJ. Each line represents a single cell's migration in one field of view over 12 hr. The line color shows the distance migrated from 0–3 hr (red), 3–6 hr (orange), 6–9 hr (green), and 9–12 hr (blue). Line origins have been artificially placed at (0,0) for display purposes, but lengths remain unchanged. The width and height of each plot is 200 μm.

(B) The mean speed ± SD of three single-cell tracking biological replicates over 12 hr, a representative example of which is found in Figure 5A. The differences between pretreated and non-pretreated 1.185, 2.5, and 3.75 μM IPA-3 were all statistically significant, with p = 0.017, 0.015, and 0.0001, respectively, measured using a two-tailed, non-paired t test. Error bars represent SEM.

(C) Directed cell migration. Confluent MDA-MB-231 cells were scratched with a plastic tip. IPA-3 was added at the indicated concentrations for the duration of the experiment (blue) or by incubating MDA-MB-231 cells with 15 μM IPA-3 for 20 min. The inhibitor was subsequently washed out, and the cells were incubated for the remainder of the experiment with IPA-3 at the indicated concentrations (red). Images of the wound were taken at 0 and 18 hr.

(D) Graph representing the percentage of wound closure of three biological replicates. Error bars represent SD.

(E) MDA-MB-231 cells were sparsely seeded on a thick collagen layer. After 24 hr, they were treated with the PAK inhibitor IPA-3 at the indicated concentrations (non-treated) or by incubating MDA-MB-231 cells with 7.5 μM IPA-3 for 20 min. The inhibitor was subsequently washed out, and the cells were incubated with IPA-3 at the indicated concentrations (pretreated). Images were taken 24 hr later. Histogram represents cell number over cell roundness, with 0 as an infinite line and 1 as a perfect circle.

in a representative replicate are plotted in Figure 5A to give a visual representation of migration. The average speed of each cell was calculated and plotted against PAK inhibition (Figure S12). The quantification in Figure 5B shows that the motility of non-pretreated cells was unaffected at 2.5 μM IPA-3 but subsequently decreased in response to linearly increasing IPA-3 concentrations. Furthermore, the transition from high to low and then low to high migration speeds occurs at different threshold levels of IPA-3, indicating hysteresis. Together, these data suggest that migration of the cells is affected by PAK inhibition in a bistable manner.

Examining instantaneous cell velocities, we found that bistability persists for the duration of the experiment (Figures S12A and S12B). Bistable switches in migration velocity occur in individual cells. Consequently, the change in the population cell velocity average is caused by switch-like velocity changes in different cells rather than by gradual changes in the whole population. Therefore, we hypothesized that under conditions in which the system is bistable (1.865, 2.5, and 3.75 μM IPA-3), the velocities of cells at a given time step (instantaneous velocities) would follow a bimodal distribution, which is a hallmark of a bistable system (Birtwistle et al., 2012; Dobrzyński et al.,

2014). Analysis of individual cell tracks from Figure 5A showed that this was the case (Figure S12C).

We also tested the effect of PAK inhibition on directed cell migration using a wound-healing assay. The cells were treated as before and were photographed immediately after treatment and again after 18 hr (see Figure S10 for workflow). A representative experiment is shown in Figure 5C. A quantification of three replicates is shown in Figure 5D. The results show switch-like inhibition of cell motility in response to graded PAK inhibition. In addition, the switch occurs at different thresholds depending on the initial condition of the systems, indicating the presence of hysteresis (Figures 5C and 5D). These results suggest that directed migration features a bistable response to PAK inhibition.

Finally, to show that the results produced by inhibition of PAK by IPA-3 were not due to an unspecific off-target inhibitor effect, we reproduced some of these results using an unrelated PAK inhibitor ($PAK^i$). $PAK^i$, a genetically encoded PAK1-3 specific inhibitor consisting of a truncated PAK1 regulatory domain, includes the kinase-binding segment that can bind to and inhibit the catalytic domain of PAK1-3 but does not have an intact CRIB domain and therefore cannot bind Rac1 directly (Volinsky et al., 2006). Expression of GFP-coupled PAK ($PAK^i$) or an inactive mutant ($PAK^i$-2 m) was induced by transient transfection in MDA-MB-231 cells. This alternative PAK inhibitor also inhibited Rac1 activity and migration (Figure S13), whereas the inactive mutant had no effect on either. These results confirm that these observed effects are specific to PAK inhibition.

### Bistability and Bimodality of Cell Morphology in 3D Matrices

Cells embedded in three-dimensional (3D) matrices have been shown to invade into the surrounding area by two fundamentally disparate modes of migration (Friedl and Alexander, 2011): mesenchymal and amoeboid. Mesenchymal migration requires cells to proteolytically degrade the matrix through the secretion of matrix metalloproteases (MMPs). This migration mode requires high Rac1-GTP, and it is independent of RhoA activity. Amoeboid migration, however, is independent of MMPs but requires RhoA activity. This migration type is driven by actinomyosin contractility and characterized by a rounded morphology with high RhoA activity.

MDA-MB-231 cells have predominantly elongated morphology in 3D matrices but switch to a rounded cell shape upon RhoA activation or MMP inhibition (von Thun et al., 2013). Thus, we hypothesized that RhoA-GTP elevated by IPA-3 treatment may also alter the morphology of MDA-MB-231 cells. This transition between two morphologies is driven by the RhoA activity changes. Thus, we would expect that, analogous to what we have seen at the molecular level, this switch would be bistable.

To test whether this was the case, we seeded MDA-MB-231 cells on a thick layer of collagen and allowed them to invade the collagen gel over 24 hr. We then incubated the cells either with a range of IPA-3 concentrations or with a 20 min burst of 7.5 μM IPA-3, which was replaced by a set of IPA-3 dilutions. We then imaged the cells embedded in the collagen matrix and determined their shape by automated image analysis (see Figure S10 for workflow). As expected, MDA-MB-231 cells adopted an elongated phenotype in a collagen matrix (quantified in Figure 5E). The 7.5 μM IPA-3 shifted most of the population toward a rounded phenotype. At the intermediate concentration of 3.75 μM IPA-3, we could observe that the distribution of the roundness within the cell population was bimodal. Pretreatment with IPA-3 followed by a washout shifted the switch concentration. In this case, we observed bimodality at the lower concentration of 1.875 μM IPA-3, whereas the cells were predominantly rounded at 3.75 and 7.5 μM IPA-3 (Figure 5E). Taken together, these data showed that the change in morphology is likely to be bistable, because we observed that the IPA-3 concentration needed to induce the switch depends on the initial condition. In addition, we observed bimodality, a general feature of bistable systems.

## DISCUSSION

Rac1 and RhoA are embedded in a wider network of interactions containing extra positive feedback regulations, which were not included in our models. For example, a positive feedback between Rac1 and PAK is formed via the protein Cool-2 (cloned out of library-2) (Baird et al., 2005; Feng et al., 2002), and Rac1 can positively regulate itself in a PAK-independent manner (Tsyganov et al., 2012). However, adding these feedbacks to the system with existing double-negative feedback, or incorporating GDP dissociation inhibitors in the model, only enlarges the bistability range; it did not significantly affect the network behavior (Figure S14) (Nikonova et al., 2013).

For cells to migrate effectively, there must be a highly coordinated interplay between protrusion extension and both adhesion formation and rear end retraction driven by high Rac1 and high RhoA activities, respectively. The high activity of one GTPase requires the low activity of the other to ensure that there is no conflict in the organization of the actin cytoskeleton. The traditional view of Rac1 and RhoA activity in migrating cells was of high Rac1 activity only at the leading edge and high RhoA activity only at the trailing edge. This hypothesis of wide spatial segregation of Rac1 and RhoA activities has since been challenged by localization experiments that describe RhoA activity at the leading edge of migrating cells (Machacek et al., 2009; Pertz et al., 2006) and Rac1 activity at the tail (Gardiner et al., 2002). Though there seems to be a lack of consensus over the exact localizations of active RhoA and Rac1 during the cell movement, and we are likely only seeing a small part of a larger, more complex picture (Wang et al., 2013), a unifying motif is that the activation zones of Rac1 and RhoA are mutually exclusive, either spatially or temporally (Guilluy et al., 2011; Pertz, 2010). This exclusivity found in the spatial and temporal localization of Rac1 and RhoA has been proposed to arise from the bistability of the system (Jilkine et al., 2007; Semplice et al., 2012). Bistability allows the cell (or area of a cell) to make a discrete digital decision in the face of a range of conflicting external signal gradients and ensures that the decision made by the cell is fairly robust. In that way, the cell is responsive to its surroundings but not so sensitive that it is unable to make efficient progress.

Due to the hysteresis present in the system, once PAK is inhibited, it remains locked in that state even at lower inhibitor concentrations. Such circuitries with bistable and hysteretic response characteristics could make attractive targets for

therapy approaches using dose variation, because an initial high dose would switch the system to the inhibited state, which can be maintained using lower drug concentrations. This behavior is desirable for minimizing drug toxicity and in terms of pharmacokinetics ensuring a long-lasting inhibition even when the drug concentrations decline. Our cell motility experiments show that undirected and directed migration are arrested in two dimensions by strong inhibition of PAK (e.g., Figure 4A). This indicates that PAK could be an attractive drug target for blocking the migration of cancer cells. Although IPA-3 is not suitable for clinical use (Zhao and Manser, 2010), our results are valid for various mechanisms of PAK inhibition, and this may stimulate the development of therapeutically appropriate PAK inhibitors in the future.

## EXPERIMENTAL PROCEDURES

For a detailed description of the models and model analyses, see Supplemental Information.

### Cell Culture and Reagents

MDA-MB-231 cells were cultured using standard techniques. The plasmids, reagents, and antibodies used in this study are listed in Supplemental Information.

### Liquid Chromatography-Tandem Mass Spectrometry, Protein Identification and Quantitation, and IPA-3 Quantitation

Samples were analyzed on a Q Exactive mass spectrometer (Thermo Scientific) coupled to ultra-high-performance liquid chromatography as described (Farrell et al., 2014). Identification and quantitation was performed using the MaxQuant/Perseus software suite for proteins or Xcalibur for IPA-3.

### Immunofluorescence

Standard protocols were followed. Images were acquired in an unsupervised manner on an ImageXpress Micro wide-field microscope and were analyzed using the MetaXpress Custom Module Editor (Molecular Devices).

### Live-Cell Imaging

Standard methods were employed. MDA-MB-231 or a clone stably expressing LifeAct-mCherry was seeded in an optical-bottomed plate. Images were acquired on a spinning-disc laser confocal Nikon microscope, an Incucyte ZOOM system, or a Zeiss Axiovert 200M.

### Mathematical Modeling

Mathematical modeling was implemented in Mathematica v.8.0.1.0 (Wolfram Research, 2010). Stability analysis was carried out using XPPaut and Mathematica. Multi-dimensional dynamic analysis and visualization were conducted using DYVIPAC (Nguyen et al., 2015). Bimodal data analysis and plotting was conducted using the R package ggplot2 (Wickham, 2009).

## SUPPLEMENTAL INFORMATION

Supplemental Information includes Supplemental Experimental Procedures, 14 figures, 6 tables, and 8 movies and can be found with this article online at http://dx.doi.org/10.1016/j.cels.2016.01.003.

## AUTHOR CONTRIBUTIONS

Design of Project, A.v.K., L.K.N., M.R.B., and B.N.K.; Biochemical and Cell Biological Assays, A.v.K., K.M.B., N.V., K.K., N.M., J.C.D., J.-C.B.-W., A.J.F., and N.O.C.; Model Development and Analysis, K.M.B., L.K.N., B.N.K., M.A.T., M.D., A.K., and A.D.; Manuscript Writing, W.K., K.M.B., A.v.K., L.K.N., and B.N.K. A.v.K., L.K.N., and B.N.K. contributed equally to this work.

## ACKNOWLEDGMENTS

This work was supported by the EU Seventh Framework under projects PRIMES (Grant No. FP7-278568), SynSignal (Grant No. 613879), and Breast Cancer NOW (2013NovPR183). We would like to thank Olivier Pertz (Basel) for the LifeAct, Mike Olson (Beatson) for the GST-Rhotekin, and Piero Crespo (Santander) for GST-PAK-CRIB constructs.

Received: July 6, 2015
Revised: November 30, 2015
Accepted: January 5, 2016
Published: January 27, 2016

## REFERENCES

Baird, D., Feng, Q., and Cerione, R.A. (2005). The Cool-2/alpha-Pix protein mediates a Cdc42-Rac signaling cascade. Curr. Biol. *15*, 1–10.

Bhalla, U.S., Ram, P.T., and Iyengar, R. (2002). MAP kinase phosphatase as a locus of flexibility in a mitogen-activated protein kinase signaling network. Science *297*, 1018–1023.

Birtwistle, M.R., Rauch, J., Kiyatkin, A., Aksamitiene, E., Dobrzyński, M., Hoek, J.B., Kolch, W., Ogunnaike, B.A., and Kholodenko, B.N. (2012). Emergence of bimodal cell population responses from the interplay between analog single-cell signaling and protein expression noise. BMC Syst. Biol. *6*, 109.

Bokoch, G.M. (2003). Biology of the p21-activated kinases. Annu. Rev. Biochem. *72*, 743–781.

Bos, J.L., Rehmann, H., and Wittinghofer, A. (2007). GEFs and GAPs: critical elements in the control of small G proteins. Cell *129*, 865–877.

Burridge, K., and Wennerberg, K. (2004). Rho and Rac take center stage. Cell *116*, 167–179.

Bustelo, X.R., Sauzeau, V., and Berenjeno, I.M. (2007). GTP-binding proteins of the Rho/Rac family: regulation, effectors and functions in vivo. BioEssays *29*, 356–370.

Chesarone, M.A., and Goode, B.L. (2009). Actin nucleation and elongation factors: mechanisms and interplay. Curr. Opin. Cell Biol. *21*, 28–37.

Chrzanowska-Wodnicka, M., and Burridge, K. (1996). Rho-stimulated contractility drives the formation of stress fibers and focal adhesions. J. Cell Biol. *133*, 1403–1415.

Deacon, S.W., Beeser, A., Fukui, J.A., Rennefahrt, U.E., Myers, C., Chernoff, J., and Peterson, J.R. (2008). An isoform-selective, small-molecule inhibitor targets the autoregulatory mechanism of p21-activated kinase. Chem. Biol. *15*, 322–331.

Dobrzyński, M., Nguyen, L.K., Birtwistle, M.R., von Kriegsheim, A., Blanco Fernández, A., Cheong, A., Kolch, W., and Kholodenko, B.N. (2014). Nonlinear signalling networks and cell-to-cell variability transform external signals into broadly distributed or bimodal responses. J. R. Soc. Interface *11*, 20140383.

Farrell, J., Kelly, C., Rauch, J., Kida, K., García-Muñoz, A., Monsefi, N., Turriziani, B., Doherty, C., Mehta, J.P., Matallanas, D., et al. (2014). HGF induces epithelial-to-mesenchymal transition by modulating the mammalian hippo/MST2 and ISG15 pathways. J. Proteome Res. *13*, 2874–2886.

Feng, Q., Albeck, J.G., Cerione, R.A., and Yang, W. (2002). Regulation of the Cool/Pix proteins: key binding partners of the Cdc42/Rac targets, the p21-activated kinases. J. Biol. Chem. *277*, 5644–5650.

Ferrell, J.E., Jr. (2002). Self-perpetuating states in signal transduction: positive feedback, double-negative feedback and bistability. Curr. Opin. Cell Biol. *14*, 140–148.

Friedl, P., and Alexander, S. (2011). Cancer invasion and the microenvironment: plasticity and reciprocity. Cell *147*, 992–1009.

Gardiner, E.M., Pestonjamasp, K.N., Bohl, B.P., Chamberlain, C., Hahn, K.M., and Bokoch, G.M. (2002). Spatial and temporal analysis of Rac activation during live neutrophil chemotaxis. Curr. Biol. *12*, 2029–2034.

Guilluy, C., Garcia-Mata, R., and Burridge, K. (2011). Rho protein crosstalk: another social network? Trends Cell Biol. *21*, 718–726.

Heck, J.N., Ponik, S.M., Garcia-Mendoza, M.G., Pehlke, C.A., Inman, D.R., Eliceiri, K.W., and Keely, P.J. (2012). Microtubules regulate GEF-H1 in response to extracellular matrix stiffness. Mol. Biol. Cell *23*, 2583–2592.

Jaffe, A.B., and Hall, A. (2005). Rho GTPases: biochemistry and biology. Annu. Rev. Cell Dev. Biol. *21*, 247–269.

Jilkine, A., Marée, A.F., and Edelstein-Keshet, L. (2007). Mathematical model for spatial segregation of the Rho-family GTPases based on inhibitory crosstalk. Bull. Math. Biol. *69*, 1943–1978.

Kholodenko, B.N. (2006). Cell-signalling dynamics in time and space. Nat. Rev. Mol. Cell Biol. *7*, 165–176.

Krendel, M., Zenke, F.T., and Bokoch, G.M. (2002). Nucleotide exchange factor GEF-H1 mediates cross-talk between microtubules and the actin cytoskeleton. Nat. Cell Biol. *4*, 294–301.

Li, J.J., Bickel, P.J., and Biggin, M.D. (2014). System wide analyses have underestimated protein abundances and the importance of transcription in mammals. PeerJ *2*, e270.

Machacek, M., Hodgson, L., Welch, C., Elliott, H., Pertz, O., Nalbant, P., Abell, A., Johnson, G.L., Hahn, K.M., and Danuser, G. (2009). Coordination of Rho GTPase activities during cell protrusion. Nature *461*, 99–103.

Markevich, N.I., Hoek, J.B., and Kholodenko, B.N. (2004). Signaling switches and bistability arising from multisite phosphorylation in protein kinase cascades. J. Cell Biol. *164*, 353–359.

Markevich, N.I., Tsyganov, M.A., Hoek, J.B., and Kholodenko, B.N. (2006). Long-range signaling by phosphoprotein waves arising from bistability in protein kinase cascades. Mol. Syst. Biol. *2*, 61.

Nguyen, L.K., Degasperi, A., Cotter, P., and Kholodenko, B.N. (2015). DYVIPAC: an integrated analysis and visualisation framework to probe multi-dimensional biological networks. Sci. Rep. *5*, 12569.

Nikonova, E., Tsyganov, M.A., Kolch, W., Fey, D., and Kholodenko, B.N. (2013). Control of the G-protein cascade dynamics by GDP dissociation inhibitors. Mol. Biosyst. *9*, 2454–2462.

Nobes, C.D., and Hall, A. (1995). Rho, rac, and cdc42 GTPases regulate the assembly of multimolecular focal complexes associated with actin stress fibers, lamellipodia, and filopodia. Cell *81*, 53–62.

Nobes, C.D., and Hall, A. (1999). Rho GTPases control polarity, protrusion, and adhesion during cell movement. J. Cell Biol. *144*, 1235–1244.

Ogasawara, H., and Kawato, M. (2010). The protein kinase Mζ network as a bistable switch to store neuronal memory. BMC Syst. Biol. *4*, 181.

Ohta, Y., Hartwig, J.H., and Stossel, T.P. (2006). FilGAP, a Rho- and ROCK-regulated GAP for Rac binds filamin A to control actin remodelling. Nat. Cell Biol. *8*, 803–814.

Parri, M., and Chiarugi, P. (2010). Rac and Rho GTPases in cancer cell motility control. Cell Commun. Signal. *8*, 23.

Pertz, O. (2010). Spatio-temporal Rho GTPase signaling—where are we now? J. Cell Sci. *123*, 1841–1850.

Pertz, O., Hodgson, L., Klemke, R.L., and Hahn, K.M. (2006). Spatiotemporal dynamics of RhoA activity in migrating cells. Nature *440*, 1069–1072.

Petrie, R.J., Doyle, A.D., and Yamada, K.M. (2009). Random versus directionally persistent cell migration. Nat. Rev. Mol. Cell Biol. *10*, 538–549.

Pomerening, J.R., Sontag, E.D., and Ferrell, J.E., Jr. (2003). Building a cell cycle oscillator: hysteresis and bistability in the activation of Cdc2. Nat. Cell Biol. *5*, 346–351.

Ridley, A.J., and Hall, A. (1992). The small GTP-binding protein rho regulates the assembly of focal adhesions and actin stress fibers in response to growth factors. Cell *70*, 389–399.

Ridley, A.J., Paterson, H.F., Johnston, C.L., Diekmann, D., and Hall, A. (1992). The small GTP-binding protein rac regulates growth factor-induced membrane ruffling. Cell *70*, 401–410.

Ridley, A.J., Schwartz, M.A., Burridge, K., Firtel, R.A., Ginsberg, M.H., Borisy, G., Parsons, J.T., and Horwitz, A.R. (2003). Cell migration: integrating signals from front to back. Science *302*, 1704–1709.

Riedl, J., Crevenna, A.H., Kessenbrock, K., Yu, J.H., Neukirchen, D., Bista, M., Bradke, F., Jenne, D., Holak, T.A., Werb, Z., et al. (2008). Lifeact: a versatile marker to visualize F-actin. Nat. Methods *5*, 605–607.

Saito, K., Ozawa, Y., Hibino, K., and Ohta, Y. (2012). FilGAP, a Rho/Rho-associated protein kinase-regulated GTPase-activating protein for Rac, controls tumor cell migration. Mol. Biol. Cell *23*, 4739–4750.

Sanz-Moreno, V., Gadea, G., Ahn, J., Paterson, H., Marra, P., Pinner, S., Sahai, E., and Marshall, C.J. (2008). Rac activation and inactivation control plasticity of tumor cell movement. Cell *135*, 510–523.

Schwanhäusser, B., Busse, D., Li, N., Dittmar, G., Schuchhardt, J., Wolf, J., Chen, W., and Selbach, M. (2011). Global quantification of mammalian gene expression control. Nature *473*, 337–342.

Semplice, M., Veglio, A., Naldi, G., Serini, G., and Gamba, A. (2012). A bistable model of cell polarity. PLoS ONE *7*, e30977.

Sha, W., Moore, J., Chen, K., Lassaletta, A.D., Yi, C.S., Tyson, J.J., and Sible, J.C. (2003). Hysteresis drives cell-cycle transitions in *Xenopus laevis* egg extracts. Proc. Natl. Acad. Sci. USA *100*, 975–980.

Symons, M., and Segall, J.E. (2009). Rac and Rho driving tumor invasion: who's at the wheel? Genome Biol. *10*, 213.

Takai, Y., Sasaki, T., and Matozaki, T. (2001). Small GTP-binding proteins. Physiol. Rev. *81*, 153–208.

Tsyganov, M.A., Kolch, W., and Kholodenko, B.N. (2012). The topology design principles that determine the spatiotemporal dynamics of G-protein cascades. Mol. Biosyst. *8*, 730–743.

Tyson, J.J., Chen, K.C., and Novak, B. (2003). Sniffers, buzzers, toggles and blinkers: dynamics of regulatory and signaling pathways in the cell. Curr. Opin. Cell Biol. *15*, 221–231.

Viaud, J., and Peterson, J.R. (2009). An allosteric kinase inhibitor binds the p21-activated kinase autoregulatory domain covalently. Mol. Cancer Ther. *8*, 2559–2565.

Volinsky, N., Gantman, A., and Yablonski, D. (2006). A Pak- and Pix-dependent branch of the SDF-1alpha signalling pathway mediates T cell chemotaxis across restrictive barriers. Biochem. J. *397*, 213–222.

von Thun, A., Preisinger, C., Rath, O., Schwarz, J.P., Ward, C., Monsefi, N., Rodríguez, J., Garcia-Munoz, A., Birtwistle, M., Bienvenut, W., et al. (2013). Extracellular signal-regulated kinase regulates RhoA activation and tumor cell plasticity by inhibiting guanine exchange factor H1 activity. Mol. Cell. Biol. *33*, 4526–4537.

Wang, Y., Ku, C.J., Zhang, E.R., Artyukhin, A.B., Weiner, O.D., Wu, L.F., and Altschuler, S.J. (2013). Identifying network motifs that buffer front-to-back signaling in polarized neutrophils. Cell Rep. *3*, 1607–1616.

Wickham, H. (2009). ggplot2: Elegant Graphics for Data Analysis, Second Edition (Springer).

Wiśniewski, J.R., Zougman, A., Nagaraj, N., and Mann, M. (2009). Universal sample preparation method for proteome analysis. Nat. Methods *6*, 359–362.

Wiśniewski, J.R., Hein, M.Y., Cox, J., and Mann, M. (2014). A "proteomic ruler" for protein copy number and concentration estimation without spike-in standards. Mol. Cell. Proteomics *13*, 3497–3506.

Wolfram Research (2010). Mathematica Edition, v.8.0 (Wolfram Research).

Xiong, W., and Ferrell, J.E., Jr. (2003). A positive-feedback-based bistable "memory module" that governs a cell fate decision. Nature *426*, 460–465.

Zenke, F.T., Krendel, M., DerMardirossian, C., King, C.C., Bohl, B.P., and Bokoch, G.M. (2004). p21-activated kinase 1 phosphorylates and regulates 14-3-3 binding to GEF-H1, a microtubule-localized Rho exchange factor. J. Biol. Chem. *279*, 18392–18400.

Zhao, Z.S., and Manser, E. (2010). Do PAKs make good drug targets? F1000 Biol. Rep. *2*, 70.

Zhao, Z.S., and Manser, E. (2012). PAK family kinases: physiological roles and regulation. Cell. Logist. *2*, 59–68.

**Cell Systems**

**Supplemental Information**

# Bistability in the Rac1, PAK, and RhoA Signaling Network Drives Actin Cytoskeleton Dynamics and Cell Motility Switches

**Kate M. Byrne, Naser Monsefi, John C. Dawson, Andrea Degasperi, Jimi-Carlo Bukowski-Wills, Natalia Volinsky, Maciej Dobrzyński, Marc R. Birtwistle, Mikhail A. Tsyganov, Anatoly Kiyatkin, Katarzyna Kida, Andrew J. Finch, Neil O. Carragher, Walter Kolch, Lan K. Nguyen, Alex von Kriegsheim, and Boris N. Kholodenko**

**Content:**

**Supplemental Figures and Files**

**Fig. S1. Related to Figure 1.** Additional experimental data
**Fig. S2. Related to Figure 2**. Illustration of the multi-dimensional dynamic analysis and visualization using Parallel Coordinates.
**Fig. S3. Related to Figure 2.** Multi-parametric dynamical analyses for 5 model state variables: Rac1, RhoA, PAK, GEF-H1 and protein 14-3-3 totals.
**Fig. S4. Related to Figure 2.** Comparative multi-parametric dynamical analyses for 5 model state variables: Rac1, RhoA, PAK, GEF-H1 and protein 14-3-3 totals.
**Fig. S5. Related to Figure 2.** Effect of kinetic parameters' variation on the bistable region in the 5D protein abundance space (identified in Fig. 2c).
**Fig. S6. Related to Figure 2.** Multi-dimensional bistability analysis of kinetic parameter variations when the protein abundances in the model are fixed at the values determined in MDA-MB-231 cells.
**Fig. S7. Related to Figure 2.** Multi-parametric dynamical analyses for 5 model state variables in the dimensionless model.
**Fig. S8. Related to Figure 2.** Dependence of bistability on PAK inhibition and the dose-response curves on parameter variations
**Fig. S9. Related to Figure 3.** Quantitation of inter-cellular IPA-3 in MDA-MB-231 cells after a drug washout
**Fig. S10. Related to Figure 3, 4, 5.** Workflow diagram of experimental validations
**Fig. S11. Related to Figure 3.** Hysteresis of pMLC and F-Actin in response to PAK inhibition.
**Figure S12. Related to Figure 5.** Bistability and bimodality is present in instantaneous cell velocities.
**Figure S13. Related to Figure 5.** PAK inhibition negatively regulates cell migration and Rac1 activity.
**Figure S14. Related to Figure 2.** Effects of additional positive feedback loops on the bistable behaviour

**Movies S1. Related to Figure 4.** Time laps of actin dynamics of untreated cells
**Movies S2. Related to Figure 4.** Time laps of actin dynamics cells treated with 1.875 $\mu$M IPA-3
**Movies S3. Related to Figure 4.** Time laps of actin dynamics cells treated with 3.75 $\mu$M IPA-3
**Movies S4. Related to Figure 4.** Time laps of actin dynamics cells treated with 7.5 $\mu$M IPA-3
**Movies S5. Related to Figure 4.** Time laps of actin dynamics cells pre-treated with 7.5 and then 0 $\mu$M IPA-3
**Movies S6. Related to Figure 4.** Time laps of actin dynamics cells pre-treated with 7.5 and then 1.875 $\mu$M IPA-3
**Movies S7. Related to Figure 4.** Time laps of actin dynamics cells pre-treated with 7.5 and then 3.75 $\mu$M IPA-3
**Movies S8. Related to Figure 4.** Time laps of actin dynamics cells pre-treated with 7.5 and then 7.5 $\mu$M IPA-3

**Supplemental Figure Legends**

**Supplemental Tables**

**Table S1. Related to Figure 1.** Reactions and reaction rates for mechanistic model.
**Table S2. Related to Figure 1.** Ordinary differential equations of mechanistic model.
**Table S3. Related to Figure 1.** Protein totals for the mechanistic model.
**Table S4 .Related to Figure 1.** Parameter values used in the mechanistic model.
**Table S5. Related to Figure 2.** Protein copy numbers and concentrations**.**
**Table S6. Related to Figure 2.** Copy numbers as determined by the proteome ruler approach across the identified proteome.

**Supplemental Experimental Procedures.**

**Mathematical Models Development, Analysis and Supplemental Methods**

**S1. Construction of a kinetic model for the integrated Rac1-PAK-RhoA pathway.**
- S1.1. Model description and assumptions
  - S1.1.1. Activation of PAK by Rac1
  - S1.1.2. PAK inhibition of RhoA through inhibition of RhoGEF
  - S1.1.3. Potential pathways of Rac1 inhibition by RhoA through Rac1 GAPs
  - S1.1.4. Inhibition of PAK by a chemical inhibitor IPA-3
- S1.2. Model reaction rates and equations

S1.3. Selection of model parameter values

**S2. Model dynamical analysis**
S2.1. Dynamical assessment based on linear stability analysis
S2.2. Bifurcation diagrams in low dimension (2D)
S2.3. Multi-dimensional analysis of model dynamics to probe the parameter space

**S3. Dimensionless model of the Rac1-PAK-RhoA system**
S3.1. Transformation to the dimensionless model
S3.2. Bistability analysis of the dimensionless model using DYVIPAC

**S4. Modelling the effects of additional positive feedbacks**
S4.1. Simplified model of the Rac1-PAK-RhoA system
S4.2. Examining the effect of added positive feedbacks

**S5. Supplemental Materials and Methods.**

**Supplemental References**

**Figure S1**

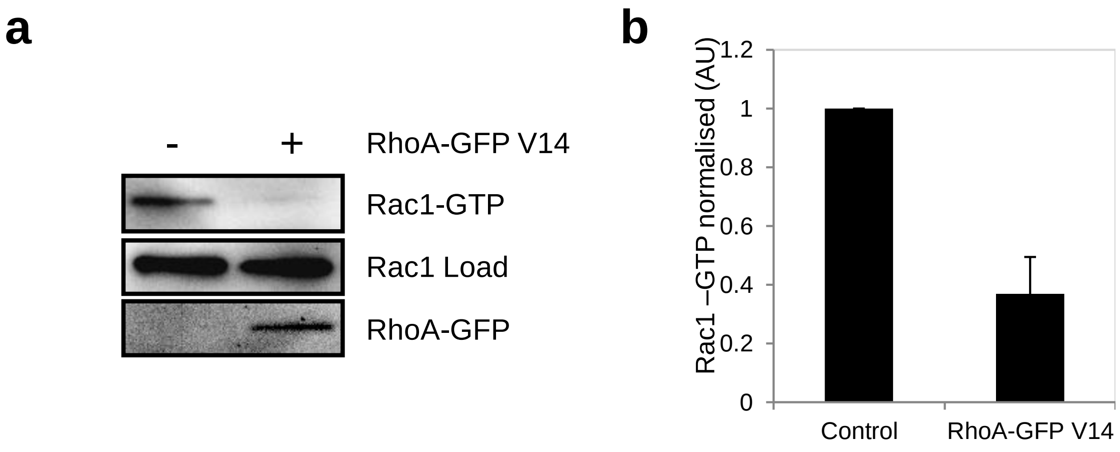
a
-
+
RhoA-GFP V14
Rac1-GTP
Rac1 Load
RhoA-GFP
b
Rac1 –GTP normalised (AU)
1.2
1
0.8
0.6
0.4
0.2
0
Control
RhoA-GFP V14

# Figure S2

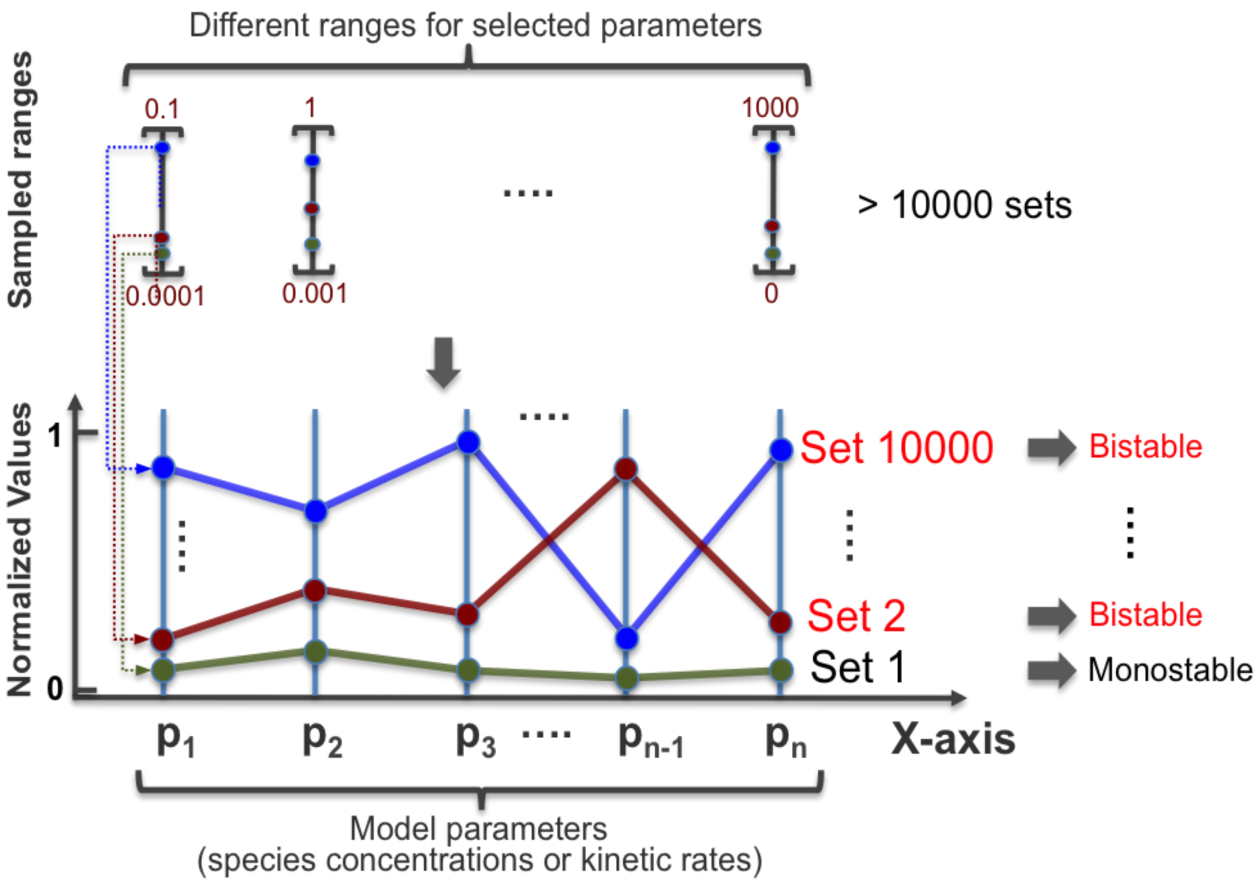

Different ranges for selected parameters
Sampled ranges
0.1
1
1000
0.0001
0.001
0
....
> 10000 sets
Normalized Values
1
0
....
Set 10000
Set 2
Set 1
Bistable
Bistable
Monostable
p1
p2
p3
....
pn-1
pn
X-axis
Model parameters
(species concentrations or kinetic rates)

# Figure S3

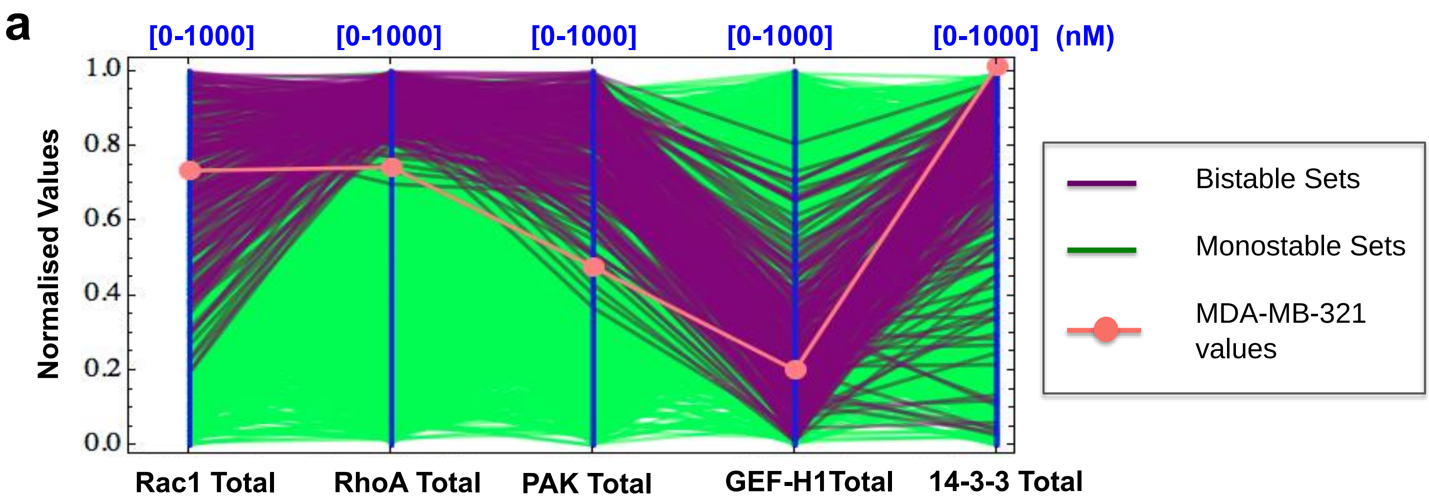
a
[0-1000]
[0-1000]
[0-1000]
[0-1000]
[0-1000] (nM)
Normalised Values
1.0
0.8
0.6
0.4
0.2
0.0
Rac1 Total
RhoA Total
PAK Total
GEF-H1Total
14-3-3 Total
Bistable Sets
Monostable Sets
MDA-MB-321 values

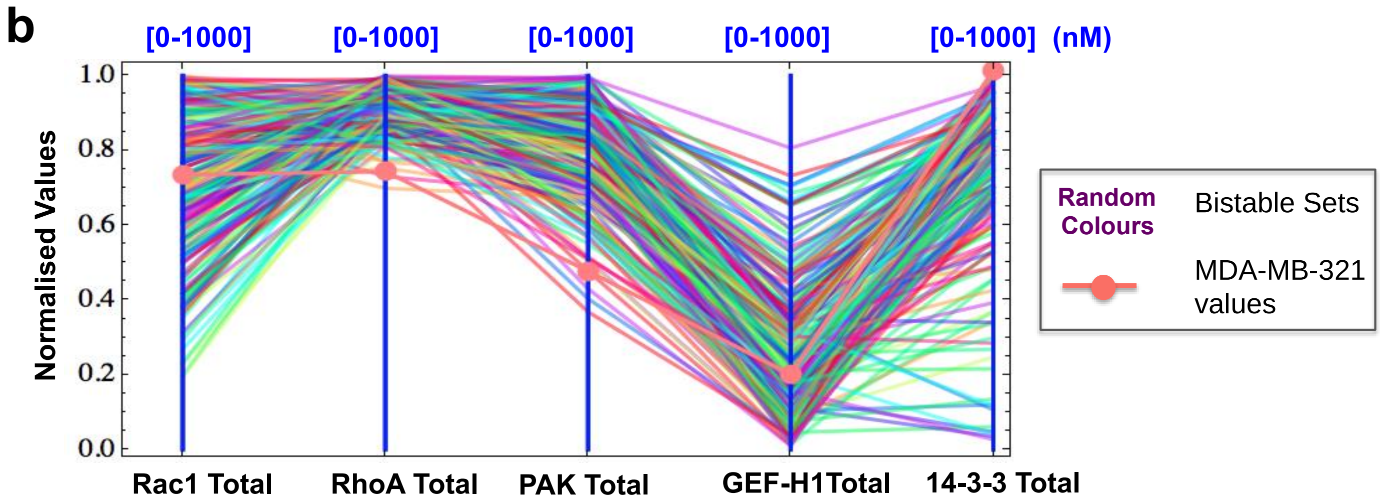
b
[0-1000]
[0-1000]
[0-1000]
[0-1000]
[0-1000] (nM)
Normalised Values
1.0
0.8
0.6
0.4
0.2
0.0
Rac1 Total
RhoA Total
PAK Total
GEF-H1Total
14-3-3 Total
Random Colours
Bistable Sets
MDA-MB-321 values

# Figure S4

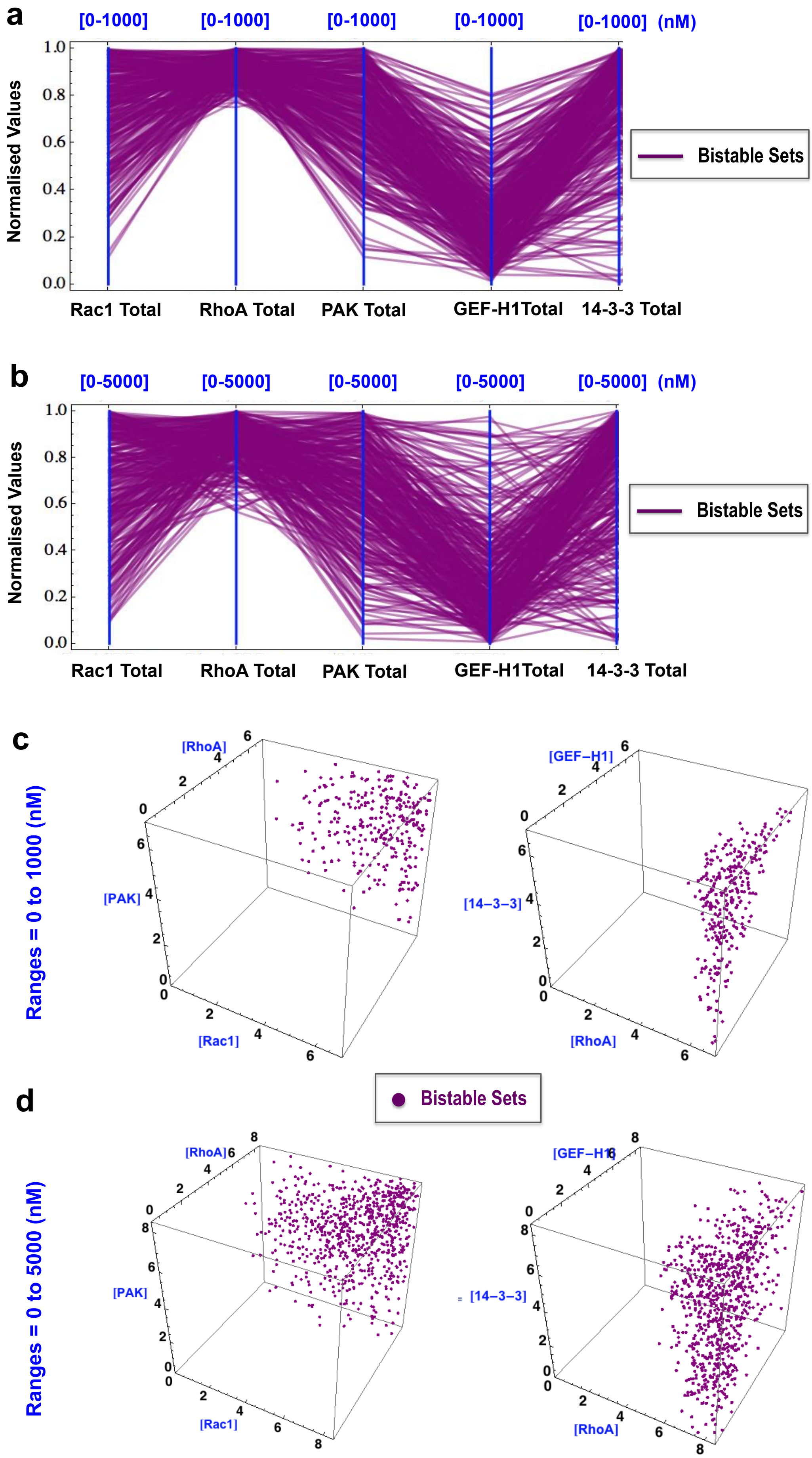
a
[0-1000]
[0-1000]
[0-1000]
[0-1000]
[0-1000] (nM)
Normalised Values
Bistable Sets
Rac1 Total
RhoA Total
PAK Total
GEF-H1Total
14-3-3 Total
b
[0-5000]
[0-5000]
[0-5000]
[0-5000]
[0-5000] (nM)
Normalised Values
Bistable Sets
Rac1 Total
RhoA Total
PAK Total
GEF-H1Total
14-3-3 Total
c
Ranges = 0 to 1000 (nM)
[RhoA]
[PAK]
[Rac1]
[GEF−H1]
[14−3−3]
[RhoA]
d
Bistable Sets
Ranges = 0 to 5000 (nM)
[RhoA]
[PAK]
[Rac1]
[GEF−H1]
[14−3−3]
[RhoA]

# Figure S5

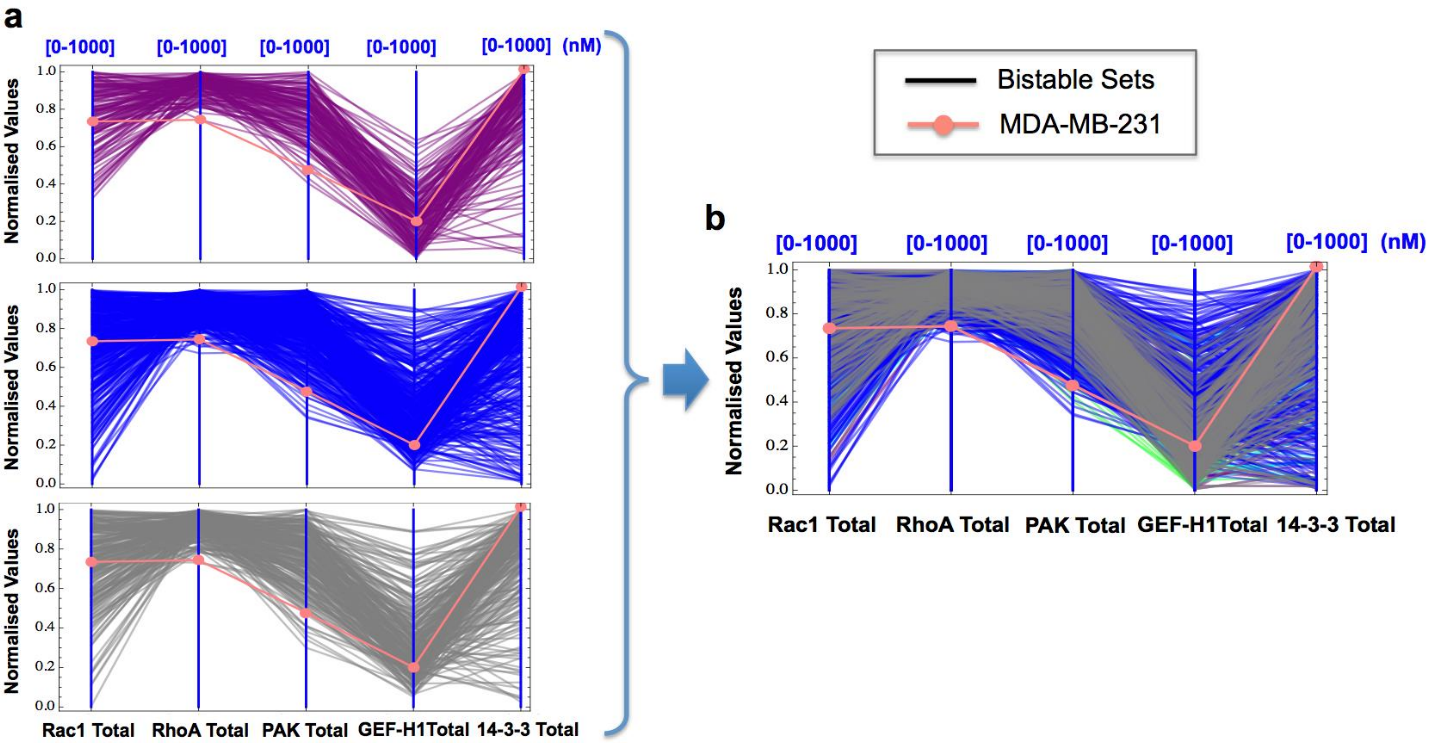
a
[0-1000]
[0-1000]
[0-1000]
[0-1000]
[0-1000] (nM)
Normalised Values
Rac1 Total
RhoA Total
PAK Total
GEF-H1Total
14-3-3 Total
Bistable Sets
MDA-MB-231
b
[0-1000]
[0-1000]
[0-1000]
[0-1000]
[0-1000] (nM)
Normalised Values
Rac1 Total
RhoA Total
PAK Total
GEF-H1Total
14-3-3 Total

# Figure S5

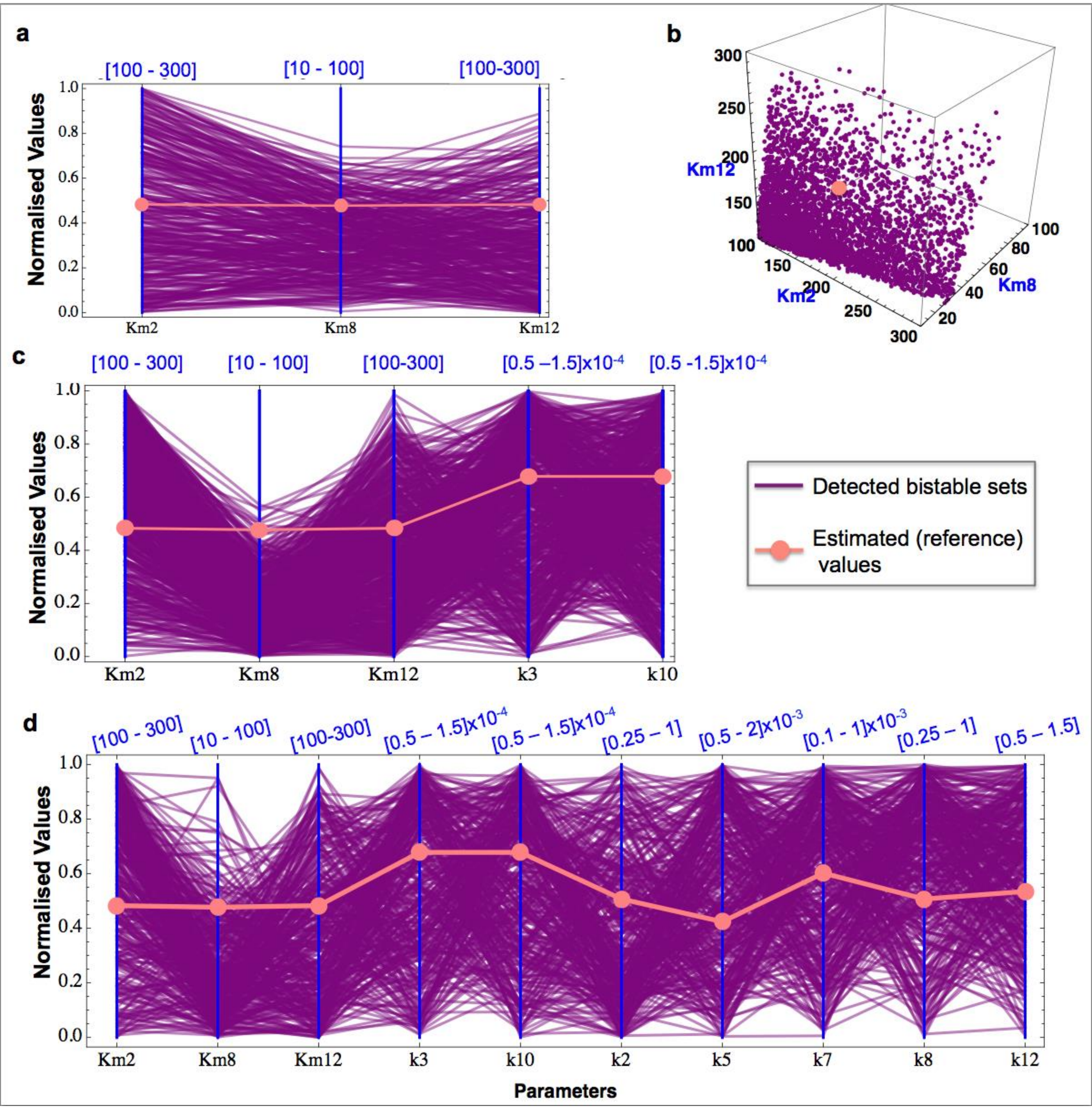

a
[100 - 300]
[10 - 100]
[100-300]
Normalised Values
Km2
Km8
Km12
b
Km12
Km2
Km8
c
[100 - 300]
[10 - 100]
[100-300]
[0.5 –1.5]x10-4
[0.5 -1.5]x10-4
Normalised Values
Km2
Km8
Km12
k3
k10
Detected bistable sets
Estimated (reference) values
d
[100 - 300]
[10 - 100]
[100-300]
[0.5 – 1.5]x10-4
[0.5 – 1.5]x10-4
[0.25 – 1]
[0.5 - 2]x10-3
[0.1 - 1]x10-3
[0.25 – 1]
[0.5 – 1.5]
Normalised Values
Km2
Km8
Km12
k3
k10
k2
k5
k7
k8
k12
Parameters

**Figure S7**

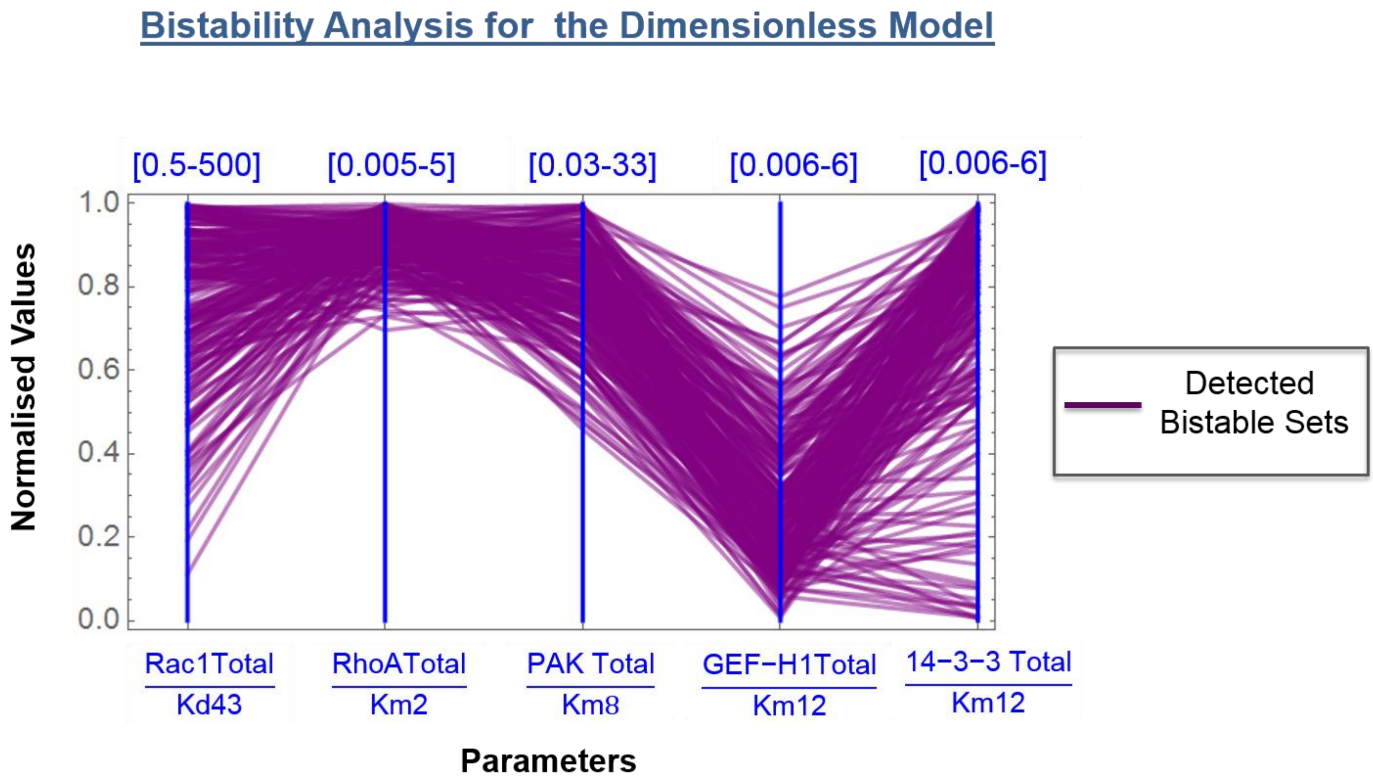
Bistability Analysis for the Dimensionless Model
[0.5-500]
[0.005-5]
[0.03-33]
[0.006-6]
[0.006-6]
1.0
0.8
0.6
0.4
0.2
0.0
Normalised Values
Rac1Total / Kd43
RhoATotal / Km2
PAK Total / Km8
GEF−H1Total / Km12
14−3−3 Total / Km12
Parameters
Detected Bistable Sets

## Figure S8

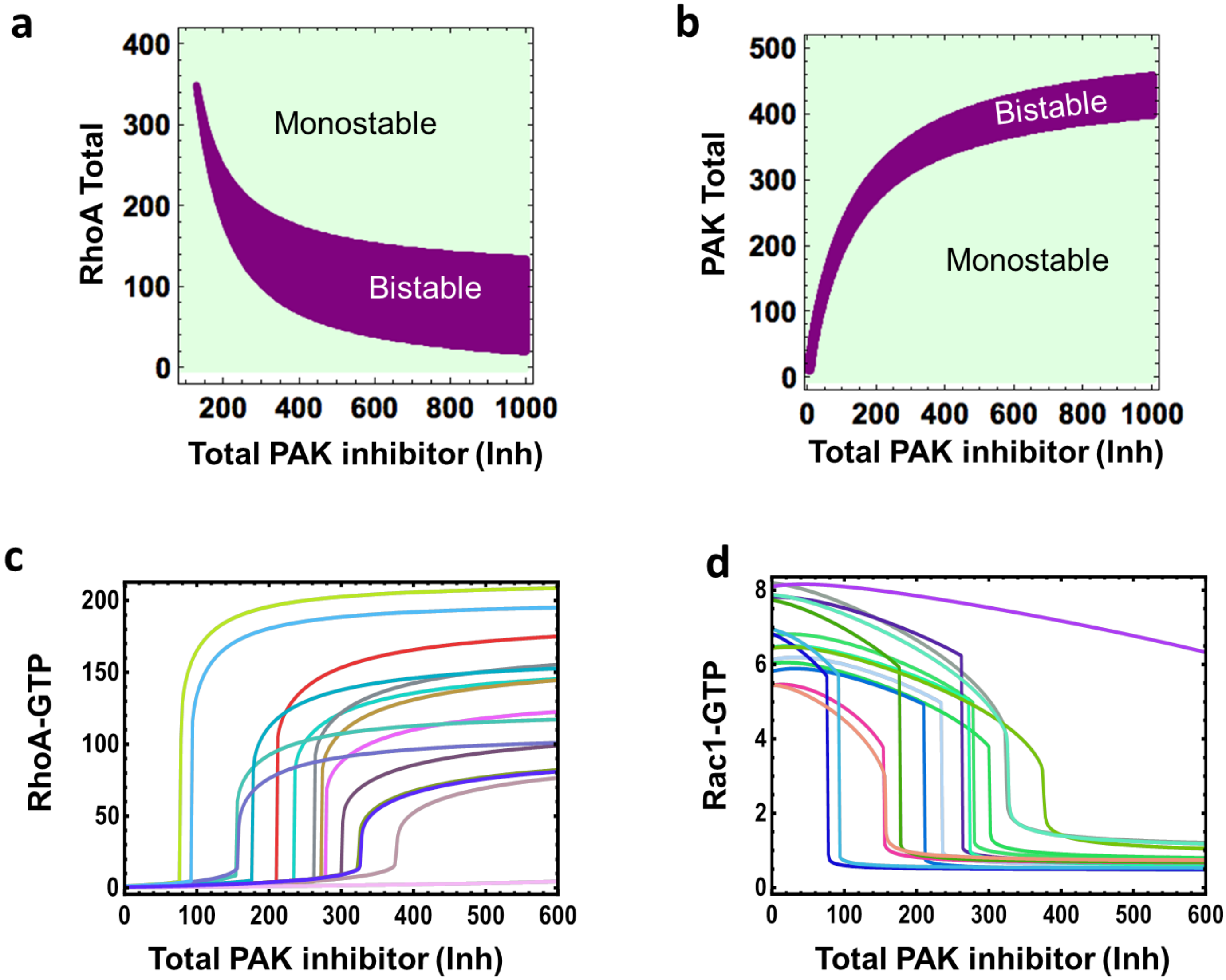

a
RhoA Total
400
300
200
100
0
Monostable
Bistable
200
400
600
800
1000
Total PAK inhibitor (Inh)
b
PAK Total
500
400
300
200
100
0
Bistable
Monostable
0
200
400
600
800
1000
Total PAK inhibitor (Inh)
c
RhoA-GTP
200
150
100
50
0
100
200
300
400
500
600
Total PAK inhibitor (Inh)
d
Rac1-GTP
8
6
4
2
0
100
200
300
400
500
600
Total PAK inhibitor (Inh)

**Figure S9**

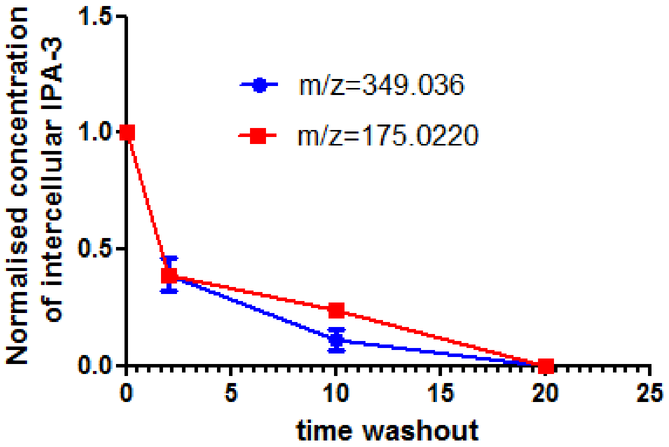

Normalised concentration of intercellular IPA-3
1.5
1.0
0.5
0.0
m/z=349.036
m/z=175.0220
0
5
10
15
20
25
time washout

# Figure S10

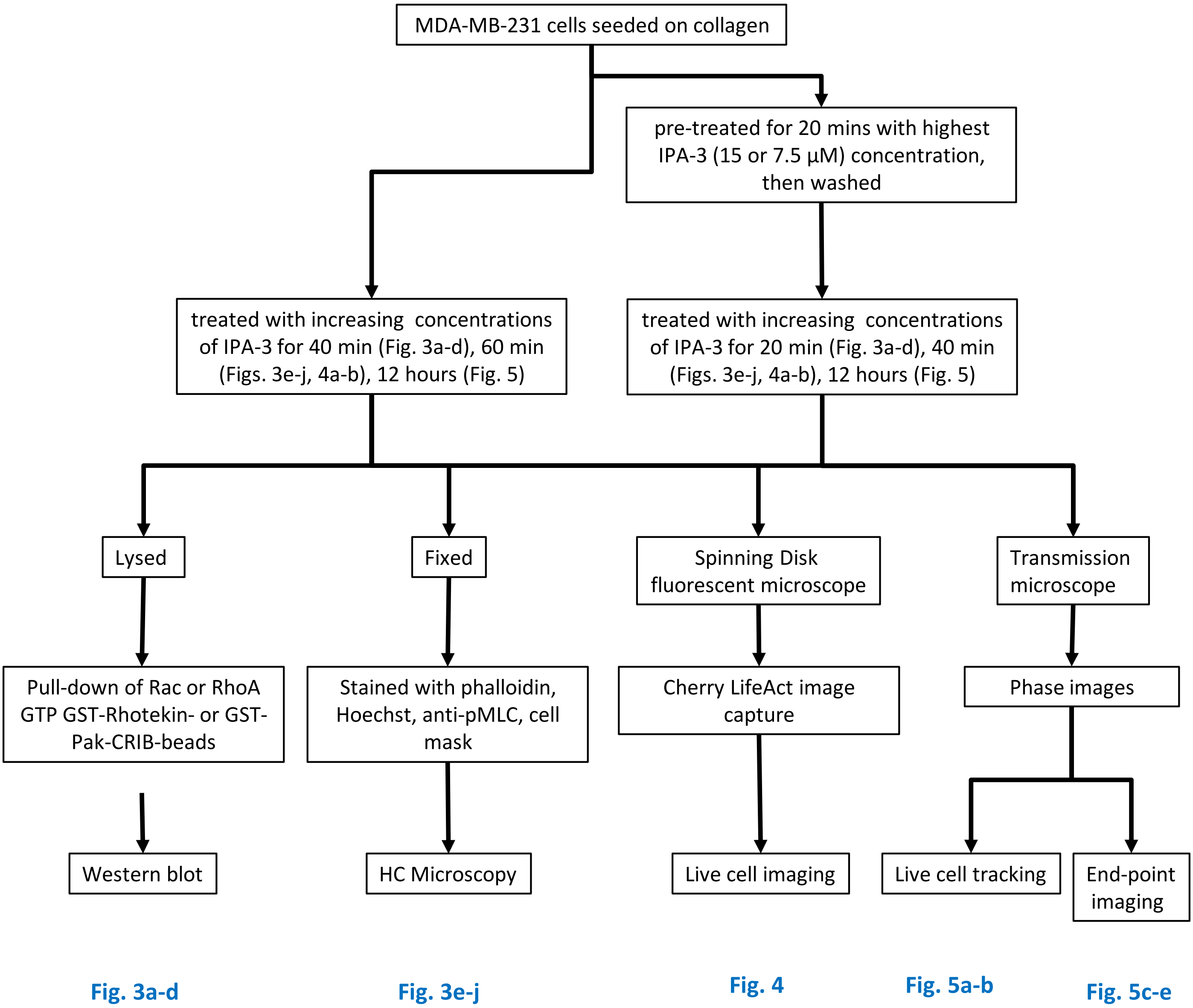
MDA-MB-231 cells seeded on collagen
pre-treated for 20 mins with highest IPA-3 (15 or 7.5 μM) concentration, then washed
treated with increasing concentrations of IPA-3 for 40 min (Fig. 3a-d), 60 min (Figs. 3e-j, 4a-b), 12 hours (Fig. 5)
treated with increasing concentrations of IPA-3 for 20 min (Fig. 3a-d), 40 min (Figs. 3e-j, 4a-b), 12 hours (Fig. 5)
Lysed
Fixed
Spinning Disk fluorescent microscope
Transmission microscope
Pull-down of Rac or RhoA GTP GST-Rhotekin- or GST-Pak-CRIB-beads
Stained with phalloidin, Hoechst, anti-pMLC, cell mask
Cherry LifeAct image capture
Phase images
Western blot
HC Microscopy
Live cell imaging
Live cell tracking
End-point imaging
Fig. 3a-d
Fig. 3e-j
Fig. 4
Fig. 5a-b
Fig. 5c-e

# Figure S11

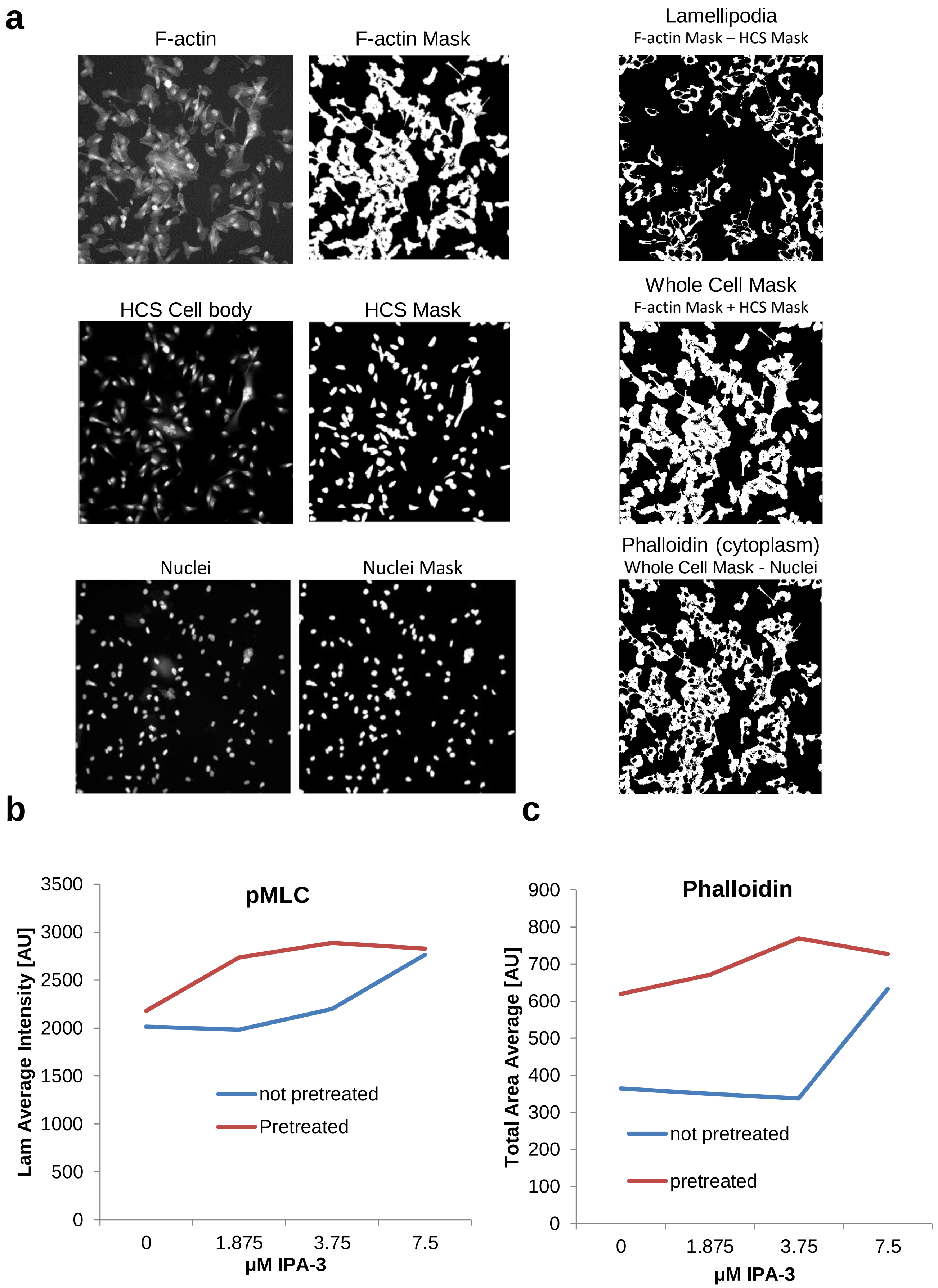
a
F-actin
F-actin Mask
Lamellipodia
F-actin Mask – HCS Mask
HCS Cell body
HCS Mask
Whole Cell Mask
F-actin Mask + HCS Mask
Nuclei
Nuclei Mask
Phalloidin (cytoplasm)
Whole Cell Mask - Nuclei
b
c
pMLC
Lam Average Intensity [AU]
3500
3000
2500
2000
1500
1000
500
0
0
1.875
3.75
7.5
µM IPA-3
not pretreated
Pretreated
Phalloidin
Total Area Average [AU]
900
800
700
600
500
400
300
200
100
0
0
1.875
3.75
7.5
µM IPA-3
not pretreated
pretreated

**Figure S12**

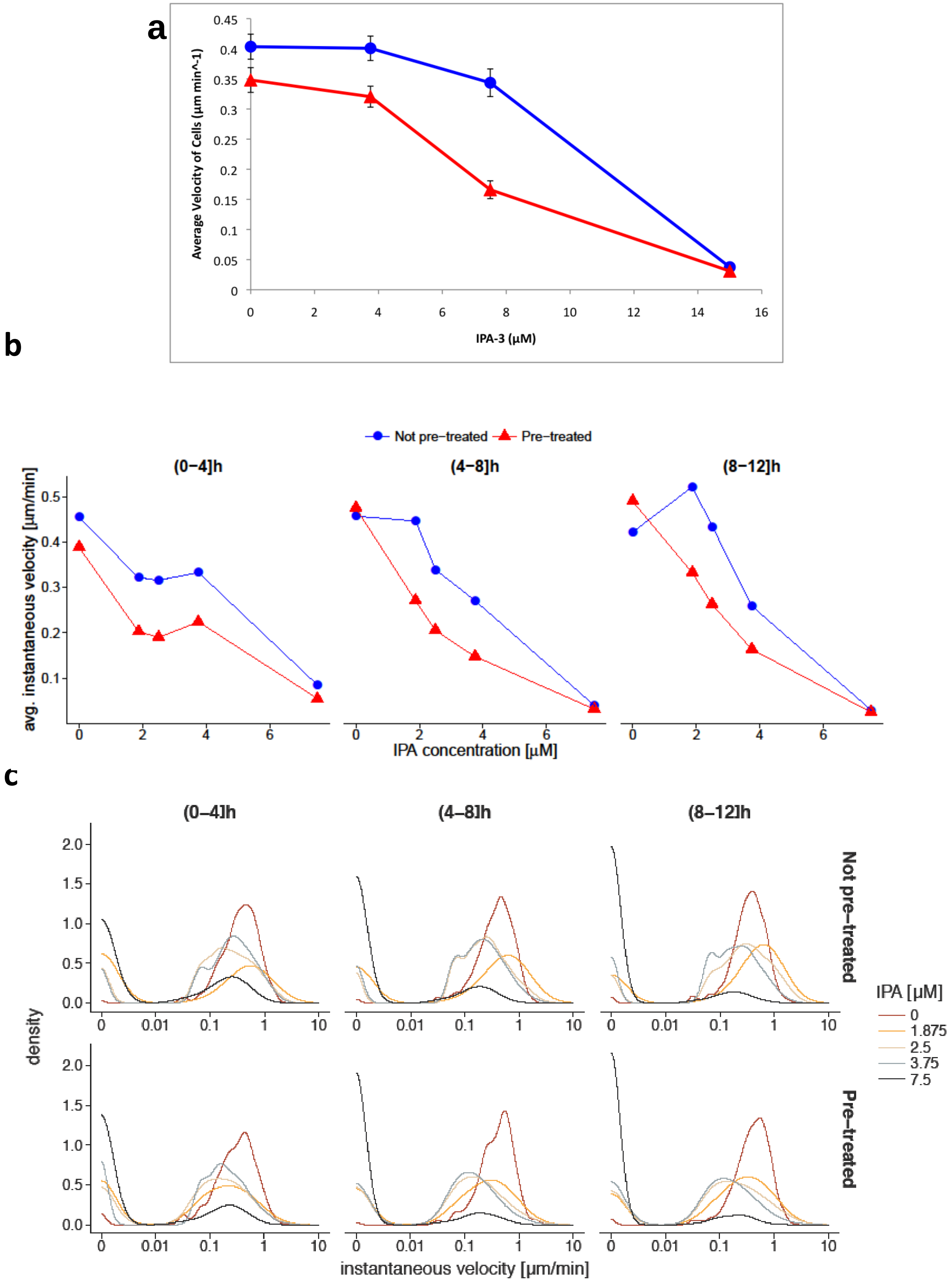
a
Average Velocity of Cells (µm min^-1)
IPA-3 (µM)
b
Not pre−treated
Pre−treated
(0−4]h
(4−8]h
(8−12]h
avg. instantaneous velocity [µm/min]
IPA concentration [µM]
c
(0−4]h
(4−8]h
(8−12]h
density
Not pre−treated
Pre−treated
IPA [µM]
0
1.875
2.5
3.75
7.5
instantaneous velocity [µm/min]

# Figure S13

**a**

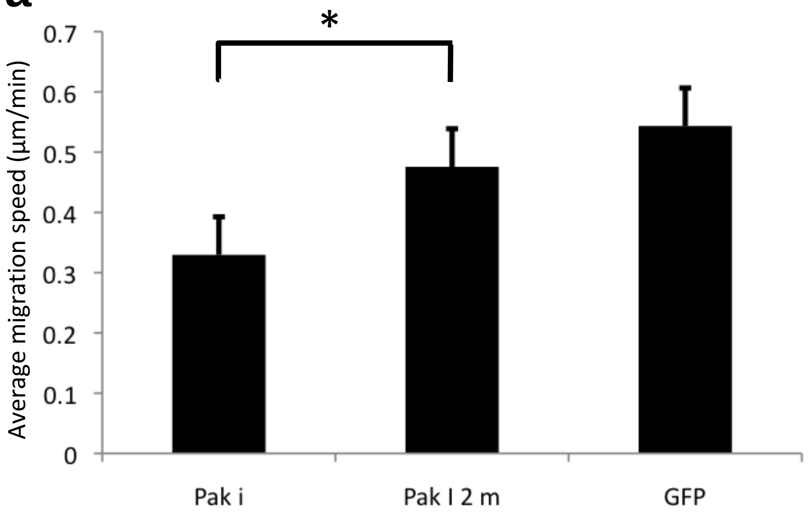


**b**

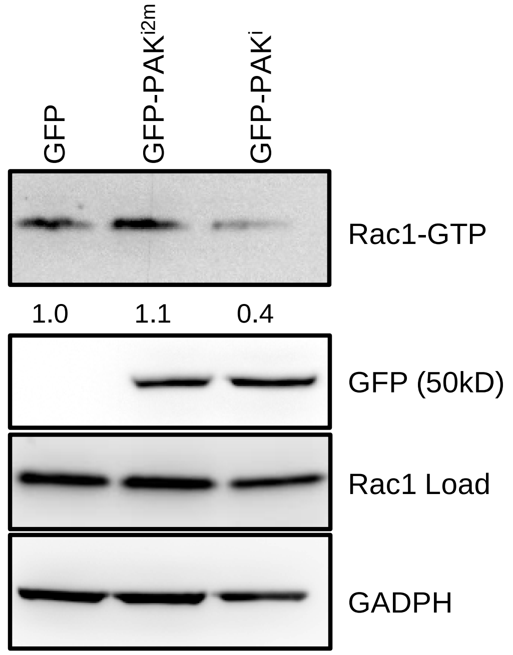

# Figure S14

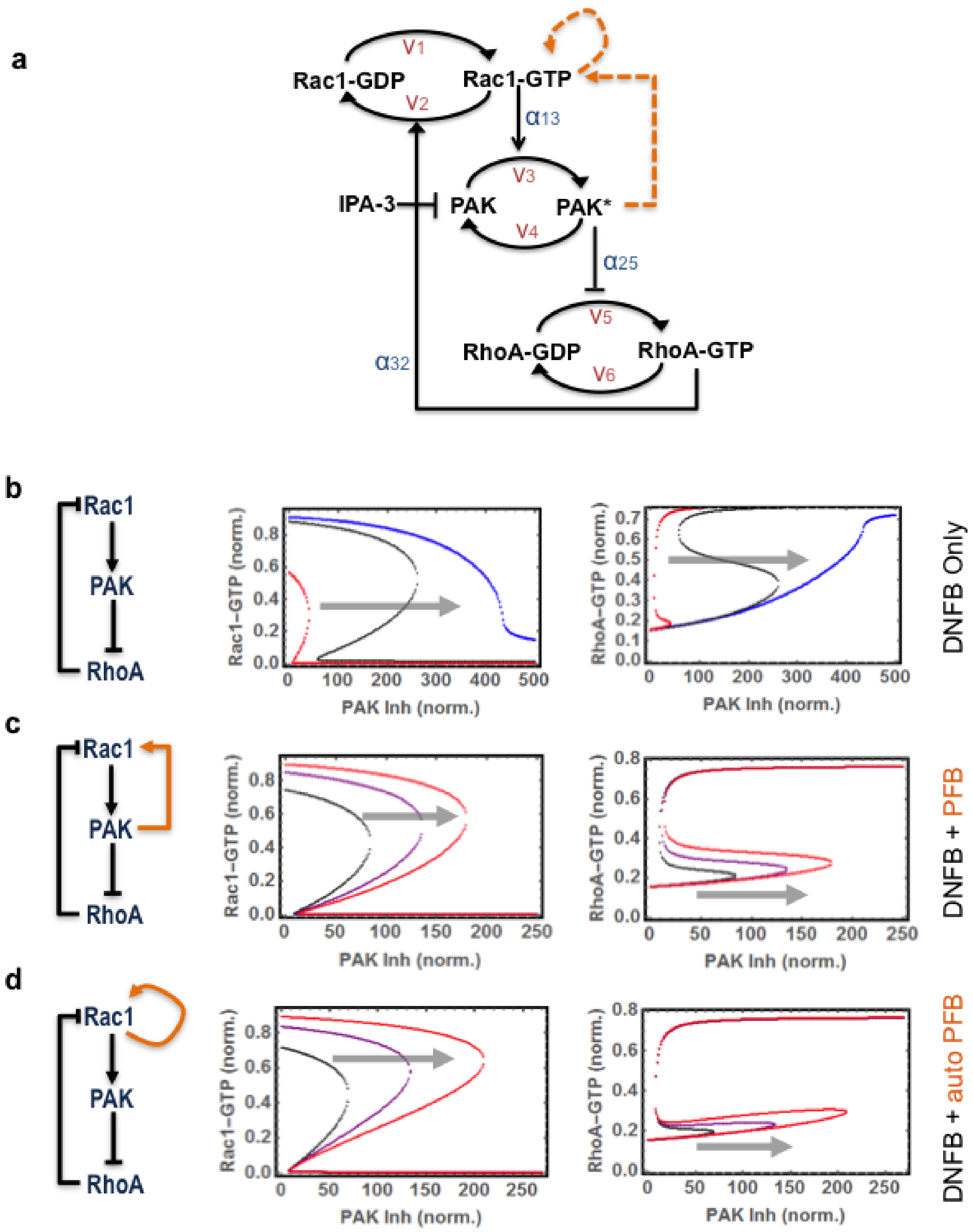
a
Rac1-GDP
Rac1-GTP
V1
V2
α13
IPA-3
PAK
PAK*
V3
V4
α25
RhoA-GDP
RhoA-GTP
V5
V6
α32
b
Rac1
PAK
RhoA
Rac1–GTP (norm.)
RhoA–GTP (norm.)
PAK Inh (norm.)
DNFB Only
c
DNFB + PFB
d
DNFB + auto PFB

## Supplemental Figure Legends

**Figure S1 Related to Figure 1.** Constitutively active RhoA Rac1-GTP **(a)** A constitutively active, EGFP tagged RhoA V14 mutant was transfected into MDA-MB-231 cells. Rac1-GTP was precipitated with GST-PAK-CRIB beads and western blotted. **(b)** Right panel represents the densitometric analysis of three biological replicates

**Figure S2**. **Related to Figure 2.** Scheme illustrating the ensemble dynamic analysis method DYVIPAC and data representation using Parallel Coordinates Plot. Large sets (typically > 10,000) of selected parameters ($p_1$, $p_2$,..., $p_n$ where typically $n \geq 3$) randomly sampled from specified ranges of values. The dynamic behaviour of the modelled system at each set is assessed using linear stability analysis, then classified (e.g. bistable or monostable, etc.) and visualized on the parallel coordinate plot. Note that all the sampling ranges are scaled to the [0,1] range to facilitate comparison and visualization of all sampled sets on the same plot. The whole process can be repeated multiple times for different selected parameters.

**Figure S3**. **Related to Figure 2.** Different presentation of the multi-dimensional dynamical analysis as in Fig. 2c (main text): **(a)** Here, the bistable sets (purple) are overlaid with a much larger number of monostable sets (green), totalling 100,000 sampled sets. The pink line indicates values measured in MDA-MB-231 cells. **(b)** Here, only the bistable sets are plotted but individual sets are coded with different colours.

**Figure S4**. **Related to Figure 2.** Comparison of the multi-dimensional dynamical analysis results for 5 parameters as in Fig.2c, main text, for different sampling range. **(a)** All the species totals are varied within 0-1000 nM while in **(b)** All the species totals are varied within 0-5000 nM. The patterns however are similar in both cases. This is further supported as observed in panels **(c)** and **(d)** where the bistable sets projected on 3D with various parameters also show consistent patterns between the small and large sampling range.

**Figure S5**. **Related to Figure 2.** Effect of kinetic parameters' variation on the bistable region in the 5D protein abundance space (identified in Fig. 2c). **(a)** Parallel coordinate plots showing the 5D bistable region for three representative parameter sets where 10 kinetic model parameters (Km2, Km8, Km12, k3, k2, k5, k7, k8, k10 and k12) are randomly sampled within 50% range around their assumed physiological values. **(b)** Superposition of plots as in panel (a) for 10 different sampled parameter sets, where the bistable region resulting from each set is coded with the same colour. The pink line represents values measured in MDA-MB-231 cells.

**Figure S6**. **Related to Figure 2.** Multi-dimensional bistability analysis of kinetic parameter variations when the protein abundances in the model are fixed at the values determined in MDA-MB-231 cells. **(a)** Parallel coordinate plot showing occurrence of bistability when 3 Michaelis-Menten constants (Km2, Km8, Km12) are sampled within large ranges containing their nominal values (pink lines). **(b)** Equivalent representation of data in panel (a) in conventional 3D graph. **(c, d)** Similar plots showing occurrence of bistability when more kinetic parameters are simultaneously sampled around their assumed physiological values. These plots together suggest that the bistable behaviour predicted for the MDA-MB-231 cells appears not to be significantly sensitive to changes in other kinetic parameters.

**Figure S7**. **Related to Figure 2.** Multi-dimensional bistability analysis for 5 independent parameter combinations in the dimensionless model (section S3, SI). These independent parameter combinations are essentially the abundance variables in the original model scaled by selected kinetic parameters, in order to reduce the dimensionality of the original model and mitigate parameter dependency related issues. The plot shows large region of bistability when these scaled independent parameters (the ratio of protein abundances (totals) and the Km/Kd values) are sampled within large ranges spanning 3 orders of magnitudes.

**Figure S8. Related to Figure 2.** Dependence of bistability on PAK inhibition and the dose-response curves on parameter variations. **(a,b)** 2D bifurcation diagrams showing dependence of bistability on changes in the total level of PAK inhibitor, Inh, and various model parameters. **(c,d)** Ensemble modelling of multiple dose-response curves of active RhoA and Rac1 against increasing Inh for 15 different sets of values of Rac1, RhoA, PAK, GEF-H1 and 14-3-3 totals, which are randomly varied within 2-fold of the reference values measured in MDA-MB-231 cells.

**Figure S9. Related to Figure 3.** Quantitation of inter-cellular IPA-3 in MDA-MB-231 cells after a drug washout. Intercellular IPA-3 was quantified by mass spectrometry in negative ion mode. We detected IPA-3 as singly charged or doubly charged ion with a mass-over-charge (m/z) of 349.036 or 175.0220 Da. Graph represent the relative concentration of intercellular IPA-3 of two biological replicates at the given time points in minutes.

**Figure S10. Related to Figure 3, 4, 5.** Workflow diagram of experimental validations

**Figure S11. Related to Figure 3.** Hysteresis of pMLC and F-Actin in response to PAK inhibition. **(a)** Imaging processing work flow: Phalloidin stain is used to determine the outline of the cells, HCS cell body stain outlines stains the nucleus and cell body and is used to outline the thicker, three dimensional regions of the cell. Hoechst stains the nucleus. Subtracting HCS mask from the F-actin mask reveals the thin, lamellipodial region of the cells, subtracting the nucleus from the F-actin mask reveals the cytoplasmatic region (b) Lamellipodial pMLC in MDA-MB-231 cells is regulated in a bistable manner by PAK. MDA-MB-231 cells were treated with the PAK inhibitor IPA-3 at the indicated concentrations for 80 minutes (blue) or by incubating MDA-MB-231 cells with 7.5 μM IPA-3 for 20 minutes. The inhibitor was subsequently washed out and the cells were incubated for an additional 60 minutes with IPA-3 at the indicated concentrations (red). Graphs represent the median of the lamellipodial average intensity. n>1000, two biological repeats (c) F-actin in MDA-MB-231 cells is regulated in a bistable manner by PAK. MDA-MB-231 cells were treated with the PAK inhibitor IPA-3 at the indicated concentrations for 80 minutes (blue) or by incubating MDA-MB-231 cells with 7.5 μM IPA-3 for 20 minutes. The inhibitor was subsequently washed out and the cells were incubated for an additional 60 minutes with IPA-3 at the indicated concentrations (red). Graphs represent the median of total cell area average intensity. n>1000, two biological repeats

**Figure S12. Related to Figure 5.** Bistability and bimodality is present in instantaneous cell velocities. **(a)** Preliminary experiments show full migration inhibition occurs at 15 μM. The IPA-3 concentrations used in Fig. 4a-b are different to those in Fig. 3a-d due to batch-to-batch variablility. Preliminary experiments here show that full inhibition of migration occurs at 15 μM, in agreement with Fig. 3c-f. The methods used were those outlined in the main text. (b,c) Bistability and bimodality is present in instantaneous cell velocities **(b)** For the duration of the experiment, the instantaneous velocities exhibit hysteretic response to IPA-3 treatment. The hysteresis and the ensuing bistability are consistent with Fig. 4b in the main text, where velocities were averaged over the entire experiment. **(c)** The instantaneous velocities for all IPA-3 treatments follow a bimodal distribution. At 0 and 7.5 μM IPA-3, all cells have near zero and maximum instantaneous velocities, respectively. At the intermediate treatments, where the system is bistable (1.865, 2.5 and 3.75 μM IPA-3), the distribution of velocities is strongly bimodal. The behaviour is present at each stage of the 12-hour experiment.

**Figure S13. Related to Figure 5.** PAK inhibition negatively regulates cell migration and Rac1 activity. **(a)** A PAK inhibitor peptide coupled to GFP ($PAK^i$) or an inactive mutant ($PAK^i$-2m) was transfected into MDA-MB-231 cells. The migration of individual cells was tracked over 12 hours using the same method as Fig. 4 a, b. The mean speed ±SD of three independent experiments are shown. The asterisk * indicates $P < 0.05$ using a two-tailed, unpaired t–test. **(b)** MDA-MB-231 cells were transfected with $PAK^i$ or $PAK^i$-2m. Rac1-GTP was precipitated with GST-Pak-CRIB beads and western blotted.

**Figure S14**. **Related to Figure 2.** Effect of additional positive feedback loops on bistability. **(a)** Schematic diagram of the simplified 3-tier Rac1-PAK-RhoA model described in section S4 of the SI. **(b)** Simulations using the model containing only the double negative feedback loop. Dependence of active Rac1 and RhoA on PAK inhibitor doses for decreasing double negative feedback strength ($b_{32}$=80, blue; 100, black; and 200, red). Bistability is observed to be more pronounced at stronger feedback and it lost for weak feedback. **(c)** Simulations using the model containing an additional Rac1-PAK positive feedback: Dependence of active Rac1/RhoA on increasing PAK inhibitor for increasing positive feedback strength ($b_{21}$=2.5, blue; 5, black; and 8, red). **(d)** Simulations conducted in the model containing an additional Rac1 auto-regulatory positive feedback: Dependence of active Rac1 and RhoA on increasing PAK inhibitor for increasing positive feedback strength ($b_{11}$=2.5, blue; 5, black; and 8, red).

**Supplemental Tables:**

**Table S1. Related to Figure 1**. Reactions and reaction rates for mechanistic model.

| **Rate number** | **Reactions** | **Reaction Rates** |
|---|---|---|
| $v_1$ | $[Rac1\text{–}GDP]\xrightarrow{[Rac1\text{–}GEF]}[Rac1\text{–}GTP]$ | $\frac{k_1\cdot[Rac1\text{–}GEF]\cdot[Rac1\text{–}GDP]}{K_{m1}+[Rac1\text{–}GDP]}$ |
| $v_2$ | $[Rac1\text{–}GTP]\xrightarrow{[Rac1\text{–}GAP]}[Rac1\text{–}GDP]$ | $\frac{k_2\cdot[Rac1\text{–}GAP]\cdot[Rac1\text{–}GTP]}{K_{m2}+[Rac1\text{–}GTP]}$ |
| $\alpha_{32}$ | | $\alpha_{32}=\frac{1+\beta_{32}\frac{[RhoA\text{–}GTP]}{K_{32}}}{1+\frac{[RhoA\text{–}GTP]}{K_{32}}}$ |
| $v_3$ | $[Rac1\text{–}GTP]+[iPAK]\rightarrow[Rac1\text{–}GTP\text{–}iPAK]$ | $k_3\cdot[Rac1\text{–}GTP]\cdot[iPAK]$ |
| $v_4$ | $[Rac1\text{–}GTP\text{–}iPAK]\rightarrow[Rac1\text{–}GTP]+[iPAK]$ | $k_4\cdot[Rac1\text{–}GTP\text{–}iPAK]$ |
| $v_5$ | $[Rac1\text{–}GTP\text{–}iPAK]\rightarrow[Rac1\text{–}GTP\text{–}pPAK]$ | $k_5\cdot[Rac1\text{–}GTP\text{–}iPAK]$ |
| $v_6$ | $[Rac1\text{–}GTP\text{–}pPAK]\rightarrow[Rac1\text{–}GTP\text{–}iPAK]$ | $k_6\cdot[Rac1\text{–}GTP\text{–}pPAK]$ |
| $v_7$ | $[Rac1\text{–}GTP\text{–}pPAK]\rightarrow[pPAK]+[Rac1\text{–}GTP]$ | $k_7\cdot[Rac1\text{–}GTP\text{–}pPAK]$ |
| $v_8$ | $[GEF\text{–}H1]\xrightarrow{[pPAK]}[pGEF\text{–}H1]$ | $\frac{k_8\cdot[pPAK]\cdot[GEF\text{–}H1]}{K_{m8}+[GEF\text{–}H1]}$ |
| $v_9$ | $[pGEF\text{–}H1]\rightarrow[GEF\text{–}H1]$ | $\frac{V_{\max 9}\cdot[pGEF\text{–}H1]}{K_{m9}+[pGEF\text{–}H1]}$ |
| $v_{10}$ | $[14\text{–}3\text{–}3]+[pGEF\text{–}H1]\rightarrow[14\text{–}3\text{–}3\text{–}pGEF\text{–}H1]$ | $k_{10}\cdot[14\text{–}3\text{–}3]\cdot[pGEF\text{–}H1]$ |
| $v_{11}$ | $[14\text{–}3\text{–}3\text{–}pGEF\text{–}H1]\rightarrow[14\text{–}3\text{–}3]+[pGEF\text{–}H1]$ | $k_{11}\cdot[14\text{–}3\text{–}3\text{–}pGEF\text{–}H1]$ |
| $v_{12}$ | $[RhoA\text{–}GDP]\xrightarrow{[GEF\text{–}H1]}[RhoA\text{–}GTP]$ | $\frac{k_{12}\cdot[GEF\text{–}H1]\cdot[RhoA\text{–}GDP]}{K_{m12}+[RhoA\text{–}GDP]}$ |
| $v_{13}$ | $[RhoA\text{–}GTP]\xrightarrow{[RhoA\text{–}GAP]}[RhoA\text{–}GDP]$ | $\frac{k_{13}\cdot[RhoA\text{–}GAP]\cdot[RhoA\text{–}GTP]}{K_{m13}+[RhoA\text{–}GTP]}$ |
| $v_{14}$ | $[IPA\text{–}3]+[iPAK]\rightarrow[IPA\text{–}3\text{–}iPAK]$ | $k_{14}\cdot[IPA\text{–}3]\cdot[iPAK]$ |
| $v_{15}$ | $[IPA\text{–}3\text{–}iPAK]\rightarrow[IPA\text{–}3]+[iPAK]$ | $k_{15}\cdot[IPA\text{–}3\text{–}iPAK]$ |
| $v_{16}$ | $[pPAK]\rightarrow[iPAK]$ | $k_{16}\cdot[pPAK]$ |

**Table S2. Related to Figure 1.** Ordinary differential equations of mechanistic model.

| **Left hand side** | **Right hand side** |
|---|---|
| d[Rac1-GDP]/dt | $-v_1 + v_2*\alpha_{32}$ |
| d[Rac1-GTP]/dt | $v_1 - v_2* \alpha_{32} - v_3 + v_4 + v_7$ |
| d[IPA-3]/dt | $-v_{14} + v_{15}$ |
| d[IPA-3-iPAK]/dt | $v_{14} - v_{15}$ |
| d[iPAK]/dt | $-v_3 + v_4 - v_{14} + v_{15} + v_{16}$ |
| d[Rac1-GTP-pPAK]/dt | $v_5 - v_6 - v_7$ |
| d[Rac1-GTP-iPAK]/dt | $v_3 - v_4 - v_5 + v_6$ |
| d[pPAK]/dt | $v_7 - v_{16}$ |
| d[protein14-3-3]/dt | $-v_{10} + v_{11}$ |
| d[GEF-H1]/dt | $-v_8 + v_9$ |
| d[pGEF-H1]/dt | $v_8 - v_9 - v_{10} + v_{11}$ |
| d[pGEF-H1-protein14-3-3]/dt | $v_{10} - v_{11}$ |
| d[RhoA-GDP]/dt | $-v_{12} + v_{13}$ |
| d[RhoA-GTP]/dt | $v_{12} - v_{13}$ |

**Table S3. Related to Figure 1.** Protein totals for the mechanistic model.

| | **Totals** |
|---|---|
| Rac1$_{TOT}$ | $[Rac1{-}GTP]+[Rac1{-}GDP]+[Rac1{-}GTP{-}iPAK]+[Rac1{-}GTP{-}pPAK]$ |
| IPA-3$_{TOT}$ | $[IPA{-}3]+[IPA{-}3{-}iPAK]$ |
| PAK$_{TOT}$ | $[iPAK]+[IPA{-}3{-}iPAK]+[Rac1{-}GTP{-}iPAK]+[Rac1{-}GTP{-}pPAK]+[pPAK]$ |
| 14-3-3$_{TOT}$ | $[14{-}3{-}3]+[14{-}3{-}3{-}pGEF{-}H1]$ |
| GEF-H1$_{TOT}$ | $[GEF{-}H1]+[pGEF{-}H1]+[14{-}3{-}3]+[14{-}3{-}3{-}pGEF{-}H1]$ |
| RhoA$_{TOT}$ | $[RhoA{-}GTP]+[RhoA{-}GDP]$ |

**Table S4. Related to Figure 1.** Parameter values used in the mechanistic model.
Concentrations and the Michaelis-Menten constants ($K_m$) are given in nM. Protein concentrations are expressed in nM. First- and second-order rate constants are expressed in $s^{-1}$ and $nM^{-1}\ s^{-1}$. Maximum rates $V_{max}$ are expressed in nM $s^{-1}$. $\beta_{32}$ and $K_{32}$ are dimensionless parameters.

| Parameter | Value | References |
|---|---|---|
| $k_1$ | 0.06 | Estimated based on typical ranges |
| Rac1-GEF | 8.5 | (Nagaraj et al., 2011) |
| $K_{m1}$ | 130 | Estimated |
| $k_2$ | 0.505 | Estimated |
| Rac1-GAP | 15 | (Nagaraj et al., 2011) |
| $K_{m2}$ | 200 | Estimated |
| $\beta_{32}$ | 130 | (Tsyganov et al., 2012) |
| $K_{32}$ | 1650 | (Tsyganov et al., 2012) |
| $k_3$ | 0.0001 | Estimated |
| $k_4$ | 0.0002 | Estimated |
| $k_5$ | 0.0009 | Estimated |
| $k_6$ | 0.00035 | Estimated |
| $k_7$ | 0.0004 | Estimated |
| $k_8$ | 0.505 | Estimated |
| $K_{m8}$ | 30 | Estimated |
| $V_{max9}$ | 5.05 | Estimated |
| $K_{m9}$ | 20 | Estimated |
| $k_{10}$ | 0.0001 | Estimated |
| $k_{11}$ | 0.001 | Estimated |
| $k_{12}$ | 0.9 | Estimated |
| $K_{m12}$ | 170 | Estimated |
| $k_{13}$ | 0.06 | Estimated |
| $K_{m13}$ | 10 | Estimated |
| RhoA-GAP | 10 | (Nagaraj et al., 2011) |
| $k_{14}$ | 0.001 | Estimated |
| $k_{15}$ | 0.01 | Estimated |
| $k_{16}$ | 0.0005 | Estimated |
| $Rac1_{TOT}$ | 160 | Measured in this study |
| $PAK_{TOT}$ | 27 | Measured in this study |
| $RhoA_{TOT}$ | 161 | Measured in this study |
| $IPA\text{-}3_{TOT}$ | 0 (or 270) (*) | |
| $GEF\text{-}H1_{TOT}$ | 4 | Measured in this study |
| $14\text{-}3\text{-}3_{TOT}$ | 1200 | Measured in this study |

(*) Total IPA-3 = 0 when the model was simulated with no PAK inhibitor. IPA-3 total = 270 nM when the model was simulated with PAK inhibitor (at which the system is bistable, as seen in Fig. 2e,f in the main text).

**Table S5. Related to Figure 2.** Protein copy numbers and concentrations.

| Gene names | Protein name | Copy number Intensity | Concentration (nM) (*) |
|---|---|---|---|
| PAK2 | Serine/threonine-protein kinase PAK 2 | **661,552** | **27** |
| RAC2 | Ras-related C3 botulinum toxin substrate 2 | 277,301 | |
| RAC1;RAC3 | Ras-related C3 botulinum toxin substrate 1;Ras-related C3 botulinum toxin substrate 3 | **3,984,262** | **160** |
| ARHGEF7 | Rho guanine nucleotide exchange factor 7 | 20,973 | |
| ARHGEF12 | Rho guanine nucleotide exchange factor 12 | 3,610 | |
| ARHGEF10 | Rho guanine nucleotide exchange factor 10 | 3,718 | |
| ARHGEF1 | Rho guanine nucleotide exchange factor 1;ARHGEF1 protein | 124,608 | |
| ARHGEF18 | Rho guanine nucleotide exchange factor 18 | 19,482 | |
| ARHGEF2 | Rho guanine nucleotide exchange factor 2 | **100,443** | **4** |
| ARHGEF28 | Rho guanine nucleotide exchange factor 28 | 6,658 | |
| RHOT1 | Mitochondrial Rho GTPase 1;Mitochondrial Rho GTPase | 7,391 | |
| RHOC | Rho-related GTP-binding protein RhoC | 530,247 | |
| RHOA;RHO C | Transforming protein RhoA;Rho-related GTP-binding protein RhoC | **4,013,548** | **161** |
| RHOG | Rho-related GTP-binding protein RhoG | 490,041 | |
| RHOT2 | Mitochondrial Rho GTPase 2 | 7,850 | |
| RHOF | Rho-related GTP-binding protein RhoF | 25,597 | |
| | | | |
| YWHAQ | 14-3-3 protein theta | 3,147,878 | |
| YWHAB | 14-3-3 protein beta/alpha | 3,602,803 | |
| YWHAB | 14-3-3 protein beta/alpha | 587,444 | |
| SFN | 14-3-3 protein sigma | 1,937,124 | |
| YWHAG | 14-3-3 protein gamma | 385,879 | |
| YWHAE | 14-3-3 protein epsilon | 9,115,957 | |
| YWHAZ | 14-3-3 protein zeta/delta | 10,513,216 | |
| YWHAH | 14-3-3 protein eta | 456,572 | |
| | Total 14-3-3 | **29,746,874** | **1200** |

(*) Concentrations were calculated assuming an average cell volume = 4 x $10^{-14}$(L). It is worth noting that our simulations show that bistablity depends on the relative rather than absolute concentrations of these species; therefore a larger or smaller cell volume will not affect our results.

**Supplemental Experimental Procedures.**

## S1. Construction of a kinetic model for the integrated Rac1-PAK-RhoA pathway.

In this section, we present description and assumptions of a kinetic and dynamic model of the Rac1-PAK-RhoA interaction network used for simulation and analysis in the main text (the model scheme is given in Fig. 1a of the main text). This model was aimed to capture the up-to-date details of the network interactions in light of experimental evidence, incorporating protein-protein interactions, phosphorylation events and feedback regulation. The model was formulated using the laws of mass-action and enzymatic Michaelis-Menten (MM) kinetics. Note that IPA-3 is explicitly used in the following model reactions and equations instead of the general inhibitor INH depicted in Fig. 1.

### S1.1. Model description and assumptions

#### *S1.1.1. Activation of PAK by Rac1*

Membrane bound Rac1-GTP recruits p21-activated kinases (PAKs) by binding to PAK's Cdc42-Rac interactive binding (CRIB)-domain, causing PAK to undergo a conformational switch. This exposes its activation loop, which is subsequently auto-phosphorylated through an intra-molecular mechanism, resulting in full activation of the kinase (Bokoch, 2003; Zhao and Manser, 2012). The mass-action model reaction scheme is given in Fig. 1a.

#### *S1.1.2. PAK inhibition of RhoA through inhibition of RhoGEF*

Activated PAK phosphorylates GEF-H1, a GEF for RhoA, on inactivating inhibitory sites (Zenke et al., 2004) Following phosphorylation, GEF-H1 binds to 14-3-3 protein which causes its relocation to microtubules (Zenke et al., 2004) where it has been shown to have decreased GEF activity (Krendel et al., 2002) In this way, Rac1 inhibits RhoA activity through PAK (Fig. 1a).

#### *S1.1.3. Potential pathways of Rac1 inhibition by RhoA through Rac1 GAPs*

We have shown experimentally that expression of constitutively active RhoA (RhoAV14) in MDA-MB-231 cells decreases the amount of active Rac1 (Fig. S1). There are several potential routes for the inhibition of Rac1 by RhoA including through the regulation of RacGAPs, e.g. ARHGAP22 (Sanz-Moreno et al., 2008) and FilGAP (Saito et al., 2012), which are phosphorylated and activated by the Rho effector kinase ROCK. In our kinetic model, we assume that active RhoA deactivates Rac1 via activation of Rac1 GAPs (Fig.1a). In addition, due to the lack of exact mechanistic detail, we describe this negative regulation in a generic way using a dimensionless multiplier that captures Rac1 GAPs activation specifying the mechanism of interaction.

Generally, we denote this multiplier $\alpha_{ij}$, where the index $i$ refers to the rate equation number being modified, and $j$ refers to the protein that modifies the regulation. This modeling approach and notation follow our previous publication (Tsyganov et al., 2012).

$$\alpha_{ij} = \frac{1+\beta_{ij}\dfrac{P_j}{K_{ij}}}{1+\dfrac{P_j}{K_{ij}}} \qquad \text{(S1)}$$

$P_j$ represents the concentration of the protein $j$. The coefficient $\beta_{ij}$ determines the maximal degree of regulation. If $\beta_{ij} < 1$, $\alpha_{ij}$ describes downregulation. If $\beta_{ij} > 1$, the term $\alpha_{ij}$ describes upregulation, as in the case of Rac1-GTP inhibition of RhoA-GTP here by upregulation of Rac1-GAPs. $K_{ij}$ is the activation or inhibition constant. In this scheme, Rac1-GTP, pPAK and RhoA-GTP are labelled as proteins 1, 2 and 3 respectively.

In the case where RhoA-GTP ($P_3$) stimulates the deactivation of Rac1 (rate $v_2$, TableS1), the corresponding multiplier reads:

$$\alpha_{32} = \frac{1+\beta_{32}\dfrac{[RhoA\text{–}GTP]}{K_{32}}}{1+\dfrac{[RhoA\text{–}GTP]}{K_{32}}}$$

#### *S1.1.4. Inhibition of PAK by a chemical inhibitor IPA-3*

Our model-based simulations and analysis suggested that PAK perturbation by inhibition is a promising way to reveal bistable behaviour for the network components (see main text). To selectively inhibit PAK in our experiments, we use IPA-3, a specific chemical inhibitor for PAK. It is known that Rac1 activates PAK by binding

directly to PAK's regulatory domain, which relieves its autoinhibition and the resulting conformational change leads to PAK autophosphorylation (Bokoch, 2003). IPA-3 is a highly selective, non-ATP-competitive inhibitor that binds directly to the regulatory domain of inactive PAK1-3 and thus prevents the Rac1 dependent activation of PAKs in a dose dependent manner. Consequently, IPA-3 has a substantially reduced effect on already active PAK (Deacon et al., 2008; Viaud and Peterson, 2009). We modelled the series of steps of IPA-3 mediated inhibition of PAK using mass action kinetics and corresponding reaction rates $v_{14}$, $v_{15}$, and $v_{16}$ can be found in Table S1.

### S1.3. Selection of parameter values

As many of the kinetic parameters in the Rac1-PAK-RhoA system are unknown at the present time, the parameter values used as the reference set for model analysis were guided by typical ranges of physiological values and constrained by experimental data where possible (Table S4). For example, the association of protein molecules into dimers or larger complexes occurs with typical rate constants of the order of $10^{-4}$ to $10^{-1}$ $nM^{-1}$ $s^{-1}$ (Kholodenko et al., 1999) In addition, the reaction rates were always constrained to be not faster than the diffusion limit. Moreover, it is worthwhile to note that a major aim of modelling is to provide a basis for guiding experimental analysis and testing explicit hypotheses; a model by itself is not an objective "truth," but it can be used to falsify or confirm a specific hypothesis. Therefore, comprehensive systematic parameter exploration compatible with experimentally observed behaviour constitutes an appropriate approach to mitigate the lack of measured parameters. This is the approach we adopted in this study.

Since abundances of Rac1, RhoA, PAK and other proteins are not available for MDA-MB-231 cells, plausible concentration ranges were based on those reported by quantitative proteomic studies reported for HeLa (Nagaraj et al., 2011) and U2OS (Beck et al., 2011) cell lines. The cell volumes used for calculation in HeLa and U2OS cell lines were $2.6 \times 10^3$ µm$^3$ (Zhao et al., 2008) and $4 \times 10^3$ µm$^3$ (Beck et al., 2011) respectively. For each protein, we divide the molecular weight (given in Da or g mol$^{-1}$) by Avagadro's constant ($6.02\times10^{23}$ mol$^{-1}$) to obtain the mass per molecule in grams. This is then multiplied by the number of copies per cell to obtain the mass of protein per cell, *m*. Therefore the molarity is obtained from the equation

$$C = \frac{p}{V \cdot A}$$

where *p* is the number of copies per cell, *V* is the cell volume (L) and *A* is Avagadro's constant.

For the unspecific Rac1-GEF, Rac1-GAP and RhoA-GAP, concentrations based on the average of several common Rac1 or RhoA GEFs and GAPs, including p114-RhoGEF, ARH-GEF17, RacGAP1, p50-RhoGAP and p105-RhoGAP (Beck et al., 2011; Nagaraj et al., 2011). Selected values for $V_{max}$ and $K_m$ are based on typical values. Values for $V_{max}$ are assumed to be in the range $10^{-1}$ to $10^2$ (Kholodenko et al., 1999). Parameters $\beta_{32}$ and $K_{32}$ are within parameter ranges used in (Tsyganov et al., 2012). In this paper, the parameter values given are normalized with respect to the total protein concentration and so parameter values are for the normalized activation or inhibition constant $m_{ij} = G_i^{tot}/K_{ij}$, where $G_i^{tot}$ is the total concentration of protein *i*. As the parameters in our models are not normalized, we obtain values for $K_{ij}$ from the formula $K_{ij}=m_{ij}$* (Tsyganov et al., 2012), using the total concentration ranges described previously.

## 2. Model dynamical analysis

### 2.1. Dynamical assessment based on linear stability analysis

Tools of nonlinear dynamics provide a useful framework to assess the dynamical properties of the Rac1-PAK-RhoA model described above (equations in Table S2). We are mainly interested in the asymptotic states and transitions between different dynamic regimes, in particular bistable and non-bistable (mostly fixed point) dynamics in this case. The steady states of the system are obtained by equating the right hand side (RHS) of the system ODEs to zero and solving for the values of the model states' concentrations. An important point to note is that it is convenient here and necessary for the next steps to reduce the full algebraic system of equations to minimal form containing only independent equations using the conditions of concentration conservation (conservation laws). These conditions are the total mass conservation for the state variables Rac1, PAK, RhoA, GEF-H1 and 14-3-3 for the mechanistic model; and Rac1, PAK, RhoA for the phenomenological model.

A solution different from the trivial one (all the species equal to zero) can exist and the temporal evolution of the system can be described by the independent selected species. If we assume that $S_{\text{indep}}$ is the vector composed by these independent concentrations, the temporal evolution of the systems is completely determined by

$$\frac{d\boldsymbol{S}_{\text{indep}}}{dt} = f(\boldsymbol{S}_{\text{indep}}), \qquad \text{(S2)}$$

where $f(S_{\text{indep}})$ is the vector formed by the reaction rates of the independent selected species. The steady states denoted by $S^0_{\text{indep}}$ are obtained by solving $f(S^0_{\text{indep}})=0$. The asymptotic stability of these steady states can be determined by linear analysis upon perturbations. Thus, in the vicinity of any of these steady states the temporal evolution of a perturbation from this state, denoted by $\Delta S^0_{\text{indep}} = S_{\text{indep}} - S^0_{\text{indep},}$ is given by

$$\frac{d\Delta\boldsymbol{S}_{\text{indep}}}{dt} = J(\boldsymbol{S}_{\text{indep}})\Delta\boldsymbol{S}^0_{\text{indep}}, \qquad \text{(S3)}$$

where $J(S^0_{\text{indep}})$ is the Jacobian matrix of the reduced system evaluated at the considered steady state. Thus, the dynamical behavior of the system is entirely specified by the Jacobian matrix and by its eigenvalues and eigenvectors. If we assume that the eigenvalues of $J(S^0_{\text{indep}})$ for a given steady state $S^0_{\text{indep}}$, denoted by $\lambda_i$ (i=1,2,..n where n is the number of independent species) are ordered decreasingly by the values of their real part, Re($\lambda_i$), then the dominant growth term of the perturbation is governed by

$$\Delta\boldsymbol{S}^0_{\text{indep}}(t) \propto \boldsymbol{E}_1 e^{\text{Re}(\lambda_1)t}, \qquad \text{(S4)}$$

where $E_1$ is the eigenvector corresponding to the eigenvalue with the largest real part. The state $S^0_{\text{indep}}$ is asymptotically stable if, and only if, Re($\lambda_1$) is negative and $\Delta S^0_{\text{indep}}$ tends to exponentially decrease in the course of time. If there is at least one positive eigenvalues' real part (i.e. at least Re($\lambda_i$) is positive) then the perturbation grows exponentially and $S^0_{\text{indep}}$ is unstable in response to the perturbation.

In order to study the asymptotic states and the transition between different dynamical behaviors, we have solved numerically the system for the parameters values given in Tables S4 and S7 (and other explored values mentioned in the text) using Mathematica 8.0. Although linear models can generally be solved analytically, non-linear models like ours cannot usually be solved analytically and resorting to numerical solvers are required. For the Rac1-PAK-RhoA model and the considered parameter ranges, only one or three real positive solutions are possible. Once the solutions have been obtained, we have substituted them into the Jacobian Matrix and numerically calculated their eigenvalues to classify the different dynamical behaviors. As discussed in section S1.3 and in the main text, we have repeated this procedure for many different values of the state variables (species concentrations) and kinetic parameters to obtain a more complete picture of the system dynamics in the parameter space.

When a unique steady state exists, the sign of the largest real part of the eigenvalue associated with that solution allows us to classify that steady state as stable (represented by black in Figs.2b-d) or unstable. The same analysis is possible when three steady states coexist. Evaluating the Jacobian matrix and calculating the eigenvalues for each solution enabled classification of the possible dynamical behaviors. For the considered ranges of parameters, typically two stable and one unstable solution indicating a bistable dynamical behavior is reported (represented by red in Figs.2b-d). The borders separating these different regions correspond to different kind of transitions. The points belonging to the borders separating the cases when one or three solutions exist are called saddle nodes bifurcation.

### S2.2. Bifurcation diagrams in low dimension (2D)

To show that bistability exists for a wide range of parameters and is not specific to the reference parameter sets chosen for simulation, we generated a series of 2D bifurcation diagrams using the software XPPaut. XPPaut finds fixed points of a system and tracks them as a parameter is varied, giving lines along which equilibrium points exist for the set of parameters being examined (Ermentrout, 2002; XPPAUT). The system is bistable for all values of the parameters on the x and y axes in the area enclosed by these lines, marked in red (e.g. see Fig.2b,c). The system is monostable for all parameter values outside the enclosed area, marked in black.

### S2.3. Multi-dimensional analysis of model dynamics to probe the parameter space

Although the dynamical properties of a dynamic system are often judged based on conventional 2D bifurcation analysis (e.g. using XPPaut and AUTO (Ermentrout, 2002; XPPAUT) and presented on 2D bifurcation diagrams, the fact that usually only two parameters are varied at the same time (while remaining parameters are set at fixed values) poses concrete limitation in our effort to obtain a global, multi-dimensional picture of the systems dynamics.

To overcome this limitation, we employ an ensemble approach called DYVIPAC (Nguyen et al., 2015) where multiple model parameters can be simultaneously sampled; and thus the dynamic behaviour of the studied system can be probed over a much wider region of the multi-dimensional parameter space. Importantly, we adapt the Parallel Coordinates graphs (Inselberg, 1985) as a new way to effectively represent the multi-dimensional data from the ensemble dynamical analysis (Nguyen et al., 2015).

First, we select the parameters for simultaneous analysis, the number of which is not limited (typically at least three). Next, parameter sampling algorithms (e.g. Monte Carlo) are carried out to sample a large number of parameter sets (often tens of thousands) over defined ranges for each parameter in a random, unbiased manner from uniform or loguniform distributions. These defined parameter ranges typically span physiologically relevant values for each parameter, but can also be relaxed from biological constraints to take any plausible value range for exploration purpose. For each parameter set generated, assessment of the network's dynamic property at that set is conducted based on linear stability analysis outlined in section S2.1 above. This analysis enables the generic subdivision of the multi-parameter space into regions with distinct dynamic behaviours, including but not limited to monostability, bistability and oscillations. For our Rac1-PAK-RhoA model, monostable and bistable dyamics are typically obtained. Subsequently, results from such dynamics classification can be effectively visualised in a multi-parameter manner using Parallel Coordinates plots, as illustrated in in Fig.2d and Fig.S4. These plots can be loosely considered as *multi-dimensional bifurcation plots* of systems dynamics, analogous to the 2D bifurcation diagram produced by conventional methods, but for multiple model parameters.

We repeated the above 5D analysis for different sets of the kinetic parameters varied around their assumed physiological values. For multiple parameter sets where 10 kinetic parameters (Km2, Km8, Km12, k3, k2, k5, k7, k8, k10 and k12) are sampled randomly within 50% deviation range around their nominal values, simulations show that the identified bistable region is not significantly affected by these variations (Fig. S5). When we fixed the protein abundances in our model at the values determined in MDA-MB-231 cells and varied the kinetic parameters (in multidimensional kinetic parameter space using DYVIPAC (Nguyen et al., 2015)), we observed that bistability occurs over large ranges of these parameters (Fig. S6).

## S3. Dimensionless model of the Rac1-PAK-RhoA system

### S3.1. Transformation to the dimensionless model

To minimize issues related to parameter dependencies when analyzing the Rac1-PAK-RhoA systems dynamic properties, we transformed the original ODE system given in Table S2 to a dimensionless form where the protein abundance variables are appropriately scaled to selected Michealis-Menten constants (Kms) or binding affinities (Kds). This resulted in the independent parameter combinations that could be used to determine the bistability region. We then analysed this dimensionless model using DYVIPAC, varying the independent parameters over the ranges spanning three orders of magnitudes of our assumed original parameter values that were based on experimentally measured and estimated values.

For derivation of the dimensionless model, we introduced the following dimensionless variables:

$$\mathrm{dGEF\text{-}H1}=\frac{\mathrm{GEF\text{-}H1}}{K_{m12}};\ \mathrm{dpGEF\text{-}H1}=\frac{\mathrm{pGEF\text{-}H1}}{K_{m12}};$$
$$\mathrm{dIPA\text{-}3}=\frac{\mathrm{IPA\text{-}2}}{K_{m8}};\ \mathrm{dIPA\text{-}3\text{-}iPAK}=\frac{\mathrm{IPA\text{-}3\text{-}iPAK}}{K_{m8}};$$
$$\mathrm{diPAK}=\frac{\mathrm{iPAK}}{K_{m8}};\ \mathrm{dpPAK}=\frac{\mathrm{pPAK}}{K_{m8}};$$
$$\mathrm{d14\text{-}3\text{-}3}=\frac{\mathrm{14\text{-}3\text{-}3}}{K_{d1110}};\mathrm{d14\text{-}3\text{-}3\text{-}pGEF\text{-}H1}=\frac{\mathrm{14\text{-}3\text{-}3\text{-}pGEF\text{-}H1}}{K_{m12}};$$
$$\mathrm{dRac1GDP}=\frac{\mathrm{Rac1GDP}}{K_{d43}};\mathrm{dRac1GTP\text{-}iPAK}=\frac{\mathrm{Rac1GDP\text{-}iPAK}}{K_{d43}};\mathrm{dRac1GTP}=\frac{\mathrm{Rac1GTP}}{K_{d43}};$$
$$\mathrm{dRac1GTP\text{-}pPAK}=\frac{\mathrm{Rac1GDP\text{-}pPAK}}{K_{m8}};$$
$$\mathrm{dRhoAGDP}=\frac{\mathrm{RhoAGDP}}{K_{m2}}\ \text{and}\ \mathrm{dRhoAGTP}=\frac{\mathrm{RhoAGTP}}{K_{m2}}$$

where the prefix "d" indicates dimensionless variables; $K_{d1110}=k_{11}/k_{10}$ and $K_{d43}=k_4/k_3$ are the dissociation constants of the respective binding reactions. Given these new variables, the conservation laws for the new dimensionless model now becomes:

$$[Rac1{-}GTP]+[Rac1{-}GDP]+[Rac1{-}GTP{-}iPAK]+\frac{K_{m8}}{K_{m12}}[Rac1{-}GTP{-}pPAK]=\mathrm{dRac1_{TOT}}$$
$$[IPA{-}3]+[IPA{-}3{-}iPAK]=\mathrm{dIPA\text{-}3_{TOT}}$$
$$[iPAK]+[IPA{-}3{-}iPAK]+\frac{K_{m8}}{K_{m12}}[Rac1{-}GTP{-}iPAK]+[Rac1{-}GTP{-}pPAK]+[pPAK]=\mathrm{dPAK_{TOT}}$$
$$\frac{K_{d1110}}{K_{m12}}[14{-}3{-}3]+[14{-}3{-}3{-}pGEF{-}H1]=\mathrm{d14{-}3{-}3_{TOT}}$$
$$[GEF{-}H1]+[pGEF{-}H1]+[14{-}3{-}3]+[14{-}3{-}3{-}pGEF{-}H1]=\mathrm{dGEF\text{-}H1_{TOT}}$$
$$[RhoA{-}GTP]+[RhoA{-}GDP]=\mathrm{dRhoA_{TOT}}$$

where $\mathrm{dRac1_{TOT}}=\mathrm{Rac1_{TOT}}/K_{d43};\mathrm{dIPA\text{-}3_{TOT}}=\mathrm{IPA\text{-}3_{TOT}}/K_{m8};\mathrm{dPAK_{TOT}}=\mathrm{PAK_{TOT}}/K_{m8};$
$\mathrm{d14\text{-}3\text{-}3_{TOT}}=\mathrm{14\text{-}3\text{-}3_{TOT}}/K_{m12};\mathrm{dGEF\text{-}H1_{TOT}}=\mathrm{GEF\text{-}H1_{TOT}}/K_{m12}$ and $\mathrm{dRhoA_{TOT}}=\mathrm{RhoA_{TOT}}/K_{m2};$

are the dimensionless total species concentrations.

After substituting these new independent variables into the original ODE system, the ODEs of the dimensionless model have the following forms:

dGEFH1' = ((Vmax9/Km12) *dpGEFH1)/((Km9/Km12) + dpGEFH1) - ((k8*Km8/Km12) *dGEFH1 *dpPAK)/((Km8/Km12) + dGEFH1)

dIPA3iPAK' = -k15 *dIPA3iPAK + (k14*Km8) *dIPA3 *diPAK

dpPAK' = -k16 *dpPAK + k7 *dRac1GTPpPAK

dprotein1433pGEFH1' = (k10*Kd1110) *dpGEFH1 *dprotein1433 - (k10*Kd1110) *dprotein1433pGEFH1

dRac1GTP' = ((k1*Km1/Kd43) *(Rac1GEF/Km1) *dRac1GDP)/(Km1/Kd43 + dRac1GDP) - (k3*Km8) *diPAK *dRac1GTP + (k3*Kd43) *dRac1GTPiPAK + (k7*Km8/Kd43) *dRac1GTPpPAK - ((k2*Km2/Kd43) *(Rac1GAP/Km12) *dRac1GTP *(1/Km2 + (beta32 *dRhoAGTP)/K32))/((Km2/Kd43 + dRac1GTP) *(1/Km2 + dRhoAGTP/K32))

dRac1GTPiPAK' = (k3*Km8) *diPAK *dRac1GTP - k4 *dRac1GTPiPAK - k5 *dRac1GTPiPAK + (k6*Km8/Kd43) *dRac1GTPpPAK

dRac1GTPpPAK' = (k5*Kd43/Km8) *dRac1GTPiPAK - (k6 + k7) *dRac1GTPpPAK

dRhoAGDP' = -(((k12*Km12/Km2) *dGEFH1 *dRhoAGDP)/(Km12/Km2 + dRhoAGDP)) + ((k13*Km13/Km2) *(RhoAGAP/Km13) *dRhoAGTP)/(Km13/Km2 + dRhoAGTP)

and (nominal) parameter values:

k1 = 0.06; Rac1GEF = 8.5; Km1 = 130; k2 = 0.505; Rac1GAP = 15; Km2 = 200; beta32 = 130; K32 = 1650; k3 = 0.0001; k4 = 0.0002; k5 = 0.0009; k6 = 0.00035; k7 = 0.0004; k8 = 0.505; Km8 = 30; Vmax9 = 5.05; Km9 = 20; k10 = 0.0001; k11 = 0.001; k12 = 0.9; Km12 = 170; k13 = 0.06; Km13 = 10; RhoAGAP = 10; k14 = 0.001; k15 = 0.01; k16 = 0.0005; Kd43 = k4/k3; Kd1110 = k11/k10;

### S3.2. Bistability analysis of the dimensionless model using DYVIPAC

The transformation of the original model into a dimensionless model not only reduced the model's dimension but also resulted in the independent parameter combinations that determine the bistability region. We carried out dynamical analysis on the dimensionless model using DYVIPAC (Nguyen et al., 2015), varying the dimensionless variables over ranges spanning three orders of magnitudes surrounding the nominal parameter values (shown in S3.1) based on experimentally measured and estimated values. As shown in Fig. S7, bistability is still observed over these large ranges of the new independent parameter values, confirming that bistability is also a dominant feature of the dimensionless system.
Note that bimodal distributions may arise in a number of situations: a purely stochastic genetic switch (Acar et al., 2008), a bistable system with stochastically induced transitions (Samoilov et al., 2005), noisy networks with the sigmoidal response function (Niepel et al., 2009; Ochab-Marcinek and Tabaka, 2010), or even as a result of heterogeneous deterministic oscillations when protein abundances vary between isogenic cells (Dobrzynski, 2012). In our case, the combined modelling and validation experimental data strongly suggest that bimodality arises from bistability.

## S4. Modelling the effects of additional positive feedbacks

### S4.1. Simplified model of the Rac1-PAK-RhoA system

Rac1 and RhoA are embedded in a wider network of interactions, which were not included in our original models. For example, there is feedback from PAK to upstream Rac1 via the protein Cool-2 (cloned out of library-2, also known as α-PIX) that form a positive feedback between Rac1 and PAK. Specifically, when in dimeric form, Cool-2 can act as a specific GEF for Rac1. Upon dissociation into monomers, a process facilitated by PAK, it can act as a GEF for both Rac1 and Cdc42. This generates a positive effect from PAK to Rac1, closing a positive feedback between Rac1 and PAK ((Baird et al., 2005; Feng et al., 2002). However, adding the positive feedback from PAC to Rac1 to the system with existing double negative feedback only enlarges the bistability range, but did not significantly affect the network behaviour, Fig. S14. Similarly, adding Rac1 auto-regulatory positive loop that is PAK-independent also only intensifies bistable behaviour, but did not significantly alter bistability dynamics (Tsyganov et al., 2012), Fig. S14. Also, the incorporation of GDP dissociation inhibitors (GDIs) in the model only modifies the parameter range where bistability exists, but bistability remains a feature of the Rac1-RhoA system (Nikonova et al., 2013).

In this section, we describe the formulation of a simplified model of the Rac1-PAK-RhoA network which is aimed to facilitate the analysis of the roles of additional positive feedbacks. This model allows incorporation of either a Rac1-PAK positive feedback as well as PAK-independent Rac1 autoregulatory positive feedback loop into the Rac1–RhoA double negative feedback. The schematic diagram of this model is given in Fig. S12a. Model formulation and notation convention follow that described in our previous study (Tsyganov et al., 2012).

Model's ODEs:

$g1p'(t) = a_{11}*a_{21}*w_1 - a_{32}*w_2;$
$g2p'(t) = a_{13}*w_3 - w_4;$
$g3p'(t) = a_{25}*w_5 - w_6;$

Here for convenience, g1p, g2p, g3p represent the dimensionless concentrations of the active Rac1-GTP, active pPAK and active RhoA-GTP, respectively (normalized by the respective total abundances).

The basic reaction rates $w_i$ (i=1-6) are defined as:

$$w_1 = \frac{r_1 \cdot (1 - \mathrm{g1p}(t)) / m_1}{1 + (1 - \mathrm{g1p}(t)) / m_1};$$

$$w_2 = \frac{r_2 \cdot \mathrm{g1p}(t) / m_2}{1 + \mathrm{g1p}(t) / m_2};$$

$$w_3 = \frac{r_3 \cdot (\frac{1 - \mathrm{g2p}(t)}{1 + \mathrm{Inh}}) / m_3}{1 + (\frac{1 - \mathrm{g2p}(t)}{1 + \mathrm{Inh}}) / m_3};$$

$$w_4 = \frac{r_4 \cdot \mathrm{g2p}(t) / m_4}{1 + \mathrm{g2p}(t) / m_4};$$

$$w_5 = \frac{r_5 \cdot (1 - \mathrm{g3p}(t)) / m_5}{1 + (1 - \mathrm{g3p}(t)) / m_5};$$

$$w_6 = \frac{r_6 \cdot \mathrm{g3p}(t) / m_6}{1 + \mathrm{g3p}(t) / m_6};$$

where the parameter Inh represents the normalized concentration of the PAK inhibitor. The modifier terms that describe respective feedbacks are defined as below:

$$a_{11} = \frac{1 + b_{11} \cdot \mathrm{g1p}(t) / m_{11}}{1 + \mathrm{g1p}(t) / m_{11}};$$

$$a_{13} = \frac{1 + b_{13} \cdot \mathrm{g1p}(t) / m_{13}}{1 + \mathrm{g1p}(t) / m_{13}};$$

$$a_{21} = \frac{1 + b_{21} \cdot \mathrm{g2p}(t) / m_{21}}{1 + \mathrm{g2p}(t) / m_{21}};$$

$$a_{25} = \frac{1 + b_{25} \cdot \mathrm{g2p}(t) / m_{25}}{1 + \mathrm{g2p}(t) / m_{25}};$$

$$a_{32} = \frac{1 + b_{32} \cdot \mathrm{g3p}(t) / m_{32}}{1 + \mathrm{g3p}(t) / m_{32}};$$

Here $a_{13}$, $a_{25}$ and $a_{31}$ describes the positive regulation of PAK (g2p) by active Rac1 (g1p), negative regulation of RhoA (g3p) by active PAK (g2p) and negative regulation of Rac1 (g1p) by active RhoA (g3p), forming the Rac1-PAK-RhoA double-negative feedback loop.

On the other hand, $a_{21}$ describes possible positive regulation of Rac1 by PAK, forming a positive feedback between Rac1 and PAK ($b_{21} > 1$). And $a_{11}$ describes possible auto-positive regulation of Rac1, forming a autoregulatory positive feedback of Rac1 that is PAK independent ($b_{11} > 1$).

Nominal parameter values used for simulations:

$r_1 = 3.122$, $r_2 = 0.074$, $r_3 = 30$, $r_4 = 25$, $r_5 = 12$, $r_6 = 11.3$,
$m_1 = 0.236$, $m_2 = 0.00483$, $m_3 = 0.297$, $m_4 = 0.269$, $m_5 = 0.030$, $m_6 = 0.045$,
$b_{13} = 150$, $m_{13} = 1$,
$b_{32} = 200$, $m_{32} = 1$,
$b_{25} = 0.5$`, $m_{25} = 1$,
Inh = 5

### S4.2. Examining the effect of added positive feedbacks

The model simplification and formulation described above allowed us to clearly define the strength of each feedback loops and this easily modulate them *in silico*. In agreement with our previous work (Tsyganov et al., 2012) which investigates a large number of two-tiered GTPase cascade topologies, we found that in this three-tiered Rac1-PAK-RhoA system, both the double negative feedback mediated via Rac1-PAK-RhoA and the Rac1-

PAK positive feedback, in principle, can bring bistability on their own. However, adding the positive feedback to the system with existing double negative feedback only enlarges the bistability range, but did not significantly affect the network behaviour (Fig.S12c). Similarly, adding a hypothetical Rac1 auto-regulatory positive that is PAK-independent also only intensify bistability but did not significantly alter bistability dynamics.

## S5. Materials and Methods

***Cells and reagents.*** Cells were cultured in DMEM supplemented with 2 mM glutamine and 10% foetal calf serum. Plasmids were transfected with Lipofectamine2000 using the manufacturer's instructions (Invitrogen, UK). Plasmids for $PAK^i$ and $PAK^i$-2m-eGFP and $PAK^i$-2m were kindly provided by Debbie Yablonski, GST-Rhotekin-RBD by Mike Olson, GST-Pak-CRIB by Piero Crespo. Antibodies for RhoA were from Santa Cruz (Clane, UK); Rac1 from Millipore (Watford UK), for EGFP from Cell Signalling (Hitchin, UK), IPA-3 was from Merk Millipore (Watford UK).

***Cell treatment, lysis and pulldown assays.*** Cells seeded in collagen-coated plates were either pretreated with 15 μM IPA-3 for 20 minutes or incubated with IPA-3 (0-15 μM) for 40 minutes. The IPA-3 containing media from the pretreated cells was removed and the cells were washed 2 times with 10% FCS DMEM and subsequently incubated in 10% FCS DMEM containing IPA-3 (0-15 μM) for an additional 20 minutes in 10 % FCS-DMEM. Cells were lysed in ice-cold lysis buffer (20 mM HEPES pH7.5, 150 mM NaCl, 1% NP40, 2 mM EDTA) supplemented with protease inhibitors (1 mM PMSF, 5 μg/ml leupeptin, 2.2 μg/ml aprotinin, 2 mM sodium fluoride) and 10 mM $MgCl_2$ (only in pull-down assays). Lysates were cleared of debris by centrifugation at 20,000 g for 10 minutes in a benchtop centrifuge. Cleared lysates were incubated with either 5 μl GST-Rhotekin- or GST-Pak-CRIB-beads for 30 min at 4ºC under end-to-end rotation (for pull-down assays) or boiled in Laemmli buffer (for RhoA/Rac1 input). The beads were washed, boiled in Laemmli buffer and Western blotted. The Western blot bands were quantified using ImageJ. Graphs represent RhoA-GTP/input RhoA, Rac1-GTP/input Rac1.

***Motility assays.*** MDA-MB-231 cells were seeded at 15,000 cells/ml in collagen-coated 12-well plates. After treatment with IPA-3, cells were imaged using a Zeiss Axiovert 200M at 10x, using Andor iQ software, over 12 hours in a temperature and $CO_2$-controlled environmental chamber. Images were taken every 20 minutes. Individual cells were manually tracked using the Manual Tracking Plugin in ImageJ 1.44o software package. The Manual Tracking plugin gives the position (in pixels) of each cell at each time step and uses this to calculate the speed of each cell per time step (20 minutes). The paths taken by 25 cells under each treatment were plotted in Fig. 4a from the position readouts using MATLAB (Inc., 2010). The average speed of individual cells was calculated over 12 hours and these were used to calculate the average speed per well. For wound healing assays, cells were plated in 6-well dishes and grown to confluence. The cell layer was subsequently scratched with a pipette tip. Three positions were marked on each plate and photographed immediately and after 18h. The wound closure was measured manually on the images.

***Cell morphology assays.*** 0.5 ml of rat-tail collagen I was polymerised in 12-well dishes. 500 MDA-MB-231 cells were seeded and were let to adhere and invade into the collagen gel. 24 hours after seeding the cells were either pretreated with 7.5 μM IPA-3 for 20 minutes or incubated with IPA-3 (0-7.5 μM). The IPA-3 containing media from the pretreated cells was removed and the cells were washed 2 times by incubating the collagen plug for 2 minutes with 10% FCS DMEM and subsequently incubated in 10% FCS DMEM containing IPA-3 (0-7.5 μM). The cells were imaged 24 hours after the treatment by using an Incucyte ZOOM. Cell masks of individual cells were detected using the Incucyte analysis software and cell roundness was measured using ImageJ.

***Actin dynamics imaging.*** *LifeAct-mCherry probe* was delivered to the cells using pHIV lentiviral transfection. This probe was generously provided by Prof. Olivier Pertz (Department of Biomedicine, University of Basel). Cells were seeded and treated as described above for motility assays. The probe expressing cells were imaged at 15 sec intervals for 1 hour, using a Nikon Plan Apo 40x/1.5 DIC oil objective on a spinning-disk laser confocal Nikon microscope with Andor iXonEM+ EMCCD camera, resulting in an effective pixel size of 234 nm. The 561 nm excitation laser and 610 nm emission filter were used. Montage images and movies were created using ImageJ software.

***Cellular pMLC imaging.*** 2000 MDA-MB-231 cells were seeded in collagen I coated polymer optical-bottomed 96-well dishes (Thermo Fisher). 24 hours later cells were either pretreated with 7.5 μM IPA-3 for 20 minutes or incubated with IPA-3 (0-7.5 μM) for 80 minutes. The IPA-3 containing media from the pretreated cells was removed and the cells were washed 2 times with 10% FCS DMEM and subsequently incubated in 10% FCS DMEM containing IPA-3 (0-7.5 μM) for an additional 60 minutes in 10 % FCS-DMEM. Post-treatment the cells were fixed in 3.7% Formaldehyde (PIPES 100 mM pH 6.8, EGTA 10 mM, MgCl2 10 mM, Triton X-100 0.2 %) for 10 minutes. F-actin was stained with Phalloidin 594 nm (Molecular Probes, 1:250 dilution), nucleus by

Hoechst (Invitrogen, 1:5000 dilution), pMLC by pSer19-Myosin Light Chain 2 (Cell signalling, 1/200 dilution) and Goat-anti-Mouse 488 nm Alexa secondary (Invitrogen, 1/200 dilution) and the cell body with HCS cell mask deep red (Invitrogen, 1:150,000 dilution). Cells were imaged on an ImageXpress Micro widefield microscope and images analysed using the MetaXpress Custom Module Editor (Molecular Devices). Briefly, using a local thresholding image analysis technique, we generated binary masks of the nuclei, whole cell mask (derived from the Phalloidin labelling) and cell body mask (from the HCS stain) for each cell. Subtraction of the cell body mask from the whole cell mask gave us a third mask of the lamellipodia area of each cell. Subtraction of the nuclear mask from the whole cell mask allowed us to measure cytoplasmic Phalloidin staining (Supplemental Figure S11a). We then measured the integrated mean fluorescent intensity of the pMLC or phalloidin labelling in each of the masks measuring >2000 cells per treatment.

***Quantitative mass spectrometry.*** MDA-MB-231 cells were lysed in 1% SDS and lysates were washed, reduced, alkylated, digested with trypsin and analysed on a Q-Exactive mass spectrometer as previously reported (Farrell et al., 2014). Proteins were identified and quantified by using the MaxQuant software suite (Cox et al., 2011) by searching against the human swissprot database, with Carbamylations of Cysteine as fixed and N-terminal acetylation and Methionine oxidation as variable modifications. The absolute cellular protein concentration was calculated by the Perseus software suite with the proteome ruler method (Wisniewski et al., 2014). Expression data is summarised in Table S6 and the data are available via ProteomeXchange with identifier PXD003213.

***Determining retention time of cellular IPA-3.*** MDA-MB-231 cells were seeded in collagen coated 6-well dishes and incubated with 7.5 µM IPA-3 for 0-20 minutes. The IPA-3 containing media was removed and the cells were washed three times with ice-cold PBS. Inter-cellular IPA-3 was extracted by scraping the cells with cold methanol. Samples were cleared of cell debris by centrifugation and the methanol was removed with a rotary vacuum concentrator. IPA-3 was detected on a Thermo QExactive using ZIC pHILIC 20 x 2.1 mm guard and 150 x 4.6 mm column (SeQuant) with a 10 minute gradient from 90% to 5% acetonitrile against 20 mM ammonium carbonate, in negative SIM mode, monitoring 348.6 to 349.5 m/z at resolution 70,000, AGC target 3e6 and max IT 200 milliseconds. Peak detection and integration was performed using Thermo Xcalibur 3.0.63.

## Supplemental References:


Acar, M., Mettetal, J.T., and van Oudenaarden, A. (2008). Stochastic switching as a survival strategy in fluctuating environments. Nature genetics *40*, 471-475.

Baird, D., Feng, Q., and Cerione, R.A. (2005). The Cool-2/alpha-Pix protein mediates a Cdc42-Rac signaling cascade. Curr Biol *15*, 1-10.

Beck, M., Schmidt, A., Malmstroem, J., Claassen, M., Ori, A., Szymborska, A., Herzog, F., Rinner, O., Ellenberg, J., and Aebersold, R. (2011). The quantitative proteome of a human cell line. Mol Syst Biol *7*, 549.

Bokoch, G.M. (2003). Biology of the p21-activated kinases. Annu Rev Biochem *72*, 743-781.

Cox, J., Neuhauser, N., Michalski, A., Scheltema, R.A., Olsen, J.V., and Mann, M. (2011). Andromeda: A Peptide Search Engine Integrated into the MaxQuant Environment. Journal of proteome research *10*, 1794-1805.

Deacon, S.W., Beeser, A., Fukui, J.A., Rennefahrt, U.E., Myers, C., Chernoff, J., and Peterson, J.R. (2008). An isoform-selective, small-molecule inhibitor targets the autoregulatory mechanism of p21-activated kinase. Chem Biol *15*, 322-331.

Dobrzynski, M., Fey, D., Nguyen L. K. & Kholodenko, B. N. (2012). Bimodal Protein Distribution in Heterogeneous Oscillating Systems. . In Computational Methods in Systems Biology Lecture Notes in Computer Science, D.H.M. Gilbert, ed. (Springer-Verlag Berlin Heidelberg), pp. 17-28.

Ermentrout, B. (2002). Simulating, Analyzing, and Animating Dynamical Systems: A Guide to XPPAUT for Researchers and Students (Philadelphia, USA: SIAM).

Farrell, J., Kelly, C., Rauch, J., Kida, K., Garcia-Munoz, A., Monsefi, N., Turriziani, B., Doherty, C., Mehta, J.P., Matallanas, D.*, et al.* (2014). HGF induces epithelial-to-mesenchymal transition by modulating the mammalian hippo/MST2 and ISG15 pathways. Journal of proteome research *13*, 2874-2886.

Feng, Q., Albeck, J.G., Cerione, R.A., and Yang, W. (2002). Regulation of the Cool/Pix proteins: key binding partners of the Cdc42/Rac targets, the p21-activated kinases. J Biol Chem *277*, 5644-5650.

Inc., T.M. (2010). MATLAB (Natick, Massachusetts).

Inselberg (1985). The Plane with Parallel Coordinates. Visual Computer *1*, 69–91.

Kholodenko, B.N., Demin, O.V., Moehren, G., and Hoek, J.B. (1999). Quantification of short term signaling by the epidermal growth factor receptor. The Journal of biological chemistry *274*, 30169-30181.

Krendel, M., Zenke, F.T., and Bokoch, G.M. (2002). Nucleotide exchange factor GEF-H1 mediates cross-talk between microtubules and the actin cytoskeleton. Nature cell biology *4*, 294-301.

Nagaraj, N., Wisniewski, J.R., Geiger, T., Cox, J., Kircher, M., Kelso, J., Paabo, S., and Mann, M. (2011). Deep proteome and transcriptome mapping of a human cancer cell line. Mol Syst Biol *7*, 548.

Nguyen, L.K., Degasperi, A., Cotter, P., and Kholodenko, B.N. (2015). DYVIPAC: an integrated analysis and visualisation framework to probe multi-dimensional biological networks. Scientific Reports *(in press)*.
Niepel, M., Spencer, S.L., and Sorger, P.K. (2009). Non-genetic cell-to-cell variability and the consequences for pharmacology. Current opinion in chemical biology *13*, 556-561.
Nikonova, E., Tsyganov, M.A., Kolch, W., Fey, D., and Kholodenko, B.N. (2013). Control of the G-protein cascade dynamics by GDP dissociation inhibitors. Mol Biosyst *9*, 2454-2462.
Ochab-Marcinek, A., and Tabaka, M. (2010). Bimodal gene expression in noncooperative regulatory systems. Proceedings of the National Academy of Sciences of the United States of America *107*, 22096-22101.
Saito, K., Ozawa, Y., Hibino, K., and Ohta, Y. (2012). FilGAP, a Rho/Rho-associated protein kinase-regulated GTPase-activating protein for Rac, controls tumor cell migration. Mol Biol Cell *23*, 4739-4750.
Samoilov, M., Plyasunov, S., and Arkin, A.P. (2005). Stochastic amplification and signaling in enzymatic futile cycles through noise-induced bistability with oscillations. Proceedings of the National Academy of Sciences of the United States of America *102*, 2310-2315.
Sanz-Moreno, V., Gadea, G., Ahn, J., Paterson, H., Marra, P., Pinner, S., Sahai, E., and Marshall, C.J. (2008). Rac activation and inactivation control plasticity of tumor cell movement. Cell *135*, 510-523.
Tsyganov, M.A., Kolch, W., and Kholodenko, B.N. (2012). The topology design principles that determine the spatiotemporal dynamics of G-protein cascades. Mol Biosyst *8*, 730-743.
Viaud, J., and Peterson, J.R. (2009). An allosteric kinase inhibitor binds the p21-activated kinase autoregulatory domain covalently. Mol Cancer Ther *8*, 2559-2565.
Wisniewski, J.R., Hein, M.Y., Cox, J., and Mann, M. (2014). A "proteomic ruler" for protein copy number and concentration estimation without spike-in standards. Molecular & cellular proteomics : MCP *13*, 3497-3506.
XPPAUT http://www.math.pitt.edu/~bard/xpp/xpp.html.
Zenke, F.T., Krendel, M., DerMardirossian, C., King, C.C., Bohl, B.P., and Bokoch, G.M. (2004). p21-activated kinase 1 phosphorylates and regulates 14-3-3 binding to GEF-H1, a microtubule-localized Rho exchange factor. J Biol Chem *279*, 18392-18400.
Zhao, L., Kroenke, C.D., Song, J., Piwnica-Worms, D., Ackerman, J.J., and Neil, J.J. (2008). Intracellular water-specific MR of microbead-adherent cells: the HeLa cell intracellular water exchange lifetime. NMR Biomed *21*, 159-164.
Zhao, Z.S., and Manser, E. (2012). PAK family kinases: Physiological roles and regulation. Cell Logist *2*, 59-68.
